# Supplementary material for: Ring walking as a regioselectivity control element in Pd-catalyzed C-N cross-coupling
Source: Nat Commun. 2022 May 24;13:2869. doi: 10.1038/s41467-022-30255-1 (PMC9130136; doi:10.1038/s41467-022-30255-1)
Supplement: Supplementary file 1 — Supplementary Information [file 41467_2022_30255_MOESM1_ESM.pdf]

SUPPLEMENTARY INFORMATION

Ring Walking as a Regioselectivity Control Element in Pd-Catalyzed C-N Cross Coupling

Madeleine C. Deem, Joshua S. Derasp, Thomas C. Malig, Kea Legard, Curtis P. Berlinguette* and Jason E. Hein*

Department of Chemistry, University of British Columbia, Vancouver, British Columbia, V6T 1Z3, Canada

* cberling@chem.ubc.ca, jhein@chem.ubc.ca

General information	2
Sampling platforms and parameters	4
Kinetic data	6
Assignment of intermediates in the synthesis of 6 and 17	19
COPASI modeling	21
Alternate substrates	25
Synthetic procedures	29
Crystallographic Data	35
Spectra	37
References	67

Supplementary methods

Chemical suppliers: THF and toluene were purchased from Millipore Sigma and dispensed from an MBraun solvent purification system (SPS) prior to use. **13** was synthesized according to literature procedure[1]. All other chemicals and solvents were purchased from Millipore Sigma, Ambeed, AK Scientific, Strem Chemicals, or Alfa Aesar and used without further purification.

Analytical equipment and methods: ^1H and ^{13}C NMR were recorded on a Bruker 300, AV-400, or 600 MHz spectrometer and were referenced to the residual solvent peaks (THF-d_8 1.72 and 3.58 ppm or DMSO-d_6 2.50 ppm for ^1H NMR and C_6D_6 128.06 ppm or THF-d_8 25.31 and 67.21 ppm or DMSO-d_6 39.52 ppm for ^{13}C NMR). ^1H NMR multiplicity was reported using the following abbreviations: br = broad, s = singlet, d = doublet, t = triplet, q = quartet, quint. = quintet, sext. = sextuplet, sept = septuplet, and m = multiplet. Integration values and coupling constants were reported in Hz. Flash chromatography was done using SiliCycle silica gel (40-63 µm, 230-400 mesh). Analysis *via* HPLC-MS was conducted using an Agilent 1200 HPLC equipped with: an Agilent G1379B degasser, G1312A binary pump, G1316A thermal column compartment, diode array detector, and a 6120 single quad mass spectrometer. Two HPLC methods were used (see below for detailed description of the HPLC methods). Data processing and analysis were carried out using both ChemStation (Agilent) and another proprietary third-party software.

Method 1:

Column:	Poroshell 120 EC-C18 2.7 µm, 2.1 x 50 mm	
Column Temperature:	30 °C	
Flow Rate:	0.650 mL/min	
Detection:	254 nm	
Acquisition Time:	8 min	
Mobile Phase:	Solvent A = 0.1% formic acid in water B = acetonitrile	
Mobile Phase Program:	Time<br>0.00 min<br>1.50 min<br>1.51 min<br>7.00 min<br>8.00 min	B%<br>30<br>50<br>80<br>100<br>100
Injection Volume:	0.75 µL	
Compound Name:	Retention time:	
Bis(4-methoxyphenyl)amine (**2**)	1.79 min	
Biphenyl	1.99 min	
Oxidative degradation amine byproduct	2.24 min	
2,2',7,7'-tetrabromo-9,9'-spirobi[fluorene] (**1**)	5.25 min	
Di-coupled intermediate, same ring (**4a**)	6.50 min	
Spiro-OMeTAD (**6**)	6.98 min	
Example Chromatogram		

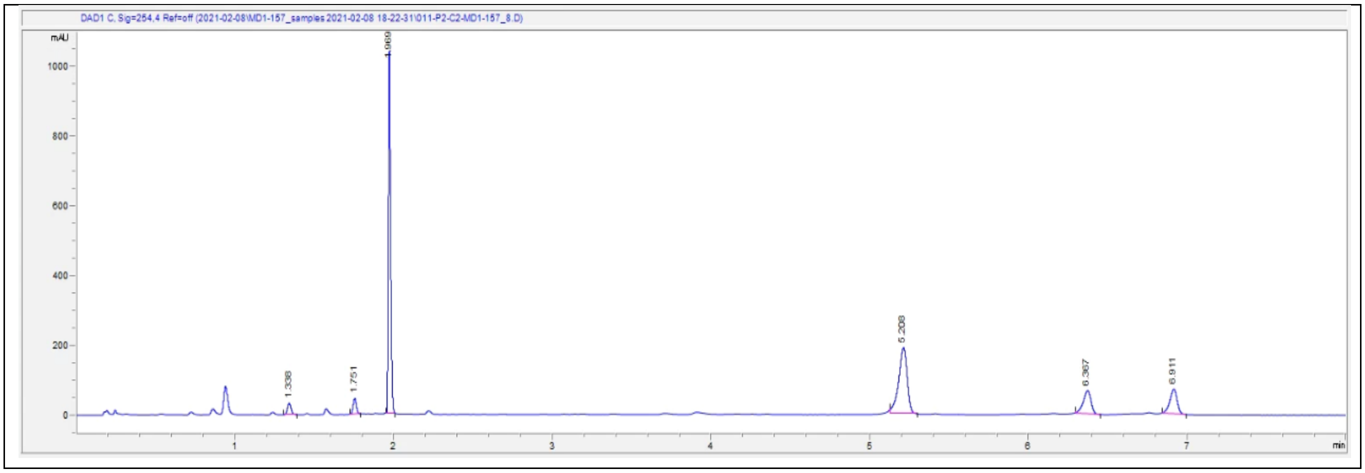

Method 2:

Column:	Poroshell 120 EC-C18 2.7 µm, 2.1 x 50 mm	
Column Temperature:	30 °C	
Flow Rate:	0.650 mL/min	
Detection:	254 nm	
Acquisition Time:	11 min	
Mobile Phase:	Solvent A = 0.1% formic acid in water B = acetonitrile	
Mobile Phase Program:	Time	B%
	0.00 min	30
	1.50 min	50
	10.00 min	95.3
Injection Volume:	0.75 µL	
Compound Name:	Retention time:	
Bis(4-methoxyphenyl)amine (**2**)	1.81 min	
Biphenyl	2.01 min	
Oxidative degradation amine byproduct	2.26 min	
2,2',7,7'-tetrabromo-9,9'-spirobi[fluorene] (**1**)	6.76 min	
Mono-coupled intermediate (**3**)	8.64 min	
Di-coupled intermediate, same ring (**4a**)	8.99 min	
Di-coupled intermediate, opp ring (**4b**)	9.69 min	
Tri-coupled intermediate (**5**)	9.85 min	
Spiro-OMeTAD (**6**)	10.24 min	

Example Chromatogram

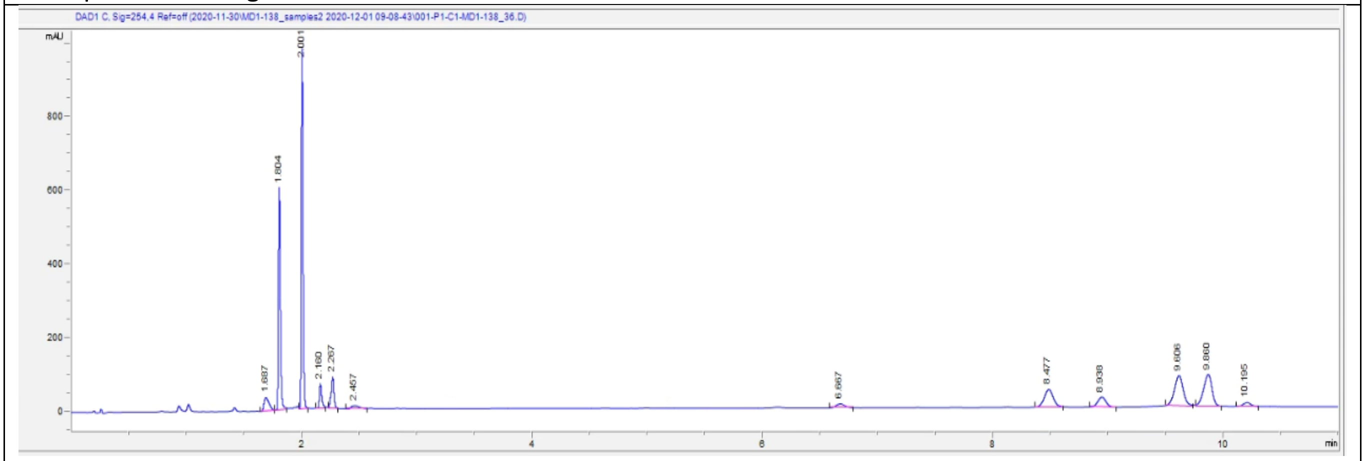

Sampling platforms and parameters

Automated sampling technology was used to gather time course data for the reactions in this study. Two types of sampling platforms were used: a Direct Inject (DI) system with online HPLC-MS and a Gilson liquid handler system with offline HPLC-MS. The DI system is shown in Supplementary Figure 1 and the Gilson-based system in shown in Supplementary Figure 2.

The different excess experiments for P(*t*Bu)$_3$ and the time course data for the alternate substrates were run using a DI system. The reactions with P(*t*Bu)$_3$ were conducted using an automated sampling platform set up inside of a glovebox. The different excess experiments for PEPPSI-*IPr*, RuPhos, and XantPhos were run on the Gilson-based system with offline HPLC-MS.

Direct Inject system with online HPLC-MS: Temporal HPLC data was obtained using a modified reaction monitoring platform similar to that which has been reported in our group previously.[2] All sampling events were executed by an Arduino microcontroller which was controlled *via* a Python script. The sampling sequence begins by actuating the EasySampler to extend the sampling pocket into the reaction mixture. The system is flushed with THF (2.0 mL at 1.0 mL/min). The pocket is then retracted, the valve position is switched bringing the sample loop in line with the EasySampler, and THF (standard volume = 0.75 mL) is delivered (default flow rate = 5.0 mL/min) filling the sample loop with the desired reaction aliquot. The injection valve is then switched, aligning the reaction aliquot with the HPLC pump and column to allow for online analysis. The sampling lines are flushed with THF (5.0 mL, 6.0 mL/min) followed by argon (90 sec) before reinitiating the sampling sequence. The Python sampling script is available on request.

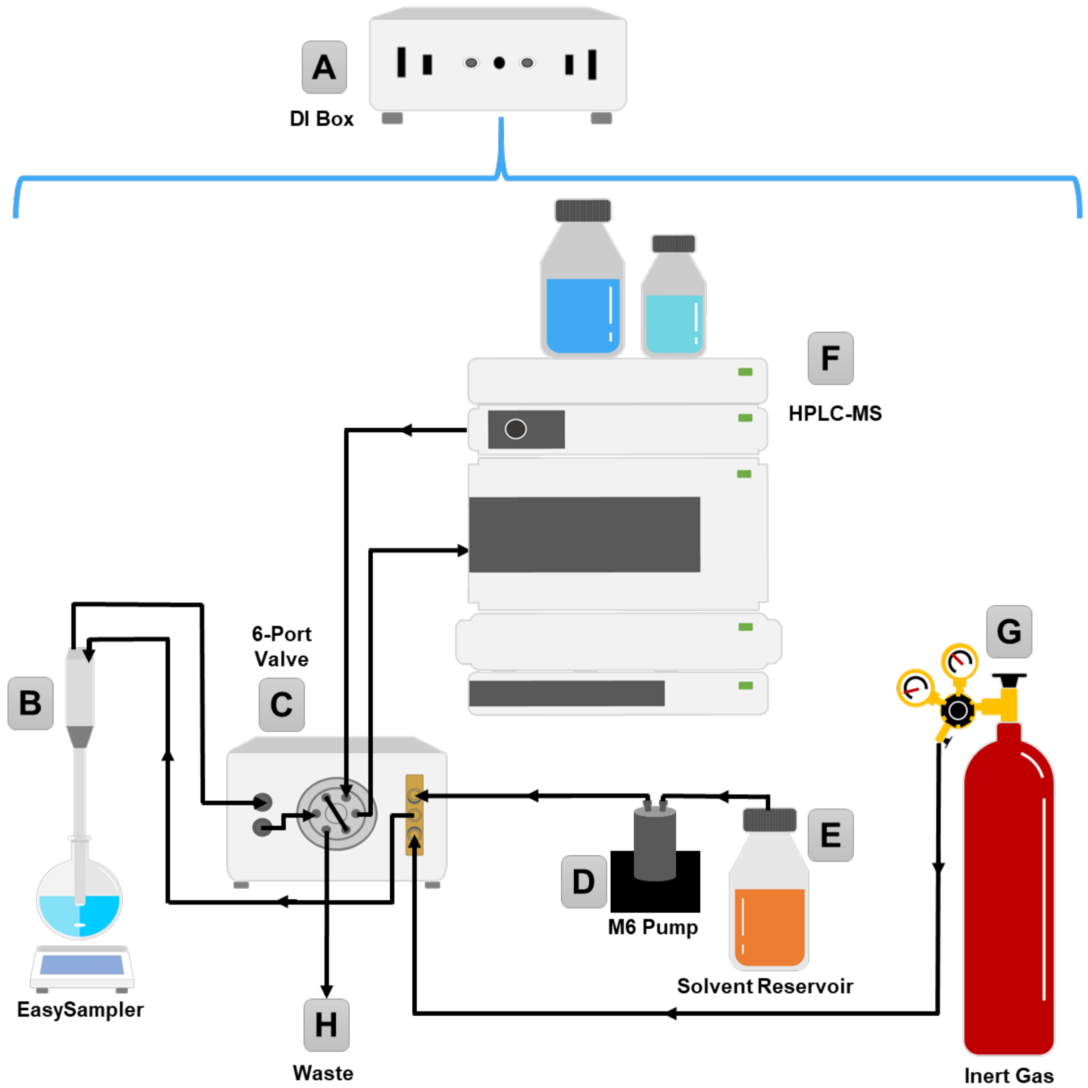

Supplementary Figure 1. Depiction of Direct Inject (DI) automated sampling system coupled with online HPLC-MS. **A**: DI box, which controls and coordinates the entire sampling sequence, run through Python script; **B**: EasySampler probe and reaction flask; **C**: 2-position 6-port injection valve with solvent/gas selector and pressure sensor; **D**: M-6 pump; **E**: push solvent reservoir (THF); **F**: HPLC-MS, see general information for specifications; **G**: argon tank.

Gilson sampling system with offline HPLC-MS: The sampling sequence begins with an argon purge (5 sec) of the system. The sampling pocket of the EasySampler extends and the lines are primed with THF (1.5 mL, 10.0 mL/min). The sampling pocket retracts and the dispensing needle on the Gilson liquid handler moves to an awaiting empty HPLC vial. Then, THF (0.75 mL, 10.0 mL/min) is pushed through the system to dispense the reaction aliquot into the HPLC vial. The dispensing needle is then returned to the waste position before THF (1.5 mL, 10.0 mL/min) and argon (2 min) are used to both clean and dry the lines. The HPLC vials containing the time point samples are transferred to an HPLC-MS and analyzed after the reaction finishes.

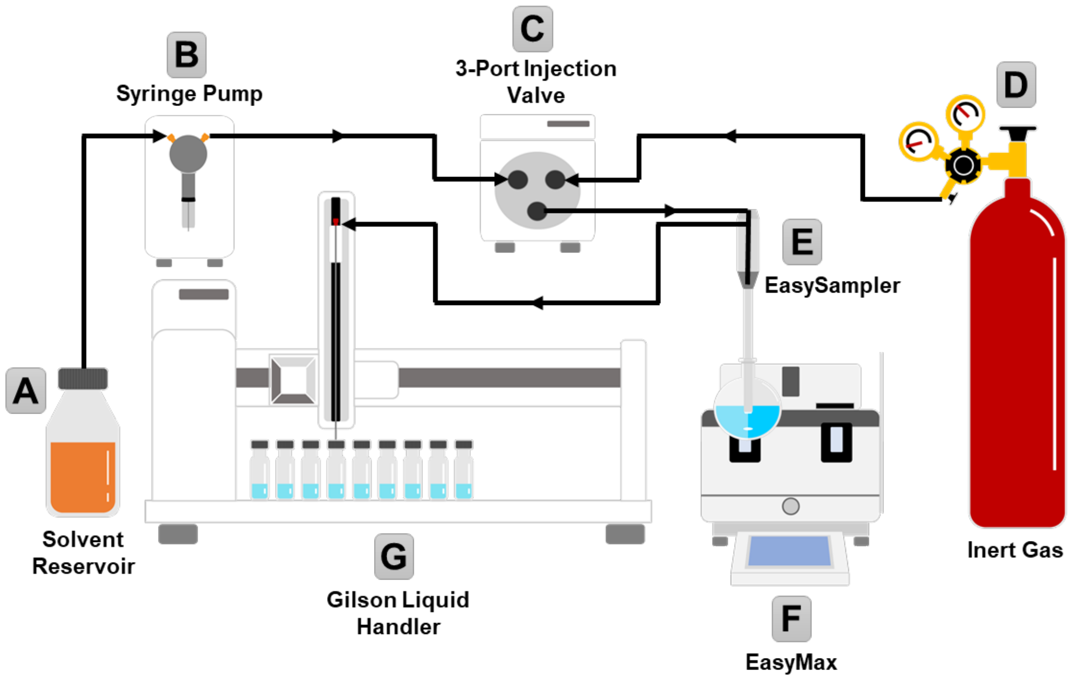

Supplementary Figure 2. Depiction of automated sampling system with Gilson liquid handler and offline HPLC-MS. **A**: solvent reservoir (THF) **B**: Cavro syringe pump; **C**: 3-port selector valve; **D**: argon tank; **E**: EasySampler probe; **F**: EasyMax reactor; **G**: 215 Gilson liquid handler.

Supplementary discussion

Kinetic data

The standard condition experiments for P(*t*Bu)$_3$, PEPPSI-*IPr*, RuPhos, and XantPhos were run in duplicate to ensure reproducibility of the reaction and the sampling system. The results from both duplicate experiments are shown below for P(*t*Bu)$_3$ (Supplementary Figure 3), PEPPSI-*IPr* (Supplementary Figure 4), RuPhos Pd G4 (Supplementary Figure 5), and XantPhos Pd G4 (Supplementary Figure 6). The results are displayed as overlays of the four reaction components (**1**, **2**, **4a**, and **6**) to better visualize the similarity of the two experiments. All of the standard conditions experiments displayed excellent reproducibility, demonstrating the robustness of both the sampling technology and the reaction. However, **2** was analytically unstable in the offline HPLC samples resulting in irreproducible overlays of **2** (example of poor amine reproducibility show in Supplementary Figure 6). Thus, overlays of **2** were not considered in the kinetic experiments. All subsequent kinetic experiments were reported as single data sets.

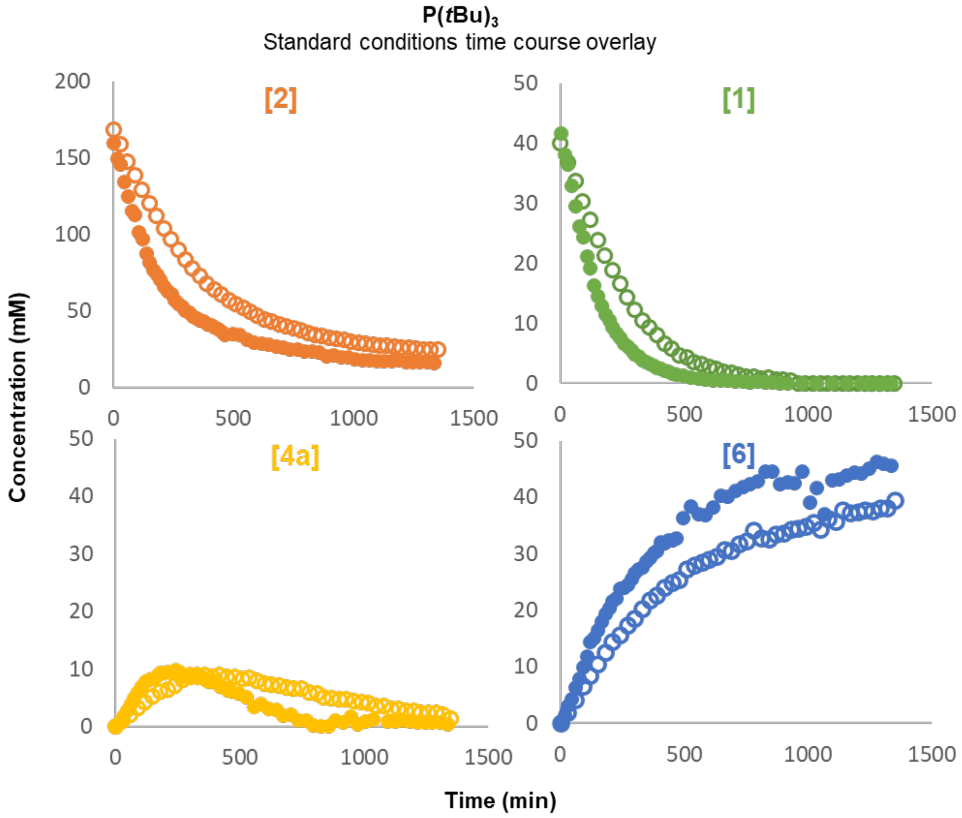

Supplementary Figure 3. Overlay of duplicate standard conditions experiments with P(*t*Bu)$_3$ as the precatalyst. Standard conditions: [**1**]$_0$ = 40 mM, [**2**]$_0$ = 180 mM, [LiHMDS]$_0$ = 200 mM, [Pd(OAc)$_2$]$_0$ = 2 mM, [P(*t*Bu)$_3$]$_0$ = 4 mM.

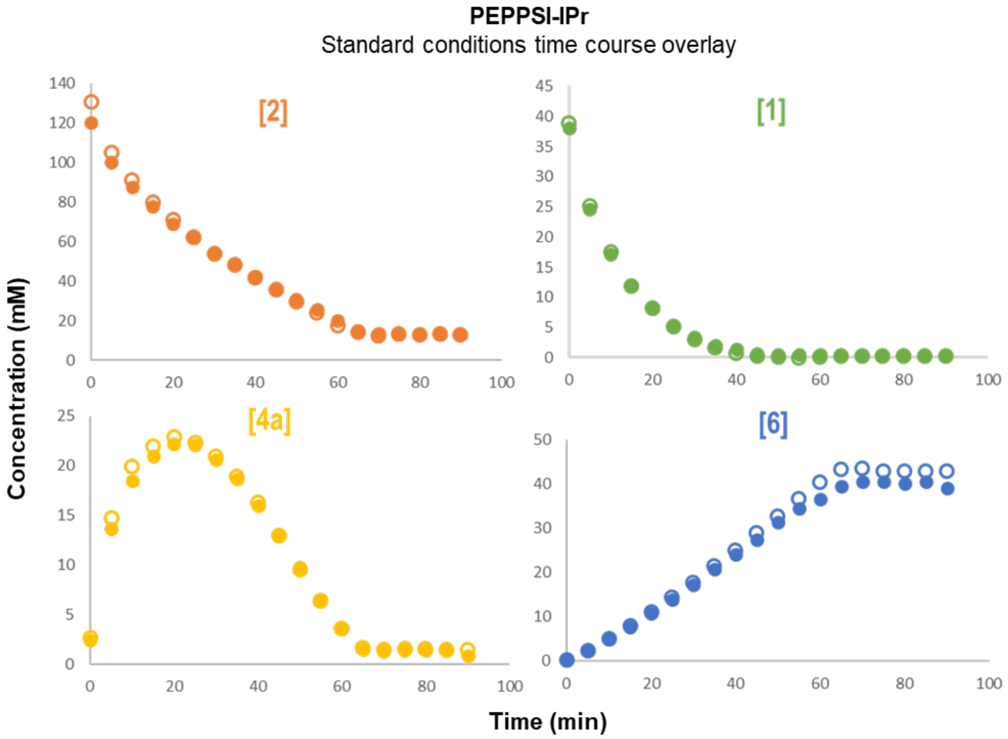

Supplementary Figure 4. Overlay of duplicate standard conditions experiments with PEPPSI-*IPr* as the precatalyst. Standard conditions: [**1**]$_0$ = 40 mM, [**2**]$_0$ = 180 mM, [LiHMDS]$_0$ = 200 mM, [PEPPSI-*IPr*]$_0$ = 2 mM.

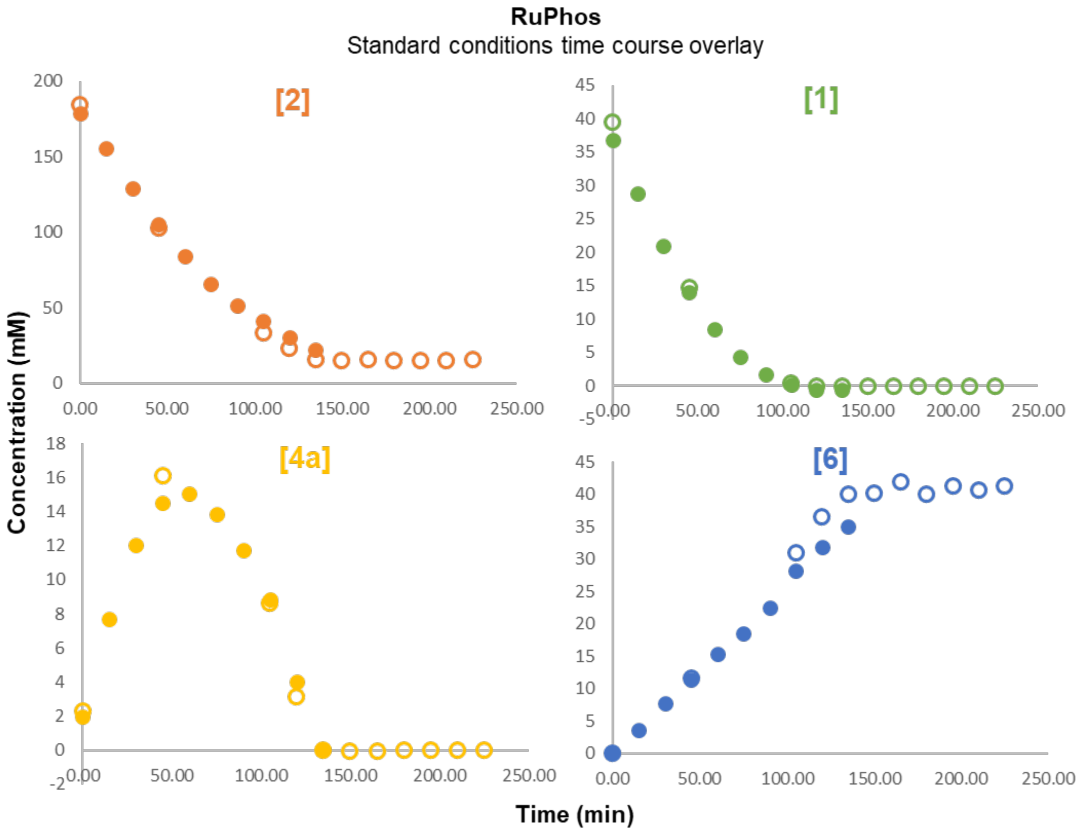

Supplementary Figure 5. Overlay of duplicate standard conditions experiments with RuPhos Pd G4 as the precatalyst. Standard conditions: $[\mathbf{1}]_0$ = 40 mM, $[\mathbf{2}]_0$ = 180 mM, $[\text{LiHMDS}]_0$ = 200 mM, $[\text{RuPhos Pd G4}]_0$ = 2 mM.

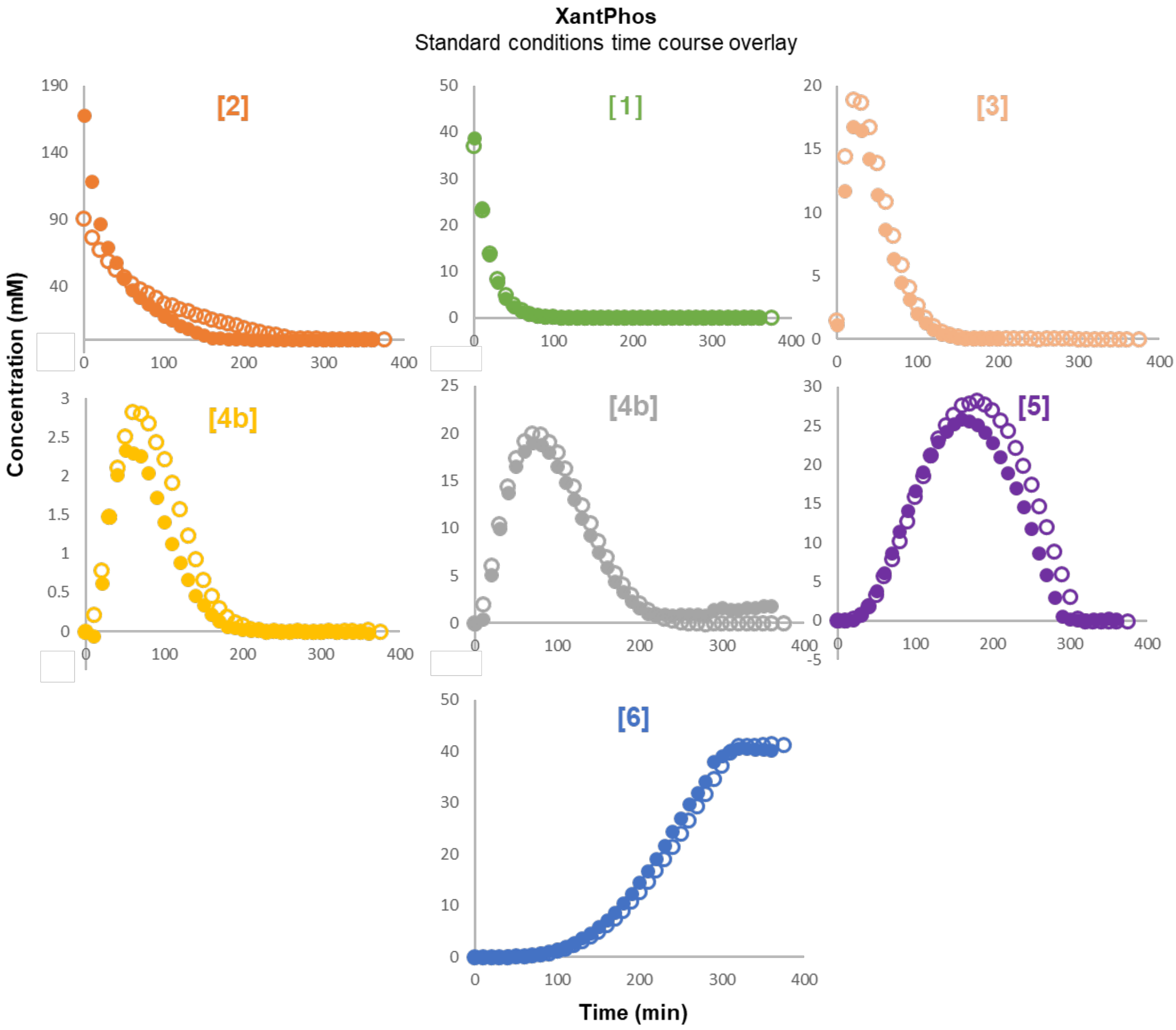

Supplementary Figure 6. Overlay of duplicate standard conditions experiments with XantPhos Pd G4 as the precatalyst. Standard conditions: [**1**]$_0$ = 40 mM, [**2**]$_0$ = 180 mM, [LiHMDS]$_0$ = 200 mM, [XantPhos Pd G4]$_0$ = 2 mM. Overlays of [**2**] are shown to demonstrate the analytical instability of **2**.

A series of different excess reactions were conducted to determine whether **1**, **2**, and LiHMDS had a positive, negative, or 0th order for each ligand system.[3,4] These results enabled us to identify the likely resting state of the catalyst. A set of different excess experiments were conducted for each precatalyst system: P(*t*Bu)$_3$, PEPPSI-*IPr*, RuPhos Pd G4, and XantPhos Pd G4.

Data processing and adjustment of 1 trends in different excess experiments: The concentration versus time profile for **1** in the different excess experiments with reduced [**1**]$_0$ have been translated by 20 mM along the y-axis in Supplementary Figure 8, Supplementary Figure 10, Supplementary Figure 12, and Supplementary Figure 13. A clear example of this translation is shown in Supplementary Figure 7. This adjustment is common in visual kinetic analyses and allows for an easier visual comparison of reaction profiles over the same catalyst turnover region.[4]

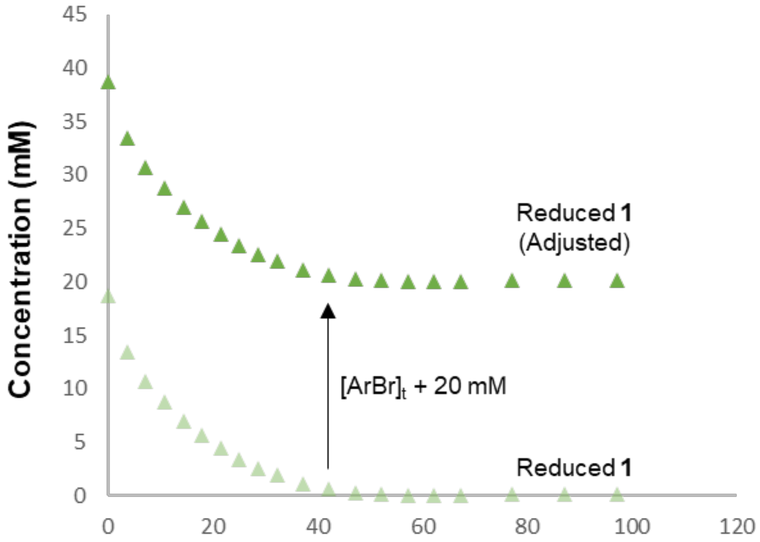

Supplementary Figure 7. Example of the adjustment made to **1** trends in the difference excess plots for reduced [**1**]$_0$ experiments.

Procedure for kinetic experiments with P(*t*Bu)$_3$: to a 16 mL vial with a stir bar inside of a glovebox, was added bis(4-methoxyphenyl)amine (**2**) (413 mg, 1.80 mmol), LiHMDS (335 mg, 2.00 mmol), 2,2',7,7'-tetrabromo-9,9'-spirobi[fluorene] (**1**) (253 mg, 0.400 mmol), THF (9.0 mL), and toluene (0.8 mL) and the resultant solution was heated to 60 °C. Three test samples were taken after 15 minutes to ensure reproducible sampling. A precatalyst solution was prepared by combining Pd(OAc)$_2$ (9.0 mg, 0.040 mmol), and 0.065 mL of 1.23 M solution of P(*t*Bu)$_3$ (16 mg, 0.080 mmol) in toluene, and diluting in 0.335 mL of toluene. The precatalyst solution sat for 30 minutes and turned from red to colourless while the test samples were taken from the reaction vial. The reaction was initiated by adding 0.2 mL of the precatalyst solution, containing Pd(OAc)$_2$ (4.5 mg, 0.020 mmol) and P(tBu)$_3$ (8.0 mg, 0.04 mmol), to the reaction vial.

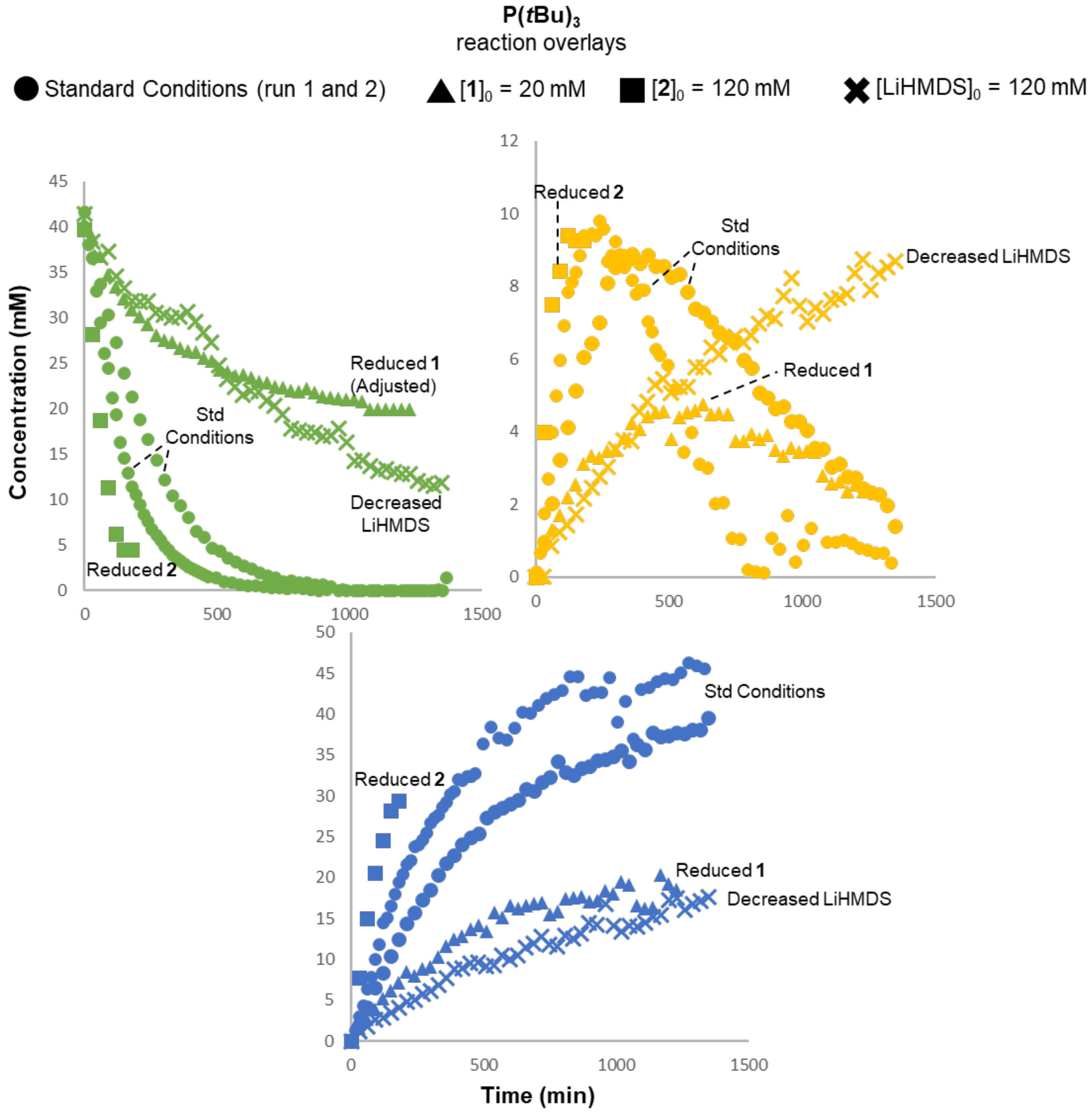

Conclusions:
positive order in **1** and LiHMDS
negative order in **2**

Supplementary Figure 8. Overlay of the reaction time course of [**1**] (top left), [**4a**] (top right), and [**6**] (bottom) for five experiments: two standard condition experiments ($[\mathbf{1}]_0$ = 40 mM, $[\mathbf{2}]_0$ = 180 mM, $[\text{LiHMDS}]_0$ = 200 mM, $[Pd(OAc)_2]_0$ = 2 mM, $[P(t\text{Bu})_3]_0$ = 4 mM), reduced aryl bromide ($[\mathbf{1}]_0$ = 20 mM), reduced amine ($[\mathbf{2}]_0$ = 120 mM), and reduced LiHMDS ($[\text{LiHMDS}]_0$ = 120 mM). See Supplementary Figure 7 for an explanation of why the 'Reduced **1**' trend in the [**1**] versus time plot starts at 40 mM.

Probing the negative order in 2 in the P(*t*Bu)$_3$ system: the system with P(*t*Bu)$_3$ exhibited a negative order dependence on **2**. An amine dosing experiment was conducted to compare the rate of reaction when **2** was linearly dosed into the reaction compared to when **2** was added to the reaction mixture as normal. This data was taken from one of the author's theses.[5] The following procedure was used in the dosing experiment:

To a 16 mL vial under an inert atmosphere was added LiHMDS (335 mg, 2.00 mmol), **1** (253 mg, 0.400 mmol), THF (9.0 mL), and toluene (0.8 mL). The stirred solution was heated at 60 °C. **2** (344 mg, 1.50 mmol) in THF (1.0 mL) was linearly dosed into the flask over 50 minutes at a rate of 0.02 mL/min. Once dosing was initiated, $Pd(OAc)_2$ (4.5 mg, 0.020 mmol) and P(*t*Bu)$_3$ (8.0 mg, 0.040 mmol) dissolved in toluene (0.2 mL) was injected via syringe to initiate the reaction and a sampling sequence was then begun. The time course reaction profile for the experiment with dosed **2** is shown in Supplementary Figure 9. Preparing a 1.5 M solution of **2** in THF and dosing 1.0 mL of the solution at a rate of 0.02 mL/min afforded the desired rate acceleration, while also avoiding coupling between **1** and LiHMDS. Introducing **2** *via* dosing resulted in a >7 fold increase of the initial reaction rate from 4.0 mM/h to 28.5 mM/h for the non-dosing and dosing experiments, respectively. This dosing experiment further validates the proposal that the order of **2** is negative.

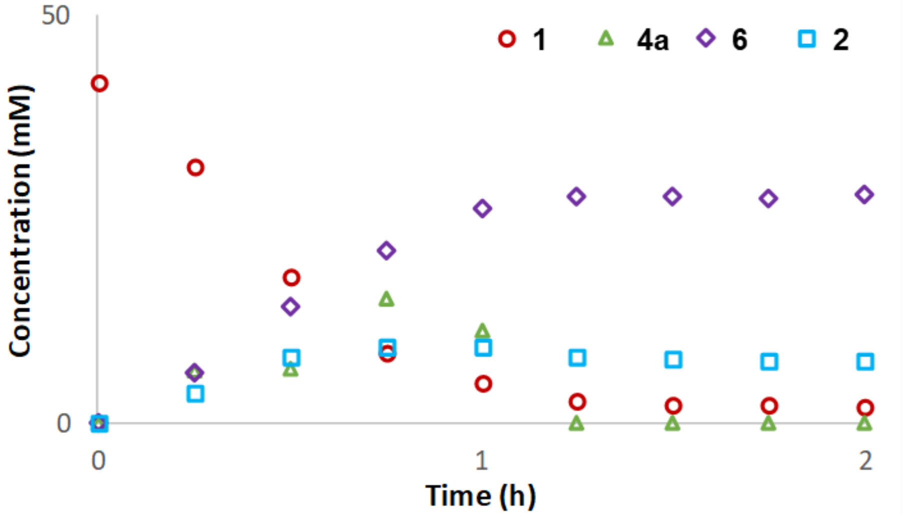

Supplementary Figure 9. Time course reaction profile for the synthesis of **6** with $Pd(OAc)_2$/P(*t*Bu)$_3$ when **2** was dosed into the reaction at a rate of 0.02 mL/min once the reaction had been initiated.

The kinetic experiments with PEPPSI-*IPr*, RuPhos, and XantPhos were run outside of the glovebox. The procedure and conditions for these experiments were modified slightly from the conditions used in the P(*t*Bu)$_3$ experiments. THF was replaced with 2-MeTHF due to the increased solubility of the aryl bromide in 2-MeTHF. Toluene was replaced with biphenyl to avoid evaporation of the internal standard in the offline HPLC samples. It should also be noted that **2** was unstable in the offline HPLC samples so the overlays of **2** were not considered in the different excess experiments.

General procedure for kinetic experiments with XantPhos, RuPhos, and PEPPSI-*IPr*: to an oven-dried 15 mL two-neck round bottom flask in the glovebox was added LiHMDS (335 mg, 2.00 mmol) and a magnetic stir bar. The flask was sealed with two rubber septa, removed from the glovebox, and put under argon on a Schlenk line. Under positive pressure, one septum was removed and 2,2',7,7'-tetrabromo-9,9'-spirobi[fluorene] (**1**) (253 mg, 0.400 mmol), bis(4-methoxyphenyl)amine (**2**) (413 mg, 1.80 mmol), and biphenyl (62 mg, 0.40 mmol) were added to the reaction flask. The flask was resealed with a septum and the system was evacuated for five minutes and backfilled with argon three times. Then, under a high flow of argon one septum was removed and the EasySampler probe was inserted into one neck of the round bottom flask. Next, 9.75 mL of 2-MeTHF was added to the reaction flask. The reaction was heated to 60 °C. After 15 minutes at 60 °C, three test samples were taken from the reaction mixture to ensure reproducible sampling. Meanwhile, a precatalyst solution was made by adding PEPPSI-*IPr* (20 mg, 0.030 mmol) to an oven-dried 1.5 mL HPLC vial. The HPLC vial with solid precatalyst was evacuated for five minutes and backfilled with argon three times before 0.75 mL of 2-MeTHF was added to the vial. The reaction was initiated by adding 0.5 mL of the precatalyst solution, containing PEPPSI-*IPr* (14 mg, 0.02 mmol), to the reaction flask.

PEPPSI-*IPr*: Reactions using PEPPSI-*IPr* as the precatalyst were run using the general procedure. Different excess results are shown in Supplementary Figure 10.

RuPhos Pd G4: Reactions using RuPhos Pd G4 as the precatalyst were run using the general procedure and RuPhos Pd G4 (25 mg, 0.030 mmol). The precatalyst solution was sonicated for 60 seconds prior to addition to the reaction flask. The reaction was initiated by adding 0.5 mL of the precatalyst solution, containing RuPhos Pd G4 (17 mg, 0.020 mmol), to the reaction flask. Different excess results are shown in Supplementary Figure 12.

XantPhos Pd G4: Reactions using XantPhos Pd G4 as the precatalyst were run using the general procedure with some modifications. The precatalyst solution was made by adding XantPhos Pd G4 (29 mg, 0.030 mmol) to an oven-dried 10 mL vial. The vial with the precatalyst was evacuated and backfilled with argon three times before 2.25 mL of 2-MeTHF was added. The solution was sonicated for 60 seconds before 1.5 mL of the precatalyst solution containing XantPhos Pd G4 (19 mg, 0.020 mmol) was added to the reaction flask. Only 8.5 mL of 2-MeTHF was initially added to the reaction flask in reactions with XantPhos Pd G4 as the precatalyst. These reactions were conducted at 40 °C. Different excess results are shown in Supplementary Figure 13.

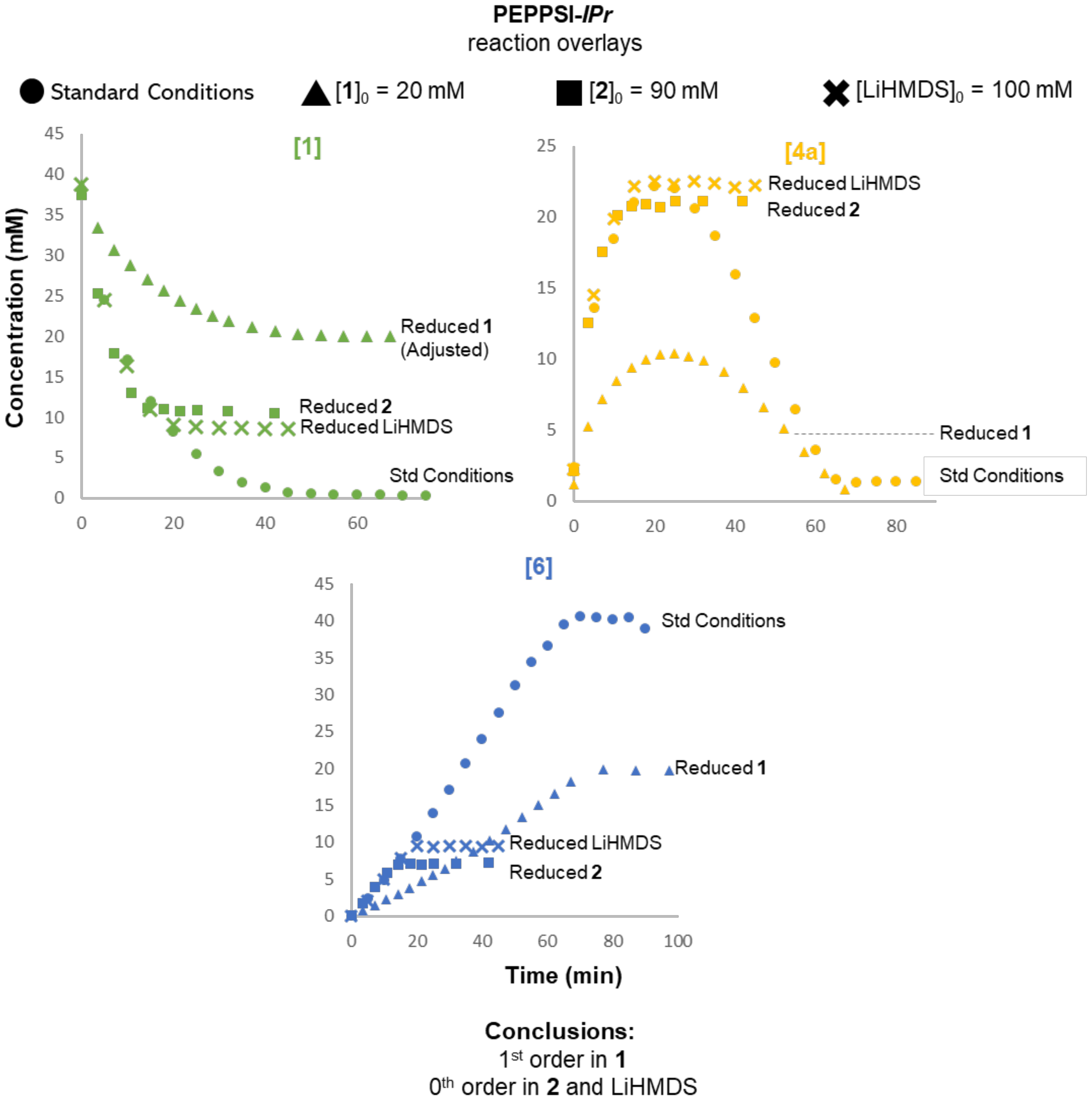

Supplementary Figure 10. Overlay of the reaction time course of [**1**] (top left), [**4a**] (top right), and [**6**] (bottom) for four experiments: standard conditions ([**1**]$_0$ = 40 mM, [**2**]$_0$ = 180 mM, [LiHMDS]$_0$ = 200 mM, [PEPPSI-*IPr*]$_0$ = 2 mM), reduced aryl bromide ([**1**]$_0$ = 20 mM), reduced amine ([**2**]$_0$ = 90 mM, [LiHMDS]$_0$ = 100 mM), and reduced LiHMDS ([LiHMDS]$_0$ = 100 mM). See Supplementary Figure 7 for an explanation of why the 'Reduced **1**' trend in the [**1**] versus time plot starts at 40 mM.

The different excess experiment with reduced **2** required a concomitant reduction in the concentration of base. When the different excess reaction was run with a high concentration of LiHMDS (200 mM, as in the standard condition experiments), there was little conversion and the reaction stalled (Supplementary Figure 11). Thus, the concentration of base was dropped to 100 mM to prevent catalyst death. LiHMDS had already been shown to have a 0th order dependence, so dropping its concentration would not affect reaction rate. Additionally,

its concentration was still greater than the amine concentration so any changes in rate or endpoint would be due to altered amine concentration.

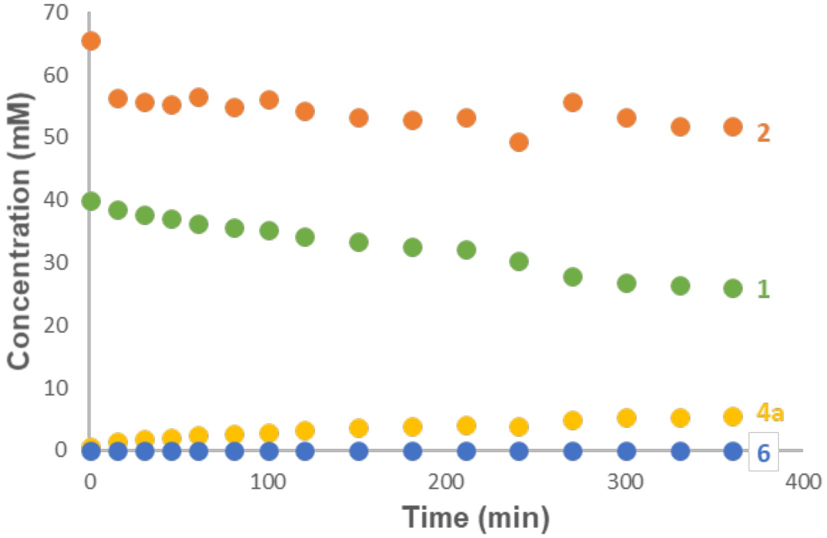

Supplementary Figure 11. Time course reaction profile of the reduced **2** different excess experiment with PEPPSI-*IPr*. $[\mathbf{2}]_0$ = 90 mM, $[LiHMDS]_0$ = 200 mM. A large excess of base deactivates the PEPPSI-*IPr* catalyst.

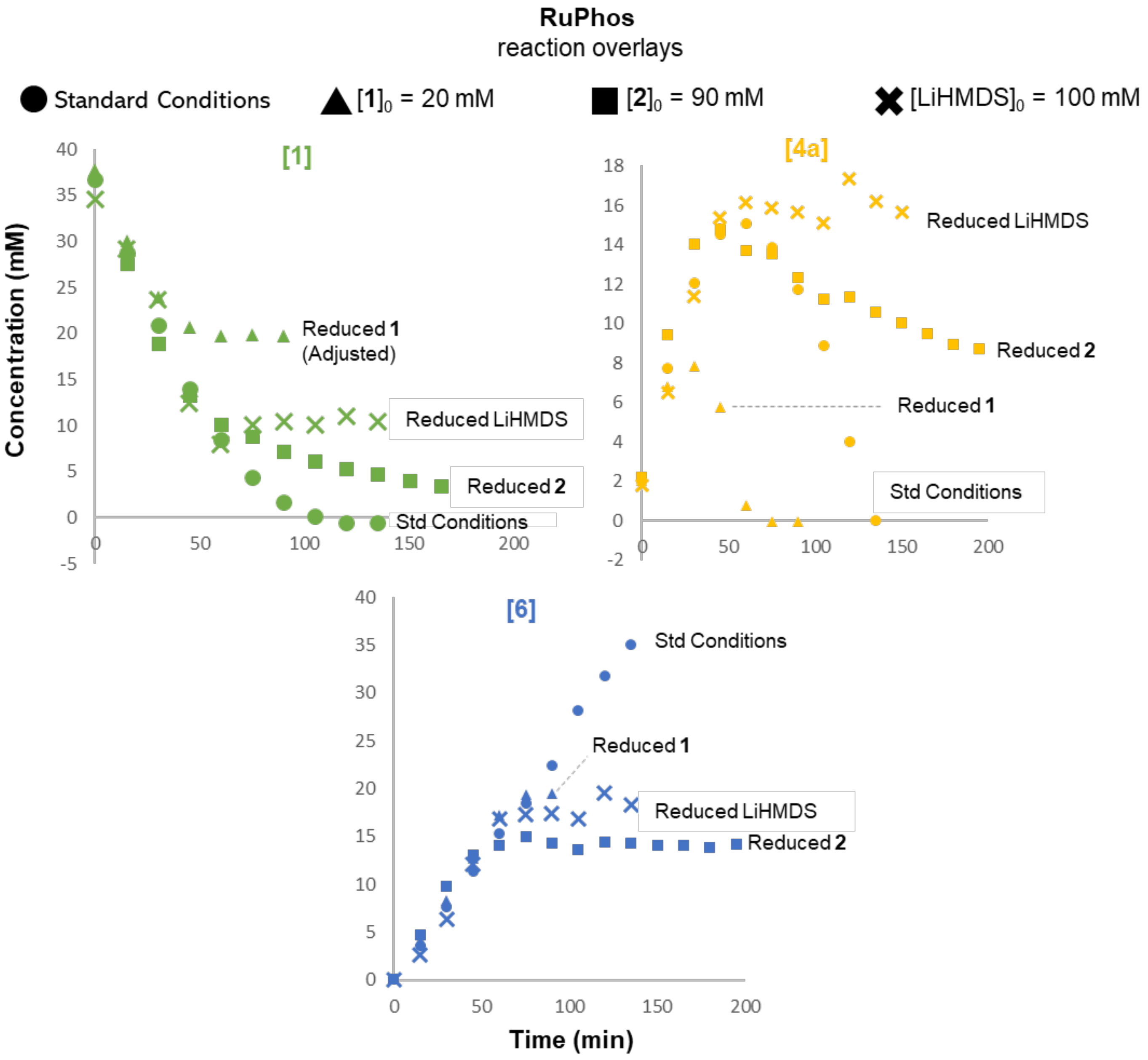

Conclusions:

0th order in **1**, **2** and LiHMDS

Supplementary Figure 12. Overlay of the reaction time course of [**1**] (top left), [**4a**] (top right), and [**6**] (bottom) for four experiments: standard conditions ([**1**]$_0$ = 40 mM, [**2**]$_0$ = 180 mM, [LiHMDS]$_0$ = 200 mM, [RuPhos Pd G4]$_0$ = 2 mM), reduced aryl bromide ([**1**]$_0$ = 20 mM), reduced amine ([**2**]$_0$ = 90 mM), and reduced LiHMDS ([LiHMDS]$_0$ = 100 mM). See Supplementary Figure 7 for an explanation of why the 'Reduced **1**' trend in the [**1**] versus time plot starts at 40 mM.

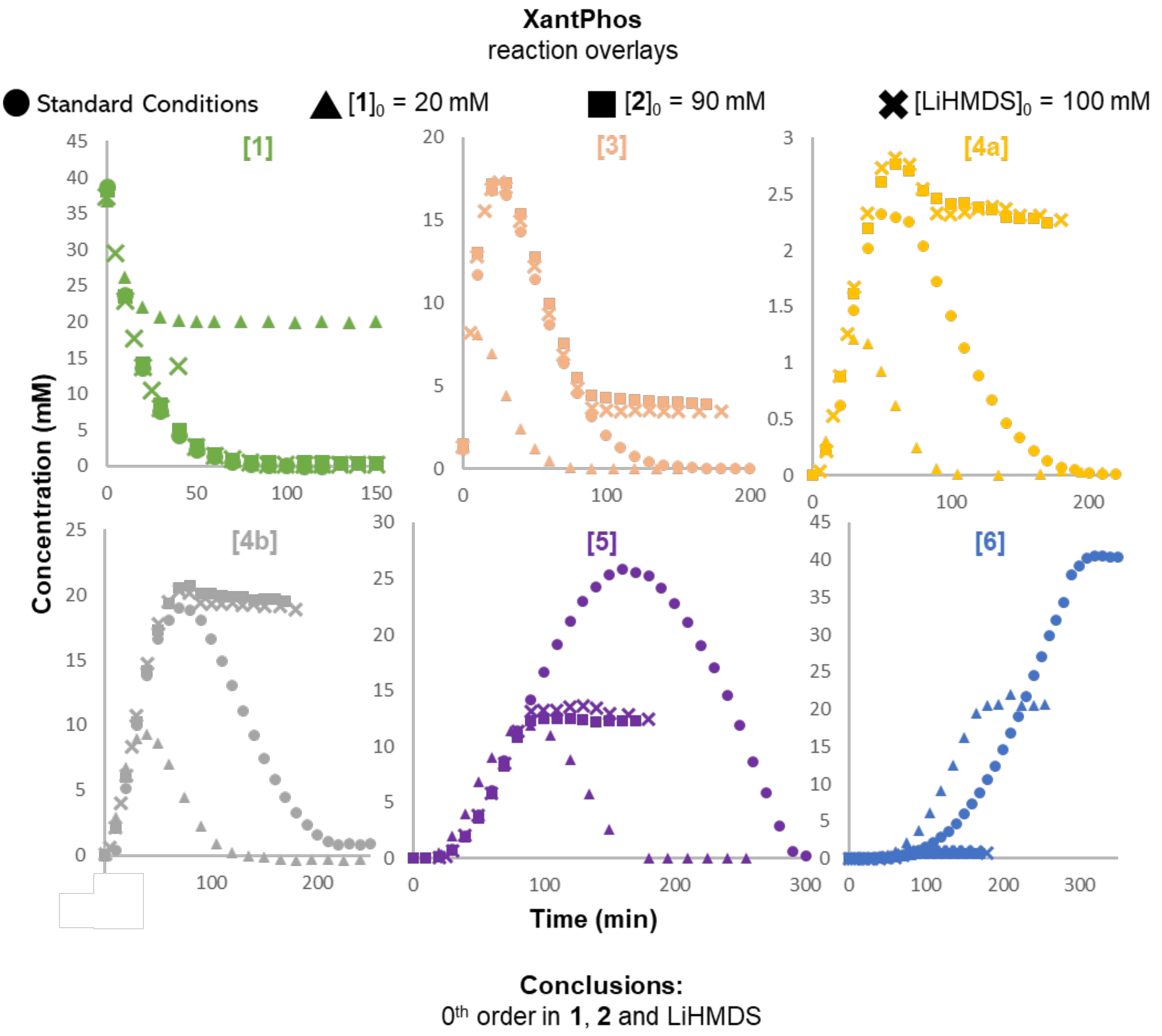

Supplementary Figure 13. Overlay of the reaction time course of [**1**] (top left), [**4a**] (top right), and [**6**] (bottom) for four experiments: standard conditions ([**1**]$_0$ = 40 mM, [**2**]$_0$ = 180 mM, [LiHMDS]$_0$ = 200 mM, [XantPhos Pd G4]$_0$ = 2 mM), reduced aryl bromide ([**1**]$_0$ = 20 mM), reduced amine ([**2**]$_0$ = 90 mM), and reduced LiHMDS ([LiHMDS]$_0$ = 100 mM). See Supplementary Figure 7 for an explanation of why the 'Reduced **1**' trend in the [**1**] versus time plot starts at 40 mM.

Conversion of peak area to concentration:

HPLC peak area (AU) was converted to concentration (mM) *via* Excel Solver. First, HPLC peak areas were tabulated and then normalized against biphenyl (internal standard). The normalized peak areas were then exported into an Excel document. An Excel worksheet was constructed that contained the data for all reactions for a ligand system (see attached Excel worksheets). To convert peak area to concentration, a peak area must be multiplied by a response factor. This response factor is a constant that is unique to each reaction species. The response factors can be solved by using the Excel Solver tool. If the response factors are calculated using Excel Solver, a series of assumptions must be made. Firstly, it was assumed that there was perfect mass balance at each time point. Secondly, it was assumed that all species that contributed to the mass balance (including starting materials, intermediates, byproducts, and products) were accounted for. An Excel worksheet was set up such that the peak areas at each time point were multiplied by a response factor to give concentration. The mass balance at each time point was calculated by summing the starting material, intermediate, and product

concentrations. An error was calculated for each time point by determining the difference between the calculated and theoretical mass balance. The total error was calculated by summing the error at each time point in the Excel sheet. The Excel Solver tool was then run and was given the objective to minimize the total error in mass balance by adjusting the response factor values. The response factors that gave concentrations resulting in the least amount of mass balance error were returned. The response factors calculated by Excel Solver for the kinetic experiments can be found in the attached Excel sheets. Different sets of response factors were calculated for each precatalyst to account for any variation in the sampling setup or HPLC.

The same data set was converted from normalized peak area to concentration using a calibration curve and then using the Solver method. A standard conditions reaction profile with the P(*t*Bu)$_3$ system was chosen for comparison. The calibration curves used are shown in Supplementary Figure 15. The concentration versus time profiles are in good agreement with each other (Supplementary Figure 14). Though the Solver method is well-accepted, we have also provided this concrete evidence to demonstrate the validity of using the Solver method in our study.

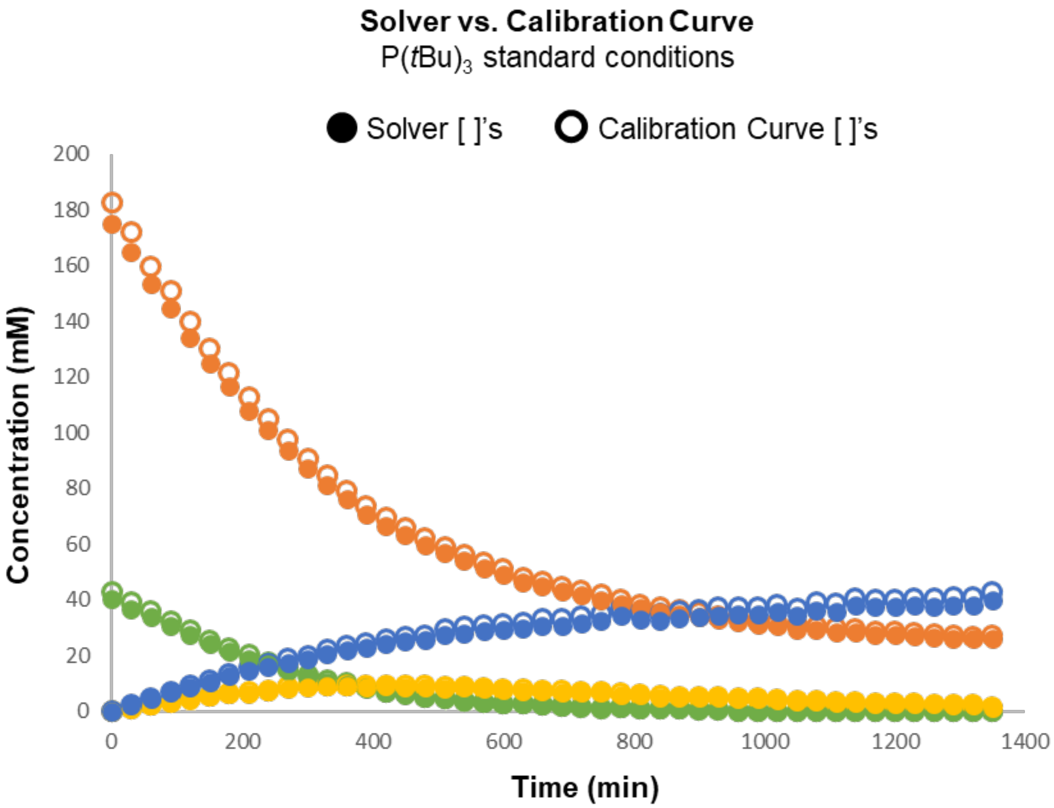

Supplementary Figure 14. Overlay of the concentration versus time profiles for the same reaction calculated *via* two different methods. Method 1: Excel Solver, solid circles. Method 2: calibration curve, open circles. The data are in good agreement with each other.

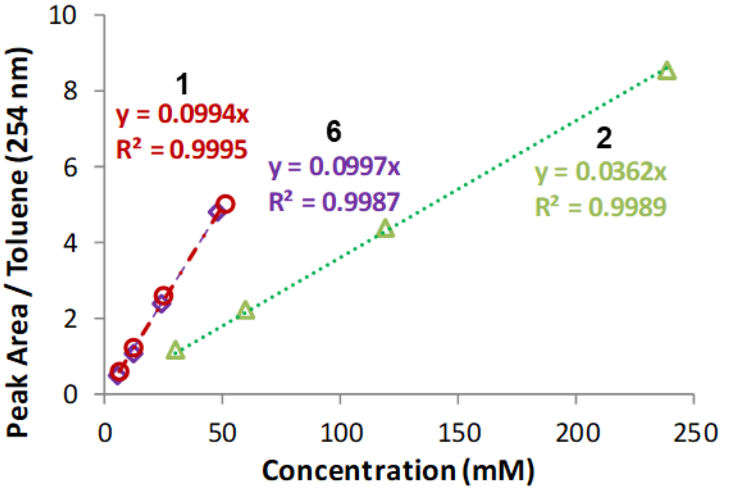

Supplementary Figure 15. Calibration curves used to calculate the concentration vs time profiles shown in Supplementary Figure 14.

Assignment of intermediates in the synthesis of 6 and 17

The identity of intermediates **3**, **4b**, **5**, **14a**, **14b**, **15a**, **15b**, **16a**, and **16b** was determined by low-resolution mass spectrometry (LRMS). The intermediates **4a**, **8,** and **11** were identified by LRMS and were subsequently isolated and fully characterized to unambiguously confirm their identity. Because the identity of intermediates in two systems was unambiguously confirmed (and aligned with the conclusions drawn from the LRMS data), we are confident in the assignment of intermediates in the other two systems based on LRMS data.

Supplementary Figure 16a shows a screen capture of an HPLC chromatogram with **3**, **4a**, **4b**, **5**, and **6**. Below the HPLC chromatogram there is a screen capture of the mass chromatogram, showing the signal intensity of mass active species versus retention time. The masses of the four intermediates and product (M^+) were extracted from the mass chromatogram and the resulting extracted ion chromatograms (EIC) are shown in Supplementary Figure 16b. This shows that for each of the extracted masses, there was one time at which a chemical species with that mass was detected. For example, only one compound with a mass between 778.5-779.5 m/z was present in the sample. This mass corresponds to the mass of the monocoupled intermediate **3** and the peak at 7.996 min was therefore identified as **3**. Similar reasoning was used to identify **4b** and **5** for this system. The LRMS for **3** (Supplementary Figure 23), **4b** (Supplementary Figure 26), and **5** (Supplementary Figure 27) are shown in the spectra section of the SI.

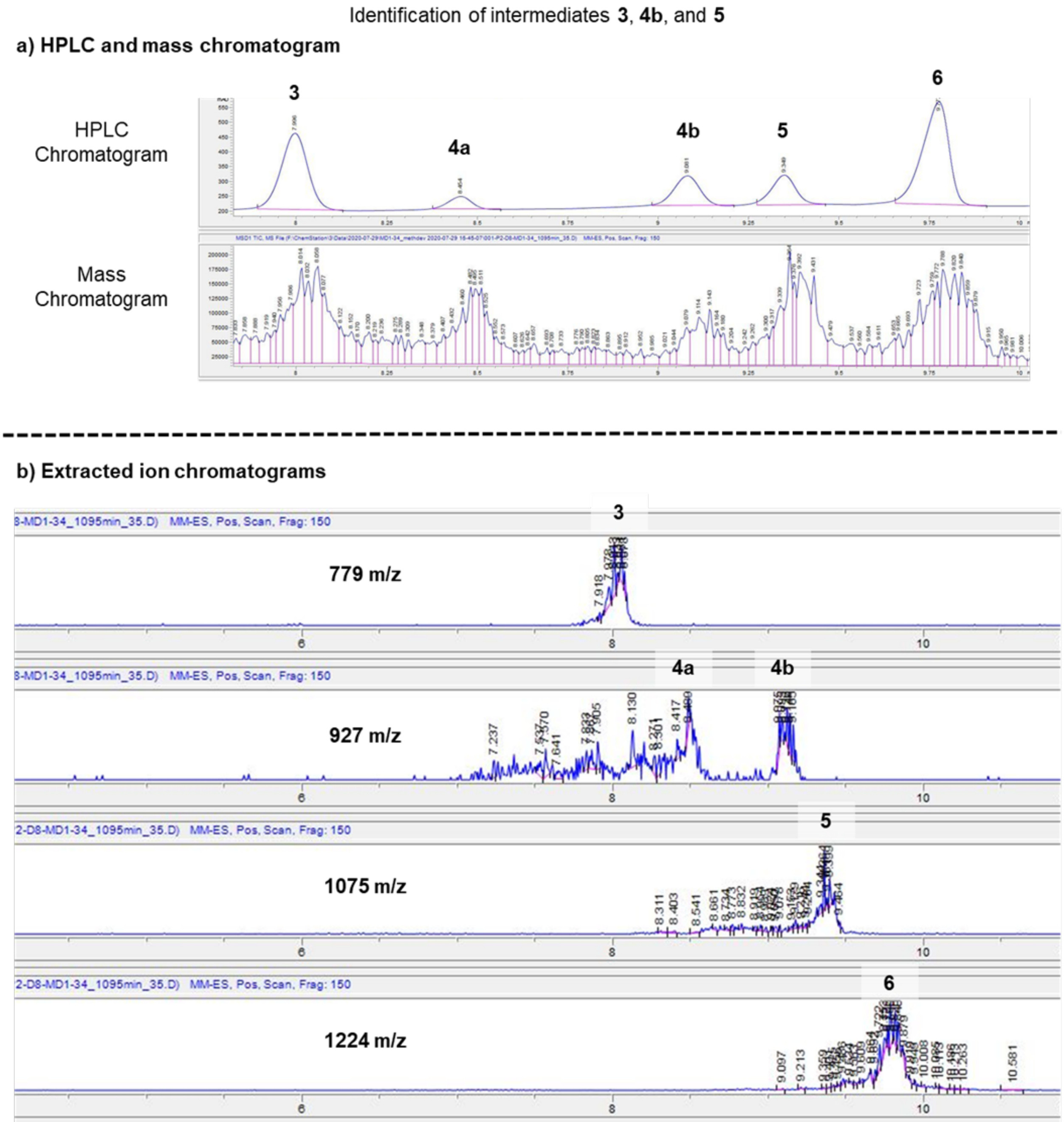

Supplementary Figure 16a. HPLC chromatogram (top) showing the peaks for compounds **3** – **6** and the mass chromatogram (bottom) showing the intensity of mass active species according to retention time. Each HPLC peak has a corresponding LRMS hit. b, the EICs corresponding to the masses of the mono, di, tri, and tetracoupled intermediates/product.

Supplementary Figure 17a shows the HPLC chromatograms from two timepoints during the synthesis of **17**. The peaks corresponding to intermediates **14a**, **14b**, **15a**, **15b**, **16a**, and **16b** are highlighted. Based on the EICs (Supplementary Figure 17b) and LRMS data for each peak, it was determined which peaks represented mono, di, and tricoupled intermediates. Since we were unable to separate and isolate the intermediates, we could not determine which regioisomers of the mono, di, and tricoupled intermediates were formed. The time course reaction profile in figure 10b shows **14a** as the major monocoupled intermediate. We could not confirm this was the major regioisomer, but assumed that the first amine equivalent would add to the more electron deficient lower aryl ring system (as oxidative addition is easier in electron deficient systems). The two di and tri regioisomers were formed in near-equal amounts and were arbitrarily assigned as either **15a**/**15b** and **16a**/**16b**. The mass spectra for **14a** (Supplementary Figure 38), **14b** (Supplementary Figure 39), **15a** (Supplementary

Figure 40), **15b** (Supplementary Figure 41), **16a** (Supplementary Figure 42), and **16b** (Supplementary Figure 43) are included in the spectra section of the SI.

Identification of intermediates **14a**, **14b**, **15a**, **15b**, **16a**, and **16b**

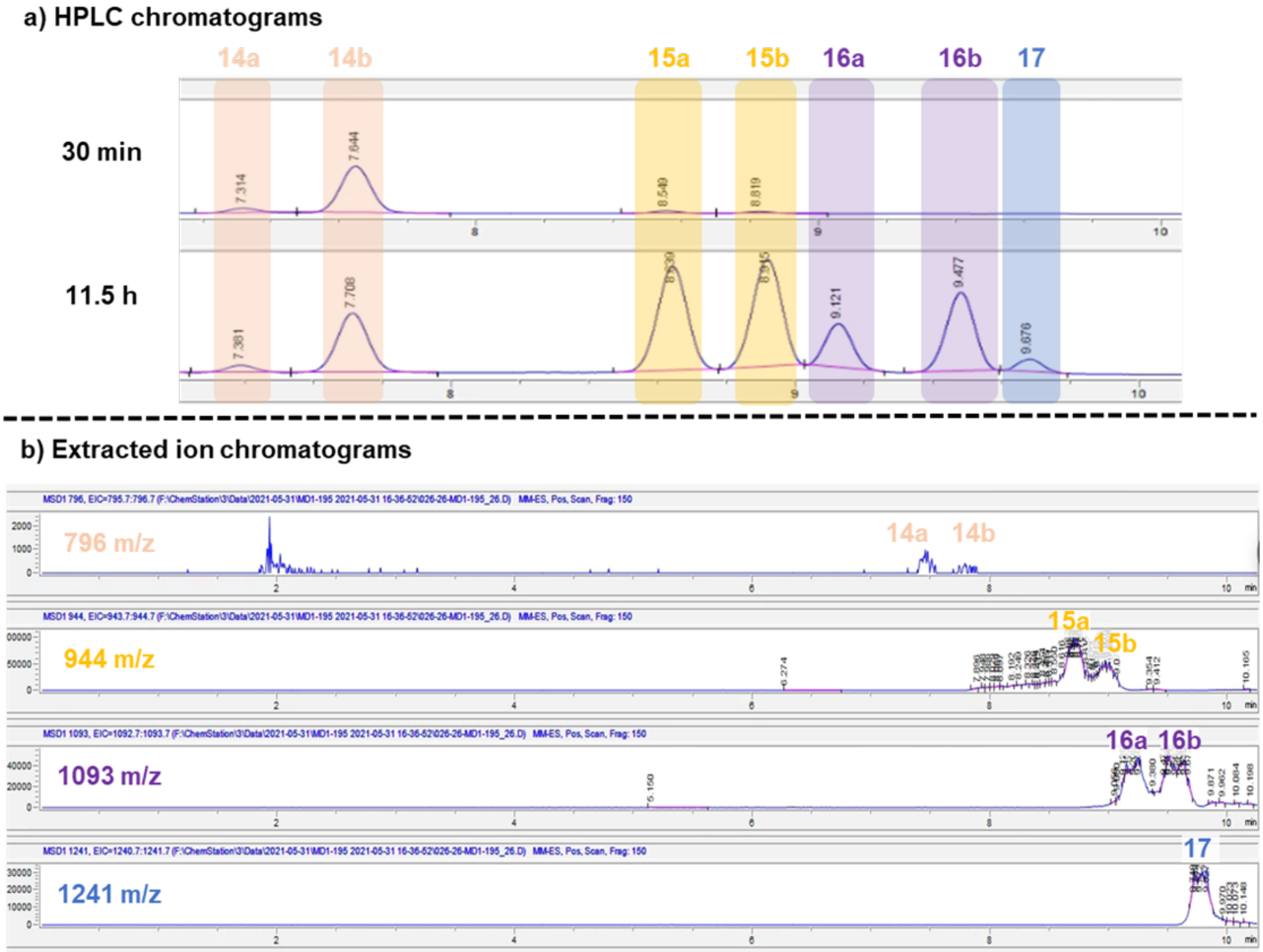

Supplementary Figure 17a. HPLC chromatograms at two timepoints showing the peaks and retention times for 14a – 17. b, the EICs corresponding to the masses of the mono, di, tri, and tetracoupled intermediates/product.

COPASI modeling

A total of three basic models were required to recapitulate the reaction behaviour for the various ligand systems. In each case, a minimal model was constructed, containing reaction components, catalyst intermediates and products as detailed below. Some reaction elements (such as the transient concentration of amine, and base) were omitted from the model as our VTNA experiments revealed that these species were not kinetically relevant. Each system was created in COPASI, using the parameter estimation function to identify optimal values for the indicated rate constants (genetic algorithm with population size of 20, number of generations 20000) In each case, optimization was executed until the resultant error converged and showed no further improvement.

Minimal model #1 - Ring Walking

A reaction set comprised of four kinetically relevant steps was required to recapitulate the time course data from the RuPhos, and PEPPSI-*IPr* systems.

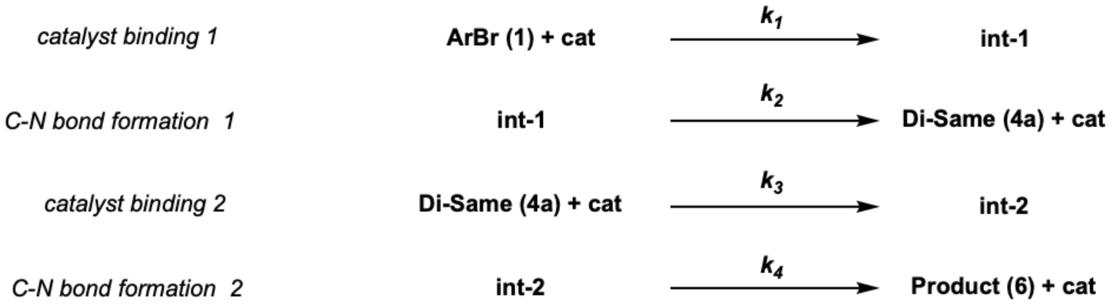

Optimized values for listed rate constants are:

	k_1 *(mL / mmol * s)*	k_2 *(1/s)*	k_3 *(mL / mmol * s)*	k_4 *(1/s)*
RuPhos	0.0257	0.4157	0.02511	390.295
PEPPSI	80.1	1.801	30.33	0.3537

<u>Minimal model #2 - Ring Walking with Diffusion Control</u>

Similar to model #1, we use a minimal set of kinetically relevant steps, with the addition of a direct path from starting aryl bromide **1**, to give product **6**. This model is most representative of the $P(tBu)_3$ ligand set, but we did apply the same to both RuPhos and PEPPSI-*IPr*. The addition of this added diffusion controlled reaction does not significantly improve the fit of our model and thus it becomes inconclusive to judge if diffusion control is operative with these ligands. Suffice to say our observed kinetic data can be recapitulated without adding diffusion controlled coupling to PEPPSI-*IPr* and RuPhos.

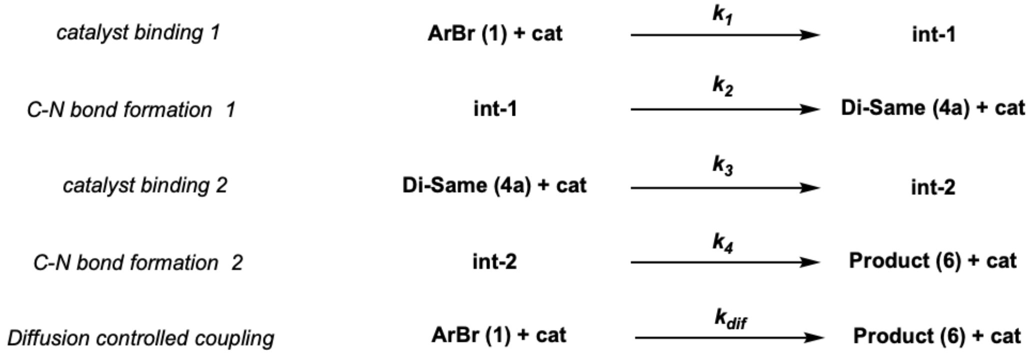

Optimized values for listed rate constants are:

	k_1 *(mL / mmol * s)*	k_2 *(1/s)*	k_3 *(mL / mmol * s)*	k_4 *(1/s)*	k_{dif} *(mL / mmol * s)*
$P(tBu)_3$ (no Diffusion)	8.292×10^{-4}	395.0	1.316×10^{-3}	399.6	—----
$P(tBu)_3$ (with Diffusion)	5.129×10^{-4}	0.0318	4.80×10^{-4}	425.2	7.724×10^{-4}

<u>Minimal model #3 - Stepwise coupling</u>

Our final model is an evolution of the previous two, now incorporating sequential C-Br reaction and C-N bond formation. This model also entertains the possibility for multiple regioisomers through reaction of the monofunctionalized product.

catalyst binding 1	**ArBr (1) + cat** $\xrightarrow{k_1}$ **int-1**	
C-N bond formation 1	**int-1** $\xrightarrow{k_2}$ **Mono (3) + cat**	
catalyst binding 2	**Mono (3) + cat** $\xrightarrow{k_3}$ **int-2**	
C-N bond formation 2	**int-2** $\xrightarrow{k_4}$ **Di-Same (4a) + cat**	
catalyst binding 3	**Mono (3) + cat** $\xrightarrow{k_5}$ **int-3**	
C-N bond formation 3	**int-3** $\xrightarrow{k_6}$ **Di-Opp (4b) + cat**	
catalyst binding 4	**Di-Same (4a) + cat** $\xrightarrow{k_7}$ **int-4**	
C-N bond formation 4	**int-4** $\xrightarrow{k_8}$ **Tri (5) + cat**	
catalyst binding 5	**Di-Opp (4b) + cat** $\xrightarrow{k_9}$ **int-5**	
C-N bond formation 5	**int-5** $\xrightarrow{k_{10}}$ **Tri (5) + cat**	
catalyst binding 6	**Tri (5) + cat** $\xrightarrow{k_{11}}$ **int-6**	
C-N bond formation 6	**int-6** $\xrightarrow{k_{12}}$ **Product (6) + cat**	

Optimized values for listed rate constants in the XantPhos system:

k_1 (mL / mmol * s)	k_2 (1/s)	k_3 (mL / mmol * s)	k_4 (1/s)	k_5 (mL / mmol * s)	k_6 (1/s)
1.989	0.744	0.172	1.056	0.177	1.420
k_7 (mL / mmol * s)	k_8 (1/s)	k_9 (mL / mmol * s)	k_{10} (1/s)	k_{11} (mL / mmol * s)	k_{12} (1/s)
0.517	0.325	2.778	0.159	0.068	0.161

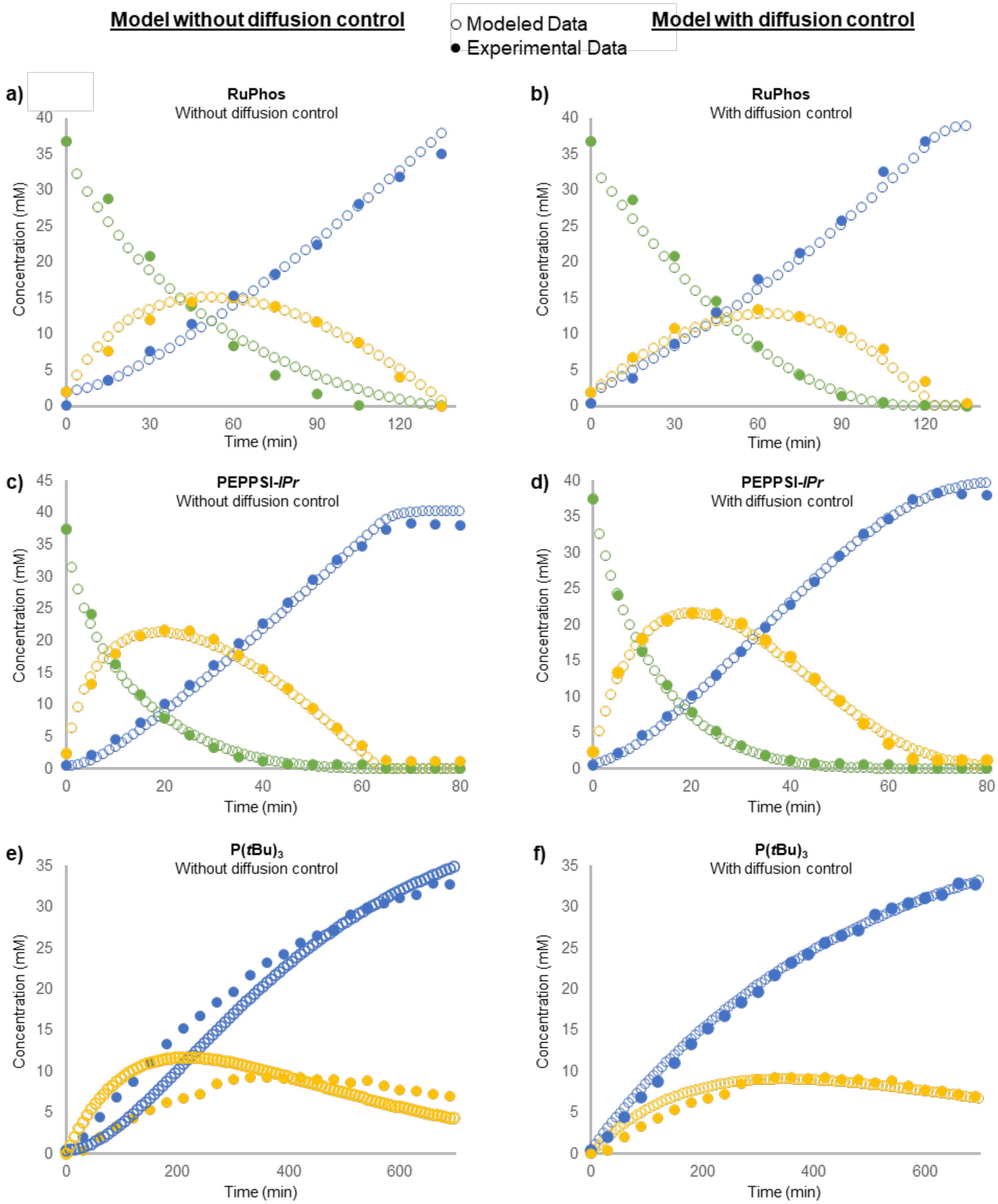

Supplementary Figure 18a. The COPASI modelled data without diffusion-controlled coupling overlayed with the acquired experimental data for RuPhos Pd G4. b, The COPASI modelled data with diffusion-controlled coupling overlayed with the acquired experimental data for RuPhos Pd G4. c, The COPASI modelled data without diffusion-controlled coupling overlayed with the acquired experimental data for PEPPSI-*IPr*. d, The COPASI modelled data with diffusion-controlled coupling overlayed with the acquired experimental data for PEPPSI-*IPr*. e, The COPASI modelled data without diffusion-controlled coupling overlayed with the acquired experimental data for $Pd(OAc)_2$/P(*t*Bu)$_3$. f, The COPASI modelled data with diffusion-controlled coupling overlayed with the acquired experimental data for $Pd(OAc)_2$/P(*t*Bu)$_3$.

Caveats to COPASI modelling: The COPASI modelling conducted on these systems does not prove or disprove the presence of each individual step included in the model. Given the results of our mechanistic study, many of these steps (such as amine coordination, ring walking, etc.) are not kinetically relevant under the reaction conditions. The model system was chosen based on the currently established mechanism for Buchwald Hartwig aminations combined with our experimental results. Consequently, the k values reported serve as a means to judge the kinetic relevance of each modeled step, rather than a quantitative measure of k values. Instead, the true utility of this analysis lies in the ability to highlight that the simplified mechanism lacks the ability to account for the observed behavior with P(*t*Bu)$_3$. In contrast, the addition of diffusion control to this model system accounts for the observed kinetic behavior very well, thus supporting its presence.

Alternate substrates

General procedure for time course reaction profiles: To an oven-dried 15 mL two-neck round bottom flask in the glovebox was added LiHMDS (167 mg, 1.00 mmol) and a magnetic stir bar. The flask was sealed with two rubber septa, removed from the glovebox, and put under argon on a Schlenk line. Under positive pressure, one septum was removed and an aryl bromide (0.40 mmol), bis(4-methoxyphenyl)amine (**2**) (206 mg, 0.900 mmol), and biphenyl (62 mg, 0.40 mmol) were added to the reaction flask. The flask was resealed with a septum and the system was evacuated for five minutes and backfilled with argon three times. Then, under a high flow of argon one septum was removed and the EasySampler probe was inserted into one neck of the round bottom flask. Next, 9.5 mL of 2-MeTHF was added to the reaction flask. The reaction was heated to 60 °C. After 15 minutes at 60 °C, three test samples were taken from the reaction mixture to ensure reproducible sampling. Meanwhile, a precatalyst solution was made by adding the precatalyst to an oven-dried vial. The vial with solid precatalyst was evacuated for five minutes and backfilled with argon three times before 0.75 mL of 2-MeTHF was added to the vial. Finally, 0.5 mL of the precatalyst solution was added to the reaction flask and the sampling sequence was immediately started.

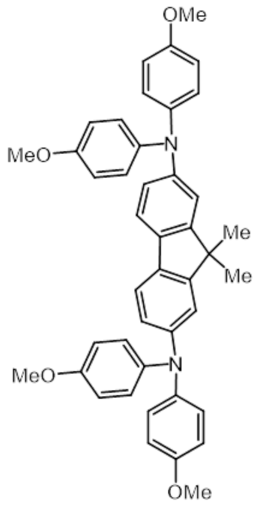

N2,N2,N7,N7-tetrakis(4-methoxyphenyl)-9,9-dimethyl-9H-fluorene-2,7-diamine (**9**)

PEPPSI-*IPr*: the time course reaction profile for the synthesis of **9** with PEPPSI-*IPr* (14 mg, 0.020 mmol) was generated according to the general procedure using 2,7-dibromo-9,9-dimethyl-9H-fluorene (**7**) (141 mg, 0.400 mmol). The time course reaction profile is shown in figure 9a.

XantPhos Pd G4: the time course reaction profile for the synthesis of **9** with XantPhos Pd G4 was generated according to the general procedure using 2,7-dibromo-9,9-dimethyl-9H-fluorene (**7**) (141 mg, 0.400 mmol). The following modifications were also made: 8.5 mL of 2-MeTHF was added to the reaction flask initially and the reaction was conducted at 40 °C. The precatalyst solution was prepared by adding XantPhos Pd G4 (29 mg, 0.030 mmol) to an oven-dried 10 mL vial. The vial was evacuated for five minutes and backfilled with argon three times before 2.25 mL of 2-MeTHF was added to the vial. The solution was sonicated for 60 seconds before 1.5 mL of the precatalyst solution containing XantPhos Pd G4 (19 mg, 0.020 mmol) was added to the reaction flask. The time course reaction profile of the synthesis of **9** with XantPhos Pd G4 is shown in figure 9b.

See synthetic procedures section for characterization data of the intermediate **8** (page S) and the product **9** (page S).

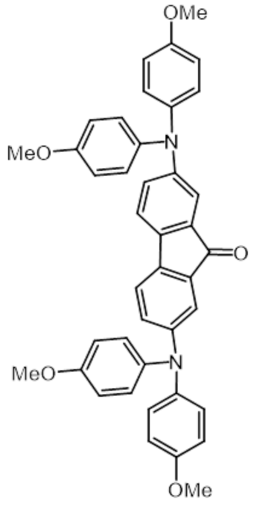

2,7-bis(bis(4-methoxyphenyl)amino)-9H-fluoren-9-one (**12**)

PEPPSI-*IPr*: the time course reaction profile for the synthesis of **12** with PEPPSI-*IPr* (14 mg, 0.020 mmol) was generated according to the general procedure using 2,7-dibromo-9H-fluoren-9-one (**10**) (135 mg, 0.400 mmol) and was conducted at 40 °C. The time course reaction profile is shown in figure 9c.

XantPhos Pd G4: the time course reaction profile for the synthesis of **12** with XantPhos Pd G4 was generated according to the general procedure using 2,7-dibromo-9H-fluoren-9-one (**10**) (135 mg, 0.400 mmol). The following modifications were made: 8.5 mL of 2-MeTHF was added to the reaction flask initially and the reaction was conducted at 25 °C. The precatalyst solution was prepared by adding XantPhos Pd G4 (43 mg, 0.045 mmol) to an oven-dried 10 mL vial. The vial was evacuated for five minutes and backfilled with argon three times before 2.25 mL of 2-MeTHF was added to the vial. The solution was sonicated for 60 seconds before 1.5 mL of the precatalyst solution containing XantPhos Pd G4 (29 mg, 0.030 mmol) was added to the reaction flask. The time course reaction profile of the synthesis of **12** with XantPhos Pd G4 is shown in figure 9d.

See synthetic procedures section for characterization data of the intermediate **11** (page S) and the product **12** (page S).

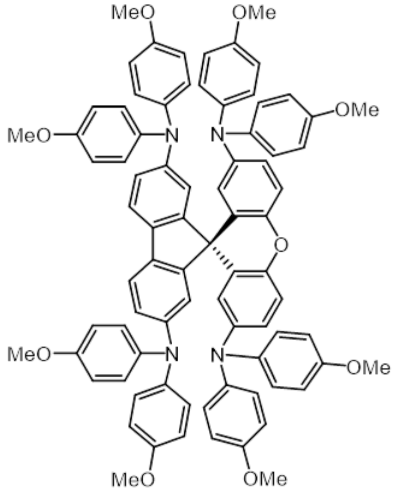

N2,N2,N2',N2',N7,N7,N7',N7'-octakis(4-methoxyphenyl)spiro[fluorene-9,9'-xanthene]-2,2',7,7'-tetraamine (**17**)

PEPPSI-*IPr*: the time course reaction profile for the synthesis of **17** with PEPPSI-*IPr* (16 mg, 0.024 mmol) was generated according to the general procedure using 2,2',7,7'-tetrabromospiro[fluorene-9,9'-xanthene] (**13**) (259 mg, 0.400 mmol), LiHMDS (335 mg, 2.00 mmol), and bis(4-methoxyphenyl)amine (413 mg, 1.80 mmol). The time course reaction profile is shown in figure 10a.

XantPhos Pd G4: the time course reaction profile for the synthesis of **17** with XantPhos Pd G4 was generated according to the general procedure using 2,2',7,7'-tetrabromospiro[fluorene-9,9'-xanthene] (**13**) (259 mg, 0.400 mmol). The following modifications were made: 8.5 mL of 2-MeTHF was added to the reaction flask initially and the reaction was conducted at 40 °C. The precatalyst solution was prepared by adding XantPhos Pd G4 (43 mg, 0.045 mmol) to an oven-dried 10 mL vial. The vial was evacuated for five minutes and backfilled with argon three times before 2.25 mL of 2-MeTHF was added to the vial. The solution was sonicated for 60 seconds before 1.5 mL of the precatalyst solution containing XantPhos Pd G4 (29 mg, 0.030 mmol) was added to the reaction flask. The time course reaction profile of the synthesis of **17** with XantPhos Pd G4 is shown in figure 10b.

See synthetic procedures section (page S) for characterization data of **17**.

The response factors used to convert peak area (AU) to concentration (M) can be found in the attached Excel workbook containing data for the alternate substrates and were calculated according to the Excel Solver method described above (page S).

Relative rates of 1st vs 2nd C-N coupling of substrate 7: The observed selectivity of the dibrominated substrates **7** and **10** could, theoretically, arise from a difference in relative rates of the C-N cross couplings rather than ring-walking or diffusion-controlled coupling (Figure 9a and c). If the second C-N cross coupling was extremely fast relative to the first, then there would be no build up or observable concentration of the monoaminated intermediates (**8** and **11** respectively). To disprove this possibility, we isolated the monoaminated intermediate **8** and gathered time course data of the amination of **8** using PEPPSI-*I*Pr as the precatalyst. These conditions approximate the 2nd C-N cross coupling and can be used to estimate the rate of the 2nd amination.

We first repeated the time course shown in the manuscript (Figure 9a) as our 2-MeTHF was contaminated with oxygen and we needed to ensure reproducibility. Incomplete conversion (approximately 95%) of **7** was observed but the rate of the reaction was comparable to the original data (Supplementary Figure 19A). Having repeated previous results to account for any starting material or reagent degradation, we then gathered time course data of the reaction using **8** as the starting material (Supplementary Figure 19B). The time course data

show that the conversion of **8** into **9** is done in 20 minutes (Supplementary Figure 19B). Supplementary Figure 20 shows the overlay of the reaction using **7** as the aryl bromide starting material versus **8** as the starting material.

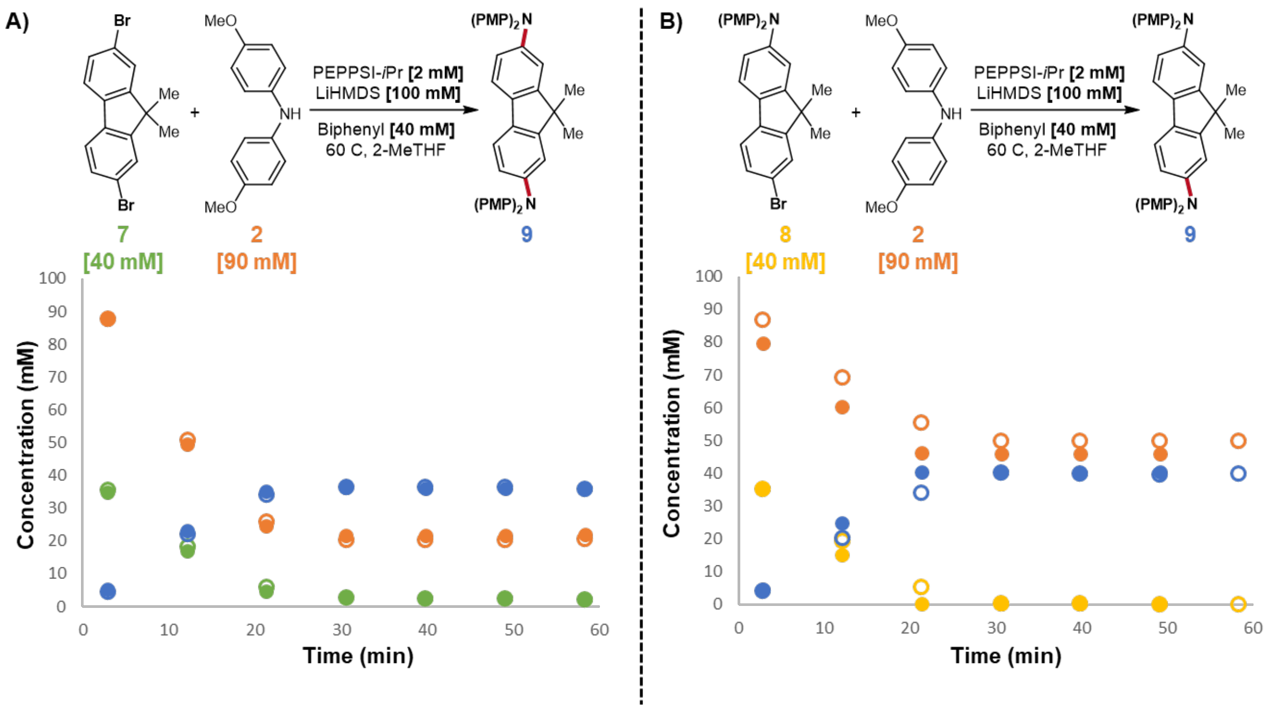

Supplementary Figure 19 A) Time course data of the amination of **7** with PEPPSI-*i*Pr. This data is a repeat of Figure 9a in the manuscript meant to account for variations arising from degradation of reagents. There are two runs of the same reaction represented by open and closed circles. B) Time course data of the amination of **8** with PEPPSI-*i*Pr. There are two runs of the same reaction represented by open and closed circles.

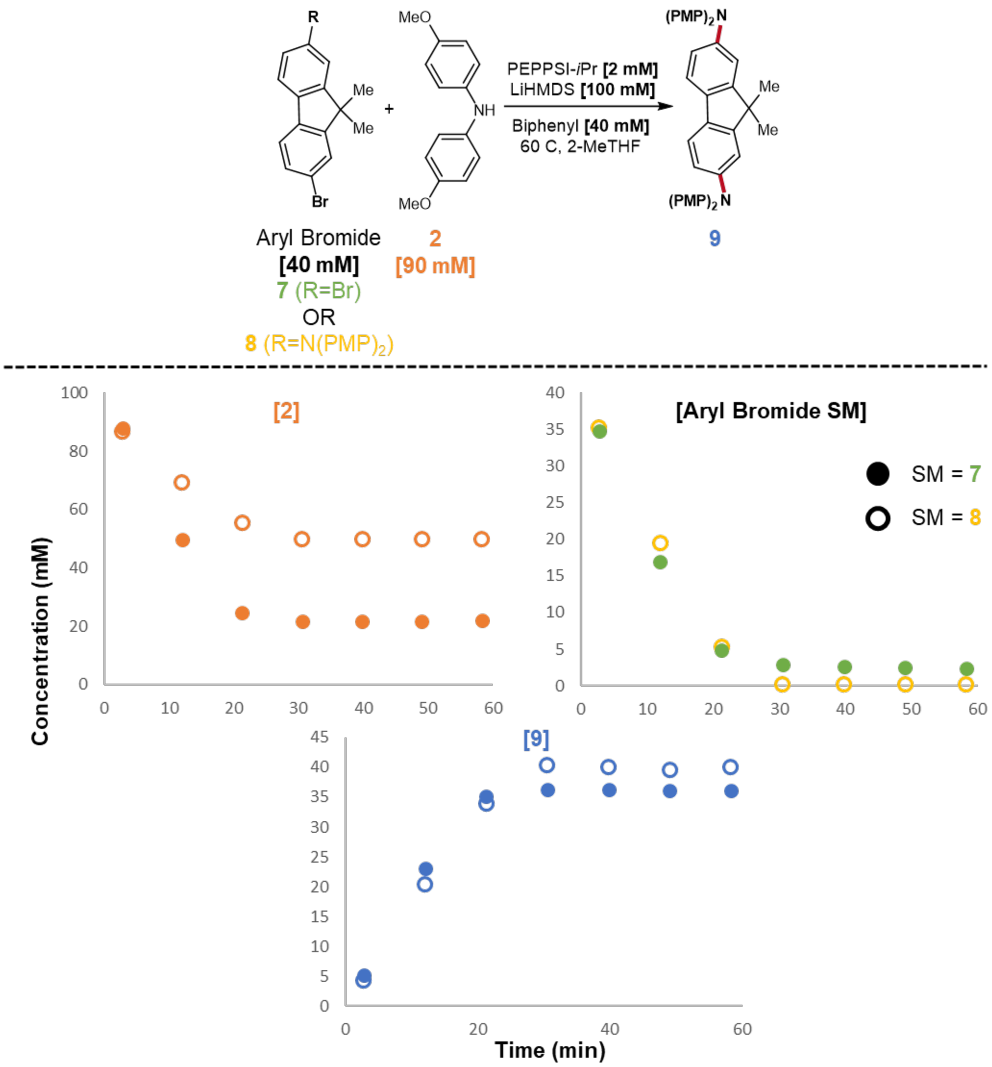

Supplementary Figure 20. Overlays of the amination of **7** and the amination of **8** with PEPPSI-IPr as the precatalyst.

The rates of the reaction with **7** and **8** are similar. Given this, we can conclude that the absence of **8** in Figure 9a is not due to a difference in relative rates.

This relative rates comparison was repeated using RuPhos Pd G4 as the precatalyst. As expected, no monocoupled intermediate was observed in the time course data with **7** as the substrate (Supplementary Figure 21). To ensure this absence was not due to a fast rate of reaction of the intermediate, time course data with **8** was collected. The conversion of **8** into product was slightly faster than the reaction with **7** as the starting material (Supplementary Figure 21). However, the magnitude of difference in rate of aryl bromide consumption is not enough to account for a complete absence of **8** in the reaction with **7**. Thus, ring walking remains the best explanation for the observed reactivity and intermediate distribution.

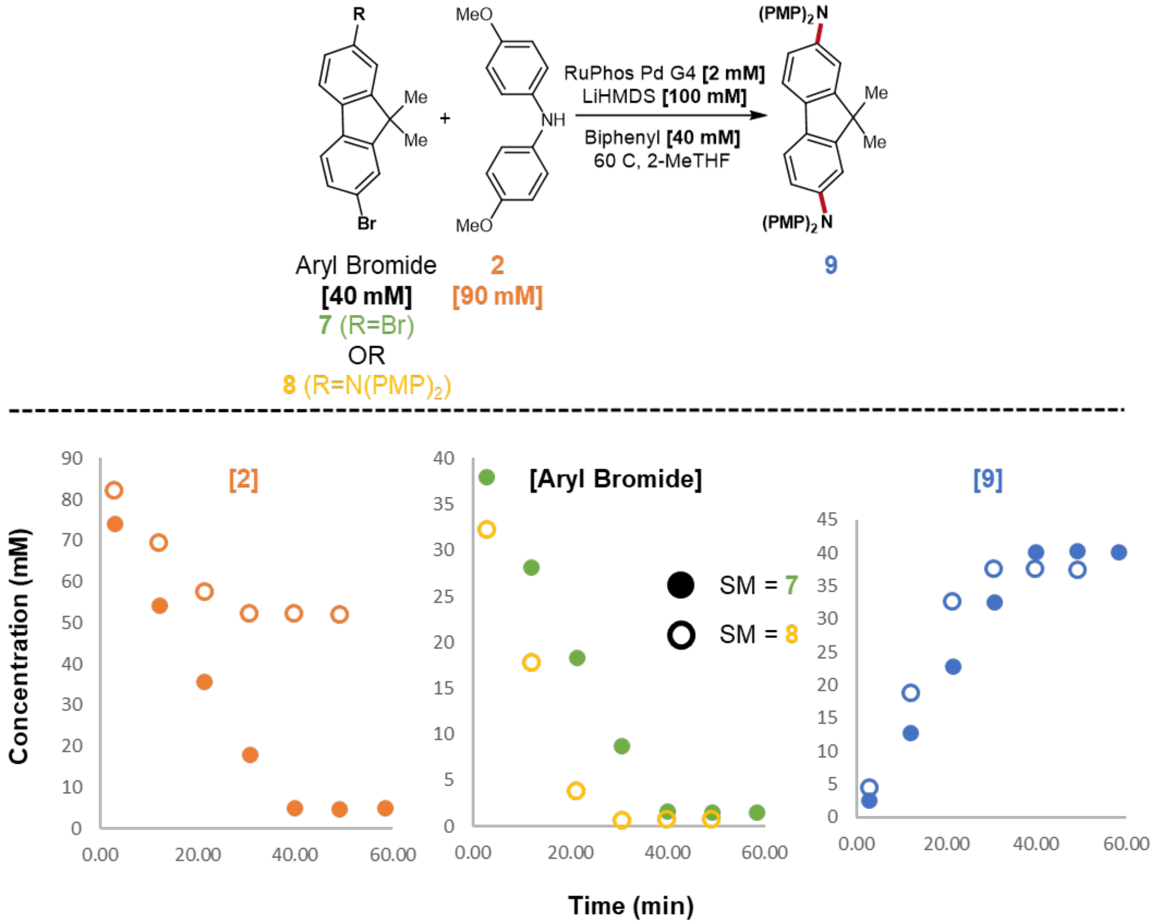

Supplementary Figure 21. Time course data of the amination of **7** (solid circles) and **8** (open circles) with RuPhos Pd G4 as the precatalyst.

Synthetic procedures

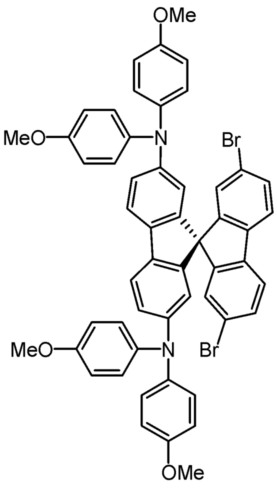

2',7'-dibromo-N2,N2,N7,N7-tetrakis(4- methoxyphenyl)-9,9'- spirobi[fluoren -e]-2,7-diamine (**4a**)

To a 15 mL 2-neck round bottom flask under an inert atmosphere was added LiHMDS (100 mg, 0.600 mmol), bis(4-methoxyphenyl)amine (**2**) (126 mg, 0.550 mmol), 2,2',7,7'-tetrabromo-9,9'-spirobi[fluorene] (**1**) (253 mg, 0.400 mmol), and PEPPSI-*IPr* (6.7 mg, 0.020 mmol). The flask was evacuated for five minutes and backfilled with argon three times before 10.0 mL of 2-MeTHF was added to the flask. The reaction was heated to 60 °C and allowed to stir for 1 hour. The reaction was quenched with saturated aqueous ammonium chloride. The crude was extracted with ethyl acetate (10 mL, 3x). The organic extracts were washed with brine, dried ($MgSO_4$), filtered, and concentrated under reduced pressure. The crude residue was purified *via* flash chromatography (15% ethyl acetate in petroleum ether with 1% triethylamine) to afford **1** (102 mg, 0.160 mmol) as a white solid and **4a** (136 mg, 0.150 mmol, 63% yield based on the recovered starting material **1**).

^{1}H NMR: (400 MHz, THF-d_8) δ 7.58 (dd, *J* = 27.5, 8.2 Hz, 4H), 7.44 (dd, *J* = 8.1, 0.9 Hz, 2H), 6.96 (s, 2H), 6.82 (t, *J* = 8.4 Hz, 10H), 6.68 (d, *J* = 8.3 Hz, 8H), 6.30 (d, *J* = 2.6 Hz, 2H), 3.67 (s, 12H).

^{13}C NMR: (101 MHz, C_6D_6) δ 156.3, 151.6, 148.9, 148.8, 141.3, 139.7, 135.3, 131.2, 127.3, 126.5, 122.2, 122.1, 122.0, 120.5, 117.3, 115.0, 66.3, 54.9.

MS EI+: (*m/z* calc. for $C_{53}H_{40}Br_2N_2O_4$ $[M]^+$ = 926.13563); found = 926.13548

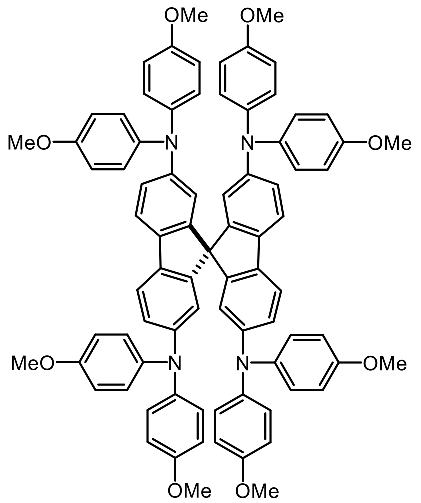

2,2',7,7'-tetrakis[N,N-di(4-methoxyphenyl)amino]-9,9'-spirobifluorene) (Spiro-OMeTAD, **6**)

To a vial under an inert atmosphere was added bis(4-methoxyphenyl)amine (**2**) (1.03 g, 4.50 mmol), LiHMDS (837 mg, 5.00 mmol), 2,2',7,7'-tetrabromo-9,9'-spirobi[fluorene] (**1**) (632 mg, 1.00 mmol), THF (18.0 mL), and toluene (1.6 mL) and the resultant solution was heated to 60 °C. $Pd(OAc)_2$ (11 mg, 0.050 mmol) and P(*t*Bu)$_3$ (20 mg, 0.10 mmol) in toluene (0.4 mL) were added via syringe to initiate the reaction. After 30 hours, the reaction was cooled to room temperature and quenched with saturated aqueous ammonium chloride. The crude was extracted with ethyl acetate (20 mL, 3x). The organic extracts were washed with brine, dried (Na_2SO_4), filtered, and concentrated under reduced pressure. Methanol (150 mL) was added to the crude residue to precipitate out the product. The resulting heterogeneous solution was triturated *via* sonicator. The mixture was then vacuum filtered, and the cake was washed with methanol followed by petroleum ether, then dried under vacuum to afford the purified product (1.12 g, 0.910 mmol, 91%) as an off-white solid. Characterization data is consistent with that in the literature.[6]

^{1}H NMR: (400 MHz, THF-d$_8$) δ 7.39 (d, *J* = 8.2 Hz, 4H), 6.91 (d, *J* = 9.1 Hz, 16H), 6.77 (d, *J* = 9.2 Hz, 20H), 6.54 (d, *J* = 2.1 Hz, 4H), 3.71 (s, 24H).

^{13}C NMR: (101 MHz, THF-d$_8$) δ 156.61, 151.07, 148.51, 142.35, 136.24, 126.15, 123.11, 120.61, 118.36, 115.26, 67.45, 55.59.

The quaternary spirocyclic center carbon signal overlaps with the THF-d$_8$ signal at 67.45 ppm. The presence of the carbon signal was confirmed by HMBC (Supplementary Figure 30). A clear correlation between a carbon signal at 67.45 ppm and the signal of the spirocyclic core protons three bonds away can be seen in the inset on the HMBC spectrum. This correlation cannot arise from a carbon nucleus in THF.

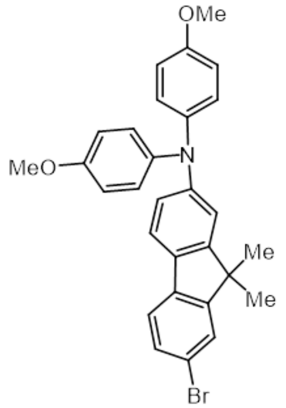

7-bromo-N,N-bis(4-methoxyphenyl)-9,9-dimethyl-9H-fluoren-2-amine (**8**)

To an oven-dried 15 mL two-neck round bottom flask in the glovebox was added LiHMDS (167 mg, 1.00 mmol) and a magnetic stir bar. The flask was sealed with two rubber septa, removed from the glovebox, and put under argon on a Schlenk line. Under positive pressure, one septum was removed and an 2,7-dibromo-9,9-dimethyl-9H-fluorene (**7**) (141 mg, 0.400 mmol), bis(4-methoxyphenyl)amine (206 mg, 0.900 mmol), and biphenyl (62 mg, 0.40 mmol) were added to the reaction flask. The flask was resealed with a septum and the system was evacuated for five minutes and backfilled with argon three times. Then, under a high flow of argon one septum was removed and the EasySampler probe was inserted into one neck of the round bottom flask. Next, 8.5 mL of 2-MeTHF was added to the reaction flask. The reaction was heated to 40 °C. Meanwhile, a precatalyst solution was prepared by adding XantPhos Pd G4 (29 mg, 0.030 mmol) to an oven-dried 10 mL vial. The vial was evacuated for five minutes and backfilled with argon three times before 2.25 mL of 2-MeTHF was added to the vial. The solution was sonicated for 60 seconds before 1.5 mL of the precatalyst solution containing XantPhos Pd G4 (19 mg, 0.020 mmol) was added to the reaction flask. The reaction was quenched with saturated aqueous ammonium chloride after 10 min when there was a substantial concentration of **8**. The crude was extracted with ethyl acetate (10 mL, 3x). The organic extracts were washed with brine, dried ($MgSO_4$), filtered, and concentrated under reduced pressure. The crude residue was purified *via* flash chromatography (5 to15% ethyl acetate in hexanes with 1% triethylamine) to afford **8** (68 mg, 0.137 mmol, 34% yield) as a white solid. Characterization data is consistent with that in the literature.[7]

^{1}H NMR: (300 MHz, DMSO-d$_6$) δ 7.69 (d, J = 1.6 Hz, 1H), 7.60 (dd, J = 8.2, 2.3 Hz, 2H), 7.44 (dd, J = 8.1, 1.7 Hz, 1H), 7.03 (d, J = 8.9 Hz, 4H), 6.93 (dd, J = 11.2, 5.5 Hz, 5H), 6.72 (dd, J = 8.3, 2.1 Hz, 1H), 3.74 (s, 6H), 1.32 (s, 6H).

^{13}C NMR: (75 MHz, DMSO-d_6) δ 155.65, 155.33, 154.39, 148.61, 140.31, 137.96, 129.93, 129.86, 126.47, 125.84, 121.11, 120.90, 119.01, 118.92, 114.94, 113.76, 55.22, 46.65, 26.61.

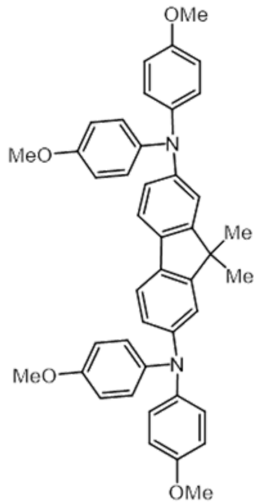

N2,N2,N7,N7-tetrakis(4-methoxyphenyl)-9,9-dimethyl-9H-fluorene-2,7-diamine (**9**)
To an oven-dried 15 mL two-neck round bottom flask in the glovebox was added LiHMDS (167 mg, 1.00 mmol) and a magnetic stir bar. The flask was sealed with two rubber septa, removed from the glovebox, and put under argon on a Schlenk line. Under positive pressure, one septum was removed and 2,7-dibromo-9,9-dimethyl-9H-fluorene (**7**) (141 mg, 0.400 mmol), bis(4-methoxyphenyl)amine (**2**) (206 mg, 0.900 mmol), and biphenyl (62 mg, 0.40 mmol) were added to the reaction flask. The flask was resealed with a septum and the system was evacuated for five minutes and backfilled with argon three times. Then, under a high flow of argon one septum was removed and the EasySampler probe was inserted into one neck of the round bottom flask. Next, 9.75 mL of 2-MeTHF was added to the reaction flask. The reaction was heated to 60 °C. Meanwhile, a precatalyst solution was prepared by adding PEPPSI-*IPr* (20 mg, 0.030 mmol) to an oven-dried 1.5 mL vial. The vial was evacuated for five minutes and backfilled with argon three times before 0.75 mL of 2-MeTHF was added to the vial. The reaction was initiated by transferring 0.5 mL of the precatalyst solution, containing PEPPSI-*IPr* (14 mg, 0.020 mmol), to the reaction flask. The reaction was quenched with saturated aqueous ammonium chloride after 60 minutes. The crude was extracted with ethyl acetate (10 mL, 3x). The organic extracts were washed with brine, dried ($MgSO_4$), filtered, and concentrated under reduced pressure. The crude residue was purified *via* flash chromatography (15% ethyl acetate in petroleum ether with 1% triethylamine) to afford **9** (153 mg, 0.235 mmol, 59% yield) as a white solid. Characterization data is consistent with that in the literature.[8]

^{1}H NMR: (300 MHz, THF-d_8) δ 7.38 (d, J = 8.2 Hz, 2H), 7.00 (m, 10H), 6.80 (d, J = 9.0 Hz, 10H), 3.74 (s, 12H), 1.25 (s, 6H).

^{13}C NMR: (75 MHz, THF-d_8) δ 156.87, 155.48, 148.53, 142.48, 133.53, 126.86, 121.33, 120.24, 116.53, 115.36, 55.58, 47.33, 27.44.

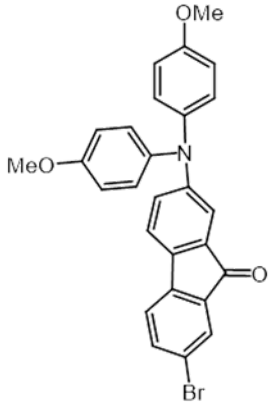

2-(bis(4-methoxyphenyl)amino)-7-bromo-9H-fluoren-9-one (**11**)
To an oven-dried 15 mL two-neck round bottom flask in the glovebox was added LiHMDS (167 mg, 1.00 mmol) and a magnetic stir bar. The flask was sealed with two rubber septa, removed from the glovebox, and put under argon on a Schlenk line. Under positive pressure, one septum was removed and an 9H-fluoren-9-one (**10**) (135 mg, 0.400 mmol), bis(4-methoxyphenyl)amine (**2**) (206 mg, 0.900 mmol), and biphenyl (62 mg, 0.40 mmol) were added to the reaction flask. The flask was resealed with a septum and the system was evacuated for five minutes and backfilled with argon three times. Then, under a high flow of argon one septum was removed and the EasySampler probe was inserted into one neck of the round bottom flask. Next, 8.5 mL of 2-MeTHF was added to the reaction flask. The reaction was heated to 25 °C. Meanwhile, a precatalyst solution was prepared by adding XantPhos Pd G4 (29 mg, 0.030 mmol) to an oven-dried 10 mL vial. The vial was evacuated for five minutes and backfilled with argon three times before 2.25 mL of 2-MeTHF was added to the vial. The solution was sonicated for 60 seconds before 1.5 mL of the precatalyst solution containing XantPhos Pd G4 (19 mg, 0.020 mmol) was added to the reaction flask. The reaction was quenched with saturated aqueous ammonium chloride after 10 min when there was a substantial concentration of **11**. The crude was extracted with ethyl acetate (10 mL, 3x). The organic extracts were washed with brine, dried ($MgSO_4$), filtered, and concentrated under reduced pressure. The crude residue was purified *via* flash chromatography (15% ethyl acetate in petroleum ether with 1% triethylamine) to afford **11** (55 mg, 0.11 mmol, 29% yield) as a green solid.

^{1}H NMR: (300 MHz, THF-d_8) δ 7.63 – 7.56 (m, 2H), 7.39 (dd, J = 11.0, 8.0 Hz, 2H), 7.06 (dd, J = 8.8, 2.1 Hz, 5H), 6.94 (dd, J = 8.2, 2.4 Hz, 1H), 6.91 – 6.84 (m, 4H), 3.76 (s, 6H).

^{13}C NMR: (75 MHz, THF-d_8) δ 192.07, 158.02, 151.70, 144.94, 140.82, 137.96, 137.03, 136.05, 135.21, 128.06, 127.62, 124.98, 122.26, 121.98, 121.67, 115.78, 115.56, 55.65.
MS ESI+: (*m/z* calc. for $C_{27}H_{20}BrNO_3$ $[M]^+$ = 485.0627); found = 485.0623

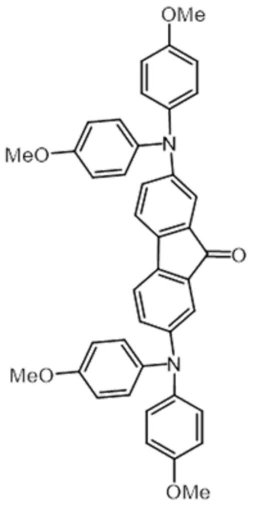

2,7-bis(bis(4-methoxyphenyl)amino)-9H-fluoren-9-one (**12**)
To an oven-dried 15 mL two-neck round bottom flask in the glovebox was added LiHMDS (167 mg, 1.00 mmol) and a magnetic stir bar. The flask was sealed with two rubber septa, removed from the glovebox, and put under argon on a Schlenk line. Under positive pressure, one septum was removed and an 9H-fluoren-9-one (**10**) (135 mg, 0.400 mmol), bis(4-methoxyphenyl)amine (**2**) (206 mg, 0.900 mmol), and biphenyl (62 mg, 0.40 mmol) were added to the reaction flask. The flask was resealed with a septum and the system was evacuated for five minutes and backfilled with argon three times. Then, under a high flow of argon one septum was removed and the EasySampler probe was inserted into one neck of the round bottom flask. Next, 9.75 mL of 2-MeTHF was added to the reaction flask. The reaction was heated to 60 °C. Meanwhile, a precatalyst solution was prepared by adding PEPPSI-*IPr* (20 mg, 0.030 mmol) to an oven-dried 1.5 mL vial. The vial was evacuated for five minutes and backfilled with argon three times before 0.75 mL of 2-MeTHF was added to the vial. The reaction was initiated by transferring 0.5 mL of the precatalyst solution, containing PEPPSI-*IPr* (14 mg, 0.020 mmol), to the reaction flask. The reaction was quenched with saturated aqueous ammonium chloride after 20 minutes. The crude was extracted with ethyl acetate (10 mL, 3x). The organic extracts were washed with brine, dried ($MgSO_4$), filtered, and concentrated under reduced pressure. The crude residue was purified *via* flash chromatography (15% ethyl acetate in petroleum ether with 1% triethylamine) to afford **9** (153 mg, 0.241 mmol, 60% yield) as a purple solid. Characterization data is consistent with that in the literature.[9]
^{1}H NMR: (300 MHz, THF-d_8) δ 7.22 (d, *J* = 8.1 Hz, 2H), 7.02 (dd, *J* = 9.2, 2.5 Hz, 10H), 6.90 (dd, *J* = 8.1, 2.4 Hz, 2H), 6.87 – 6.81 (m, 8H), 3.75 (s, 12H).

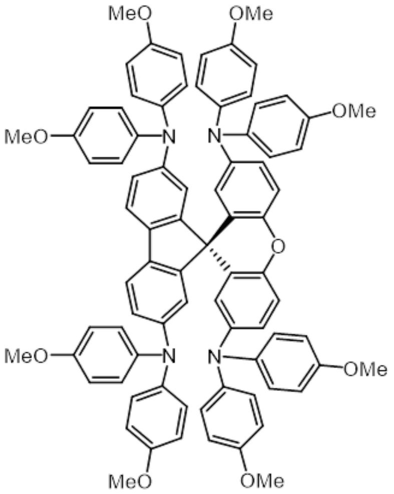

N2,N2,N2',N2',N7,N7,N7',N7'-octakis(4-methoxyphenyl)spiro[fluorene-9,9'-xanthene]-2,2',7,7'-tetraamine (**17**)
To an oven-dried 16 mL vial in the glovebox with a stir bar was added LiHMDS (306 mg, 1.83 mmol),2,2',7,7'-tetrabromospiro[fluorene-9,9'-xanthene] (**13**) (237, 0.366 mmol), and bis(4-methoxyphenyl)amine (**2**) (377 mg, 1.65 mmol) and PEPPSI-*IPr* (24.9 mg, 3.66 x 10^{-2} mmol) were added to the reaction flask. The flask was resealed with a septum and brough out of the glovebox upon which 10 mL of MeTHF was added under a positive pressure of argon. The reaction was heated to 60 °C and stirred for 3 hours. The reaction was quenched with saturated aqueous ammonium chloride. The crude was extracted with ethyl acetate (10 mL, 3x). The organic extracts were washed with brine, dried ($MgSO_4$), filtered, and concentrated under reduced pressure. The crude residue was purified *via* flash chromatography (20% ethyl acetate in petroleum ether to 50% ethyl acetate in petroleum ether with 1% triethylamine) to afford **17** (240 mg, 0.193 mmol, 53% yield) as a lightbrown solid. Characterization data is consistent with that in the literature.[10]
^{1}H NMR: (400 MHz, THF-d_8) δ 7.28 (d, *J* = 8.3 Hz, 2H), 6.93 – 6.88 (m, 11H), 6.82 (d, *J* = 9.2 Hz, 8H), 6.79 – 6.70 (m, 21H), 6.38 (d, *J* = 2.7 Hz, 2H), 3.72 (s, 12H), 3.67 (s, 12H).
^{13}C NMR: (101 MHz, THF-d_8) δ 156.79, 156.27, 156.03, 148.83, 147.75, 144.69, 142.45, 142.04, 133.74, 126.85, 126.60, 125.44, 124.42, 122.81, 122.10, 120.46, 118.81, 118.05, 115.35, 115.24, 55.62, 55.61, 55.61.
The quaternary spirocyclic carbon signal overlaps with the methoxy carbon signals at 55.62 and 55.61 ppm. The presence of the quaternary carbon signal was confirmed by HMBC (Supplementary Figure 46). A clear correlation between a carbon signal at 55.6 ppm and the signal of aromatic protons can be seen in the inset on the HMBC spectrum. These correlations cannot arise from a carbon nucleus in THF or from the methoxy carbons.

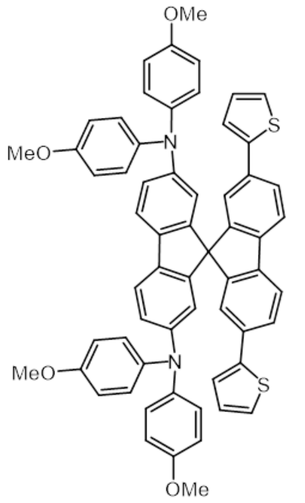

N2,N2,N7,N7-tetrakis(4-methoxyphenyl)-2',7'-di(thiophen-2-yl)-9,9'-spirobi[fluorene]-2,7-diamine (**18**)

To a 16 mL vial was added **4a** (232 mg, 0.250 mmol), 3-thiophenylboronic acid (96 mg, 0.75 mmol), and tripotassium phosphate (212 mg, 1.00 mmol). The flask was evacuated for five minutes and backfilled with argon three times before 2.0 mL of THF and 0.2 mL of degassed water were added to the vial. The reaction was heated to 65 °C before XPhos Pd G2 (9.4 mg, 0.012 mmol) was added. After 2.5 hours, the reaction was cooled to room temperature and the THF was evaporated *in vacuo*. The reaction residue was taken up in 5 mL of water and extracted with DCM (5 mL, 3x). The combined organic layers were filtered through celite and then concentrated *in vacuo* to afford **18** (213 mg, 0.230 mmol, 92% yield) as a brown solid. A minor impurity was identified as the debrominated monocoupled product based on HPLC-MS (spectra and structure shown in Supplementary Figure 48 and Supplementary Figure 49) and was quantified by integration in the ^{1}H NMR (Supplementary Figure 47). The integration of the methyl peak in the impurity was less than 5% of the integration of the methyl peak of **18**.

^{1}H NMR: (400 MHz, THF-d_8) δ 7.71 (d, *J* = 8.0 Hz, 2H), 7.63 – 7.54 (m, 6H), 7.42 – 7.36 (m, 4H), 7.11 (s, 2H), 6.69 (dd, *J* = 81.9, 9.0 Hz, 18H), 6.41 (s, 2H), 3.61 (s, 12H).

^{13}C NMR: (101 MHz, THF-d_8) δ 156.79, 156.26, 156.03, 148.83, 147.75, 144.69, 142.45, 142.04, 133.74, 126.84, 126.59, 125.44, 124.41, 122.81, 122.11, 120.46, 118.82, 118.05, 115.35, 115.24, 67.45, 55.61.

The quaternary spirocyclic center carbon signal overlaps with the THF-d_8 signal at 67.45 ppm. The presence of the non-THF carbon signal was confirmed by HMBC (Supplementary Figure 51). Clear correlations between a carbon signal at 67.45 ppm and aromatic protons at 6.41 and 7.11 ppm can be seen in the inset on the HMBC spectrum. These correlations cannot arise from a carbon nucleus in THF.

MS ESI+: (*m/z* calc. for $C_{61}H_{46}N_2O_4S_2$ $[M]^+$ = 934.2899); found = 934.2898

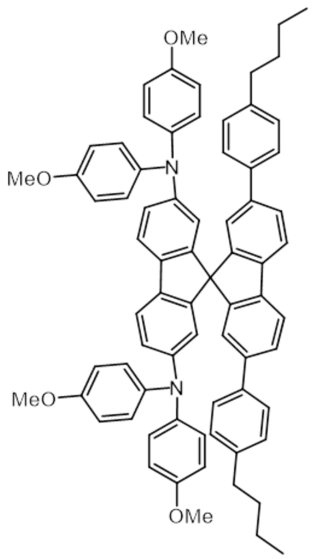

2',7'-bis(4-butylphenyl)-N2,N2,N7,N7-tetrakis(4-methoxyphenyl)-9,9'-spirobi[fluorene]-2,7-diamine (**19**)

To a 16 mL vial was added **4a** (279 mg, 0.300 mmol), (4-butylphenyl)boronic acid (214 mg, 1.20 mmol), and tripotassium phosphate (509 mg, 2.40 mmol). The flask was evacuated for five minutes and backfilled with argon three times before 1.2 mL of THF and 0.3 mL of degassed water were added to the vial. The reaction was heated to 50 °C before XPhos Pd G2 (11.8 mg, 0.0150 mmol) was added. After 19.5 hours, the reaction was cooled to room temperature, diluted with ethyl acetate, extracted with NaOH (5 mL 1 M, 1x), and extracted with brine (5 mL, 1x). The organic layer was dried with Na_2SO_4, concentrated *in vacuo*, and purified *via* flash chromatography (10% ethyl acetate in hexanes with 1% triethylamine) to afford **19** (272 mg, 0.263 mmol, 88% yield) as a brown solid.

^{1}H NMR: ^{1}H NMR (400 MHz, THF-d_8) δ 7.75 (d, *J* = 7.9 Hz, 2H), 7.60 – 7.53 (m, 4H), 7.44 (d, *J* = 8.2 Hz, 4H), 7.21 (d, *J* = 8.2 Hz, 4H), 7.07 (d, *J* = 1.8 Hz, 2H), 6.71 (dd, *J* = 82.5, 9.1 Hz, 18H), 6.45 (d, *J* = 2.2 Hz, 2H), 3.62 (s, 12H), 2.63 (4H), 1.61 (p, *J* = 7.5 Hz, 4H), 1.37 (dt, *J* = 15.0, 7.0 Hz, 4H), 0.93 (t, *J* = 7.4 Hz, 6H).

The chemical shift of water in THF-d_8 in the ^{1}H NMR spectrum of **19** is 2.62 ppm. The methylene protons closest to the aryl ring on the butyl chain also have a chemical shift around 2.62 ppm. Therefore, the peak for these protons could not be integrated and the coupling constant could not be determined. **19** has either poor solubility in alternative deuterated solvents or suffers from overlapping peaks. The ^{1}H NMR spectrum in THF-d_8 (Supplementary Figure 52) shows that the sample is pure. Additionally, the HMBC spectrum (Supplementary Figure 54) shows that the proton signal at 2.63 ppm correlates to two alkyl carbons and two aromatic carbons, as expected for the protons at that position on the alkyl chain. The HMBC spectrum confirms the identity of the overlapped protons, and the ^{1}H NMR spectrum shows that the integration and splitting for all other proton signals is correct.

^{13}C NMR: (101 MHz, THF-d_8) δ 156.67, 151.22, 150.81, 148.65, 142.66, 142.04, 141.78, 141.22, 139.64, 136.28, 129.56, 127.67, 127.30, 126.37, 122.86, 122.23, 121.22, 120.49, 118.28, 115.13, 67.46, 55.47, 36.09, 34.72, 23.21, 14.34.
The quaternary spirocyclic center carbon signal overlaps with the THF-d_8 signal at 67.46 ppm. The presence of the non-THF carbon signal was confirmed by HMBC (Supplementary Figure 54). Clear correlations between a carbon signal at 67.46 ppm and aromatic protons at 6.45 and 7.06 ppm can be seen in the inset on the HMBC spectrum. These correlations cannot arise from a carbon nucleus in THF.
MS ESI+: (*m/z* calc. for $C_{73}H_{66}N_2O_4$ $[M]^+$ = 1034.5023); found = 1034.5027

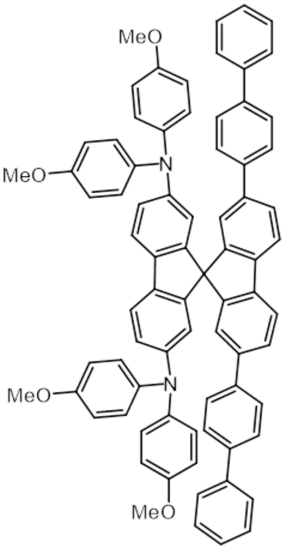

2',7'-di([1,1'-biphenyl]-4-yl)-N2,N2,N7,N7-tetrakis(4-methoxyphenyl)-9,9'-spirobi[fluorene]-2,7-diamine (20)
To a 16 mL vial was added **4a** (232 mg, 0.250 mmol), 4-biphenylboronic acid (149 mg, 0.750 mmol), and tripotassium phosphate (212 mg, 1.00 mmol). The flask was evacuated for five minutes and backfilled with argon three times before 2.0 mL of THF and 0.2 mL of degassed water were added to the vial. The reaction was heated to 65 °C before XPhos Pd G2 (9.4 mg, 0.012 mmol) was added. After 4 hours, the reaction was cooled to room temperature and the THF was evaporated *in vacuo*. The reaction residue was taken up in 5 mL of water and extracted with DCM (5 mL, 3x). The combined organic layers were filtered through celite and then concentrated *in vacuo*. The crude reaction mixture was purified *via* flash chromatography (15% ethyl acetate in petroleum ether with 1% triethylamine) to afford **20** (160 mg, 0.150 mmol, 60% yield) as a yellow solid.
^{1}H NMR: (400 MHz, THF-d_8) δ 7.81 (d, *J* = 7.9 Hz, 2H), 7.69 – 7.58 (m, 16H), 7.40 (t, *J* = 7.6 Hz, 4H), 7.29 (t, *J* = 8.0 Hz, 2H), 7.15 (d, *J* = 1.8 Hz, 2H), 6.84 – 6.79 (m, 10H), 6.63 – 6.58 (m, 8H), 6.47 (d, *J* = 2.2 Hz, 2H), 3.62 (s, 12H).
^{13}C NMR: (101 MHz, THF-d_8) δ 156.66, 151.36, 150.66, 148.66, 142.00, 141.57, 141.47, 141.29, 141.02, 140.87, 136.25, 129.55, 128.15, 128.03, 128.01, 127.59, 127.39, 126.35, 122.87, 122.24, 121.37, 120.90, 118.23, 115.10, 67.45, 55.42.
The quaternary spirocyclic center carbon signal overlaps with the THF-d_8 signal at 67.45 ppm. The presence of the non-THF carbon signal was confirmed by HMBC (Supplementary Figure 57). Clear correlations between a carbon signal at 67.45 ppm and aromatic protons at 6.47 and 7.15 ppm can be seen in the inset on the HMBC spectrum. These correlations cannot arise from a carbon nucleus in THF.
MS ESI+: (*m/z* calc. for $C_{77}H_{58}N_2O_4$ $[M]^+$ = 1074.4397); found = 1074.4406

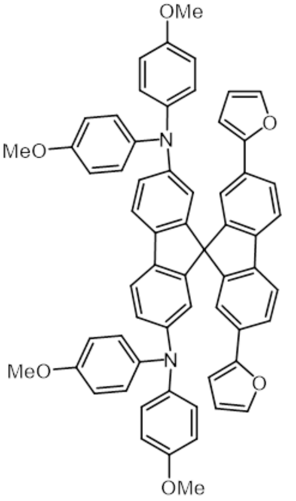

2',7'-di(furan-2-yl)-N2,N2,N7,N7-tetrakis(4-methoxyphenyl)-9,9'-spirobi[fluorene]-2,7-diamine (21)
To a 16 mL vial was added **4a** (279 mg, 0.300 mmol), 2-furanylboronic acid (134 mg, 1.20 mmol), and tripotassium phosphate (509 mg, 2.40 mmol). The flask was evacuated for five minutes and backfilled with argon three times before 1.2 mL of THF and 0.3 mL of degassed water were added to the vial. The reaction was heated to 50 °C before XPhos Pd G2 (11.8 mg, 0.0150 mmol) was added. After 19.5 hours, the reaction was cooled to room temperature, diluted with EtOAc, extracted with NaOH (5 mL 1 M, 1x), and extracted with brine (5 mL, 1x). The organic layer was dried with Na_2SO_4 and concentrated *in vacuo* to afford **21** (264 mg, 0.290 mmol, 97% yield by ^{1}H NMR) as a brown solid. Diethyl ether was present in the sample even after the sample was dried under vacuum, heated to 60 °C, and stirred overnight. Caffeine (19.5 mg, 0.100 mmol) was added to the ^{1}H NMR sample to determine the yield of **21**. The ^{1}H NMR spectrum with the internal standard is show in Supplementary Figure 59.
^{1}H NMR: (400 MHz, DMSO-d_6) δ 8.14 (s, 2H), 7.83 (d, *J* = 7.9 Hz, 2H), 7.71 – 7.57 (m, 6H), 6.89 (d, *J* = 11.5 Hz, 4H), 6.81 – 6.71 (m, 10H), 6.69 – 6.62 (m, 8H), 6.11 (d, *J* = 2.3 Hz, 2H), 3.61 (s, 12H).

^{13}C NMR: (75 MHz, DMSO-d_6) δ 155.22, 149.51, 149.17, 147.25, 144.31, 140.22, 139.51, 139.37, 134.46, 131.56, 125.77, 125.67, 125.49, 120.83, 120.38, 120.29, 120.07, 115.63, 114.58, 108.64, 65.39, 55.04.
The HMBC of **21** (Supplementary Figure 61) shows that the carbon signal at 65.39 ppm, which cannot arise from THF-d_8 since this spectrum was taken in DMSO-d_6, correlates to protons in the aromatic region at 6.11 and 6.89 ppm. This HMBC data substantiates our earlier conclusions that the spirocyclic carbon signal overlaps with the residual solvent peaks of THF-d_8 in the ^{13}C NMR spectra of compounds **6**, **17**, **18**, **19**, and **20**.
MS ESI+: (*m/z* calc. for $C_{61}H_{46}N_2O_6$ $[M]^+$ = 902.3356); found = 902.3360

Accompanying spectra can be found in the Spectra section (S).

Crystallographic Data[5]

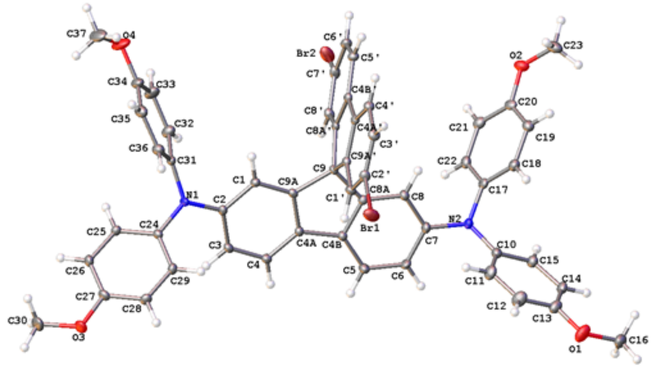

Supplementary Figure 22. Labeled crystal structure of **4a** obtained by single-crystal x-ray analysis.

Procedure and parameters for single-crystal x-ray analysis of 4a: A colourless blade-shaped crystal with dimensions 0.35 × 0.17 × 0.05 mm^3 was mounted on a mylar loop in oil. Data were collected using a Bruker APEX II area detector diffractometer equipped with a Kryoflex low-temperature device operating at T = 100(2) K.

Data were measured using *f* and *w* scans of 0.5 ° per frame for 10 s using MoK_a radiation (microfocus sealed X-ray tube, 50 kV, 0.99 mA). The total number of runs and images was based on the strategy calculation from the program APEX3. The maximum resolution that was achieved was *Q* = 30.427° (0.70 Å).

The unit cell was refined using **SAINT** (Bruker, V8.40A, after 2013) on 9731 reflections, 16% of the observed reflections. Data reduction, scaling and absorption corrections were performed using **SAINT** (Bruker, V8.40A, after 2013). The final completeness is 100.00 % out to 30.427° in Q.

A multi-scan absorption correction was performed using **SADABS**-2016/2 (Bruker, 2016/2) was used for absorption correction. wR_2(int) was 0.1056 before and 0.0460 after correction. The ratio of minimum to maximum transmission is 0.7767. The *l*/2 correction factor is not present. The absorption coefficient m of this material is 1.777 mm^{-1} at this wavelength (*l* = 0.71073Å) and the minimum and maximum transmissions are 0.711 and 0.915.

The structure was solved and the space group P2/*c* (# 13) determined by the **XT** (Sheldrick, 2015) structure solution program using Intrinsic Phasing methods and refined by full matrix least squares minimisation on $\boldsymbol{F^2}$ using version 2018/3 of **XL** (Sheldrick, 2015). The material crystallizes with both toluene and MeOH solvent in the lattice. There is one half-molecule of toluene, disordered about a two-fold rotational axis in the unit cell. The MeOH is disorder in two orientations and only partially occupied. Occupancies for each fragment were refined individually. All non-hydrogen atoms were refined anisotropically. Hydrogen atom positions were calculated geometrically and refined using the riding model.

Supplementary Table 3. Crystallographic parameters for **4a**.

Formula	$C_{56.98}H_{45.93}Br_2N_2O_{4.48}$
$D_{calc.}$/ g cm^{-3}	1.401
m/mm^{-1}	1.777

Formula Weight	990.78
Colour	colourless
Shape	blade
Size/mm^3	0.35×0.17×0.05
T/K	100(2)
Crystal System	monoclinic
Space Group	*P*2/*c*
a/Å	18.150(6)
b/Å	10.685(3)
c/Å	25.040(8)
a/°	90
b/°	104.687(10)
g/°	90
V/Å^3	4697(3)
Z	4
Z'	1
Wavelength/Å	0.71073
Radiation type	MoK_a
Q_{min}/°	1.160
Q_{max}/°	30.427
Measured Refl's.	61726
Ind't Refl's	14151
Refl's with I > 2(I)	11298
R_{int}	0.0452
Parameters	654
Restraints	1
Largest Peak	0.570
Deepest Hole	-0.583
GooF	1.015
wR_2 (all data)	0.0900
wR_2	0.0839
R_1 (all data)	0.0523
R_1	0.0358

Spectra

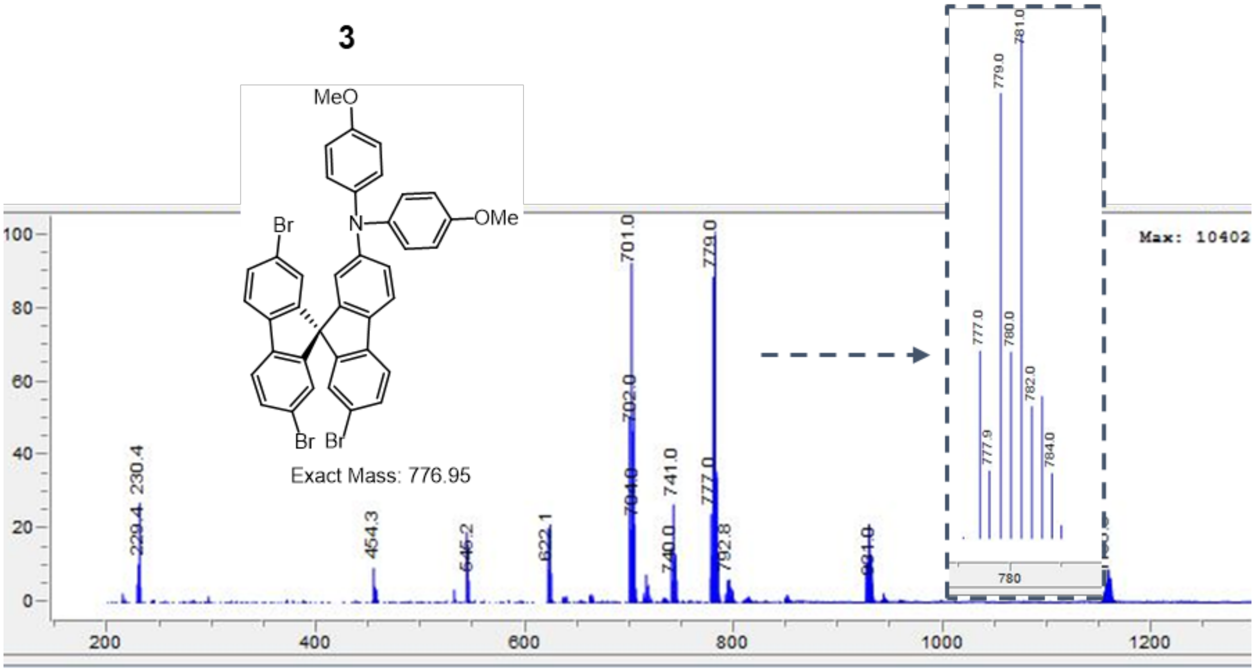

Supplementary Figure 23. LRMS of **3** (exact mass: 776.95 m/z, found: 777.0 m/z).

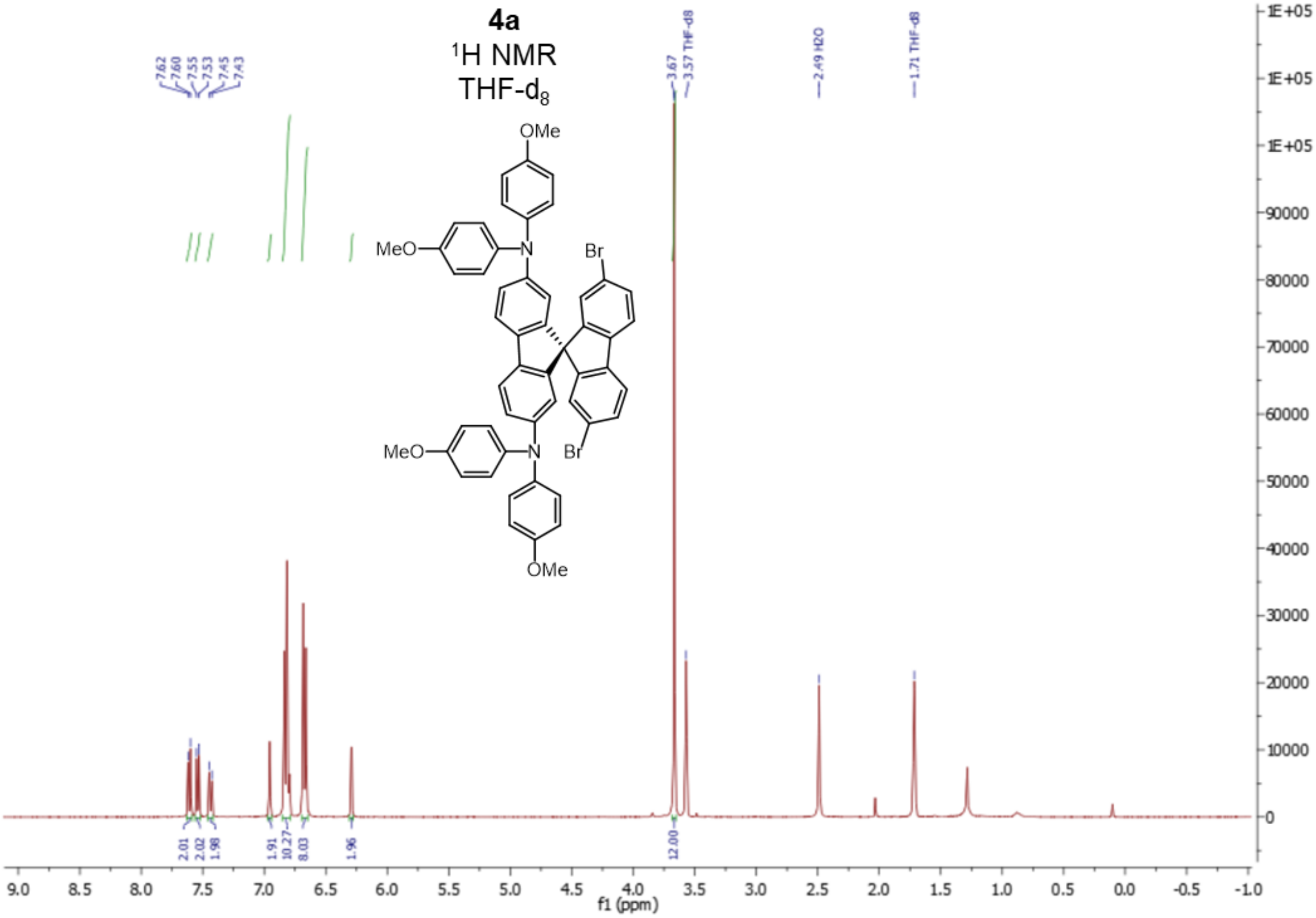

Supplementary Figure 24. ^{1}H NMR (400 MHz, THF-d$_8$) spectrum of **4a**.

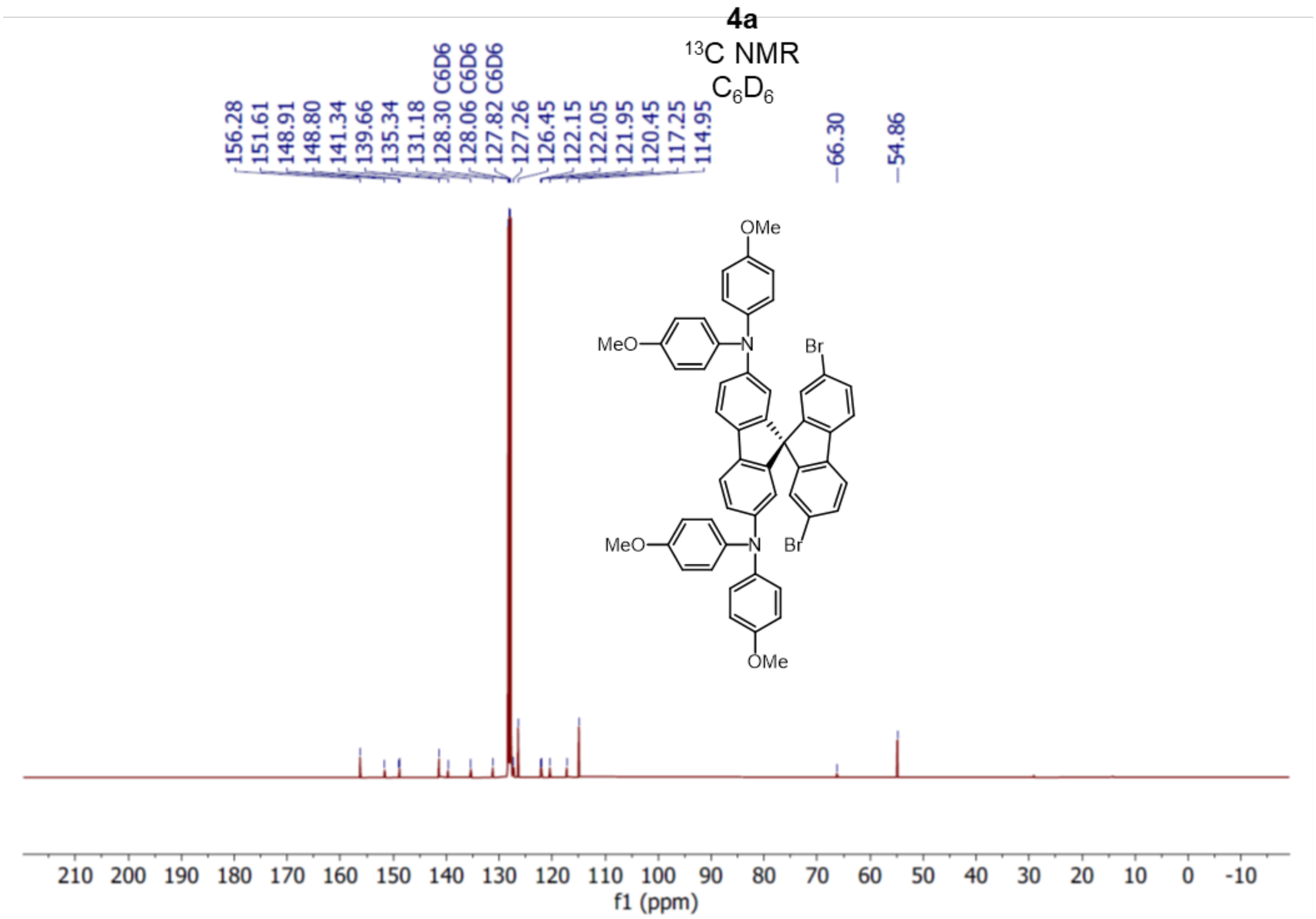

Supplementary Figure 25. ^{13}C NMR (101 MHz, C_6D_6) spectrum of **4a**.

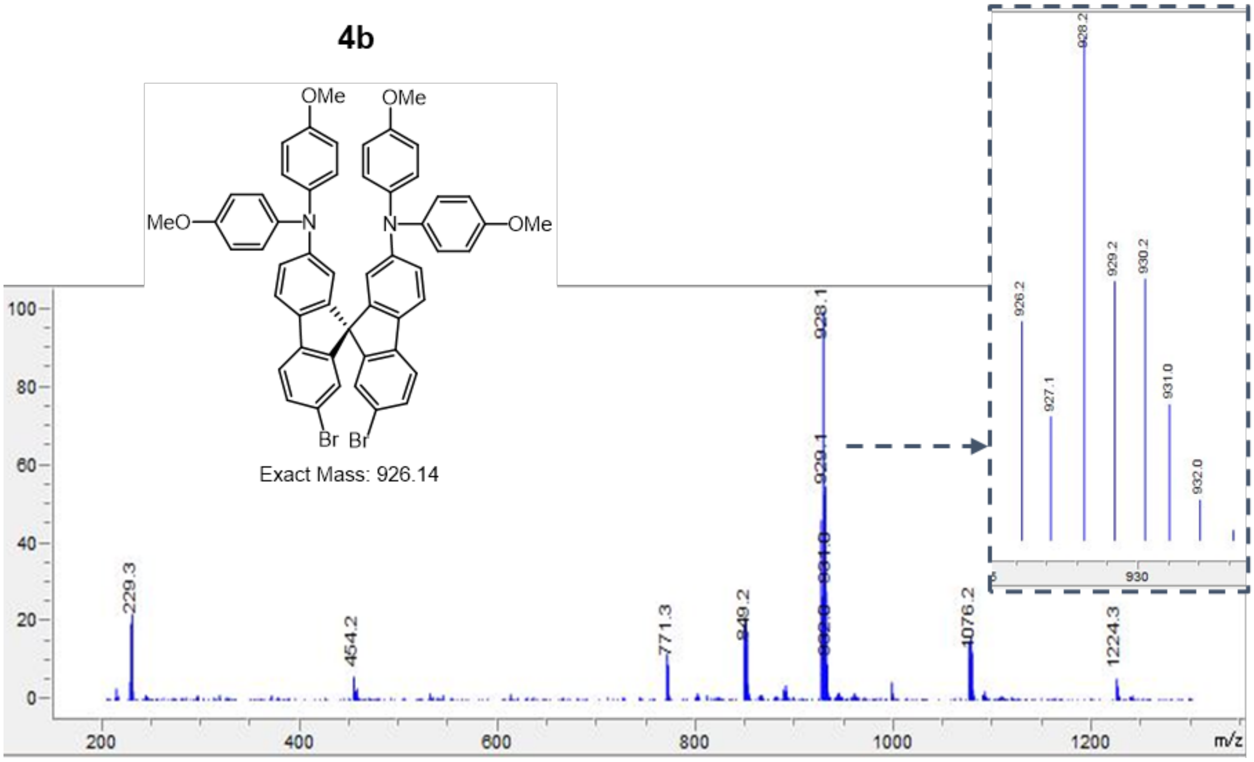

Supplementary Figure 26. LRMS of **4b** (exact mass: 926.14 m/z, found: 926.2 m/z).

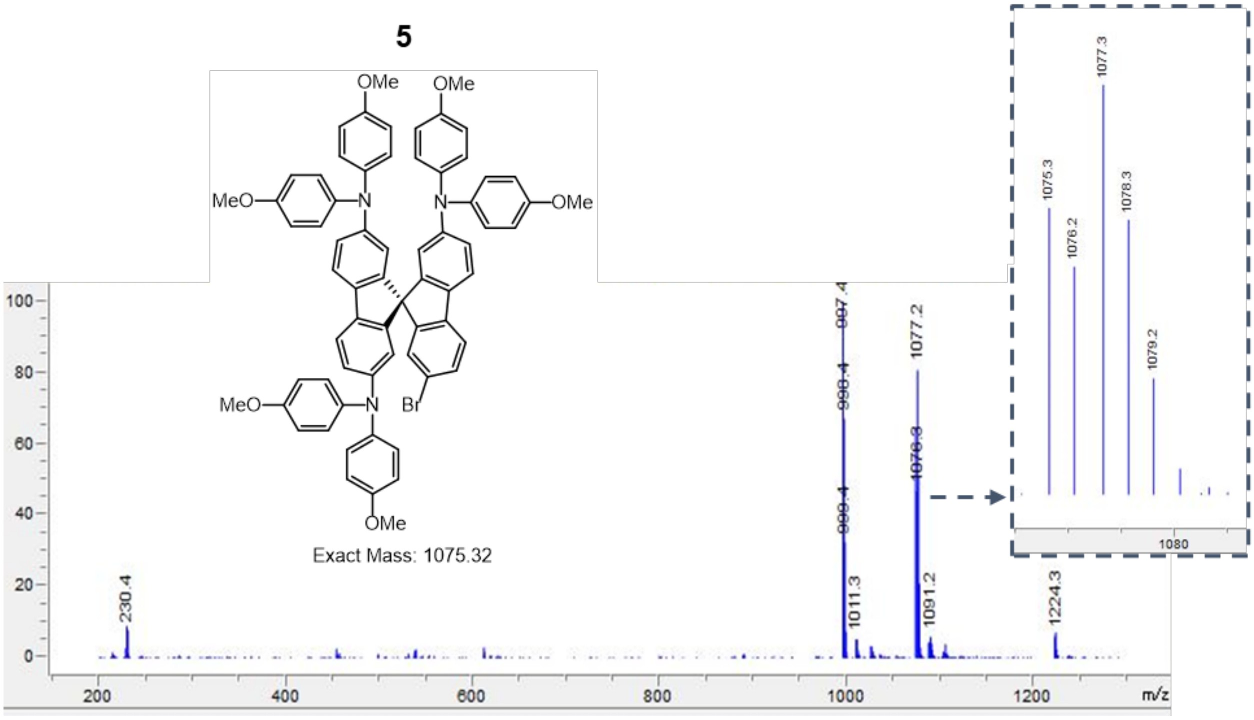

Supplementary Figure 27. LRMS of **5** (exact mass: 1075.32 m/z, found: 1075.3 m/z).

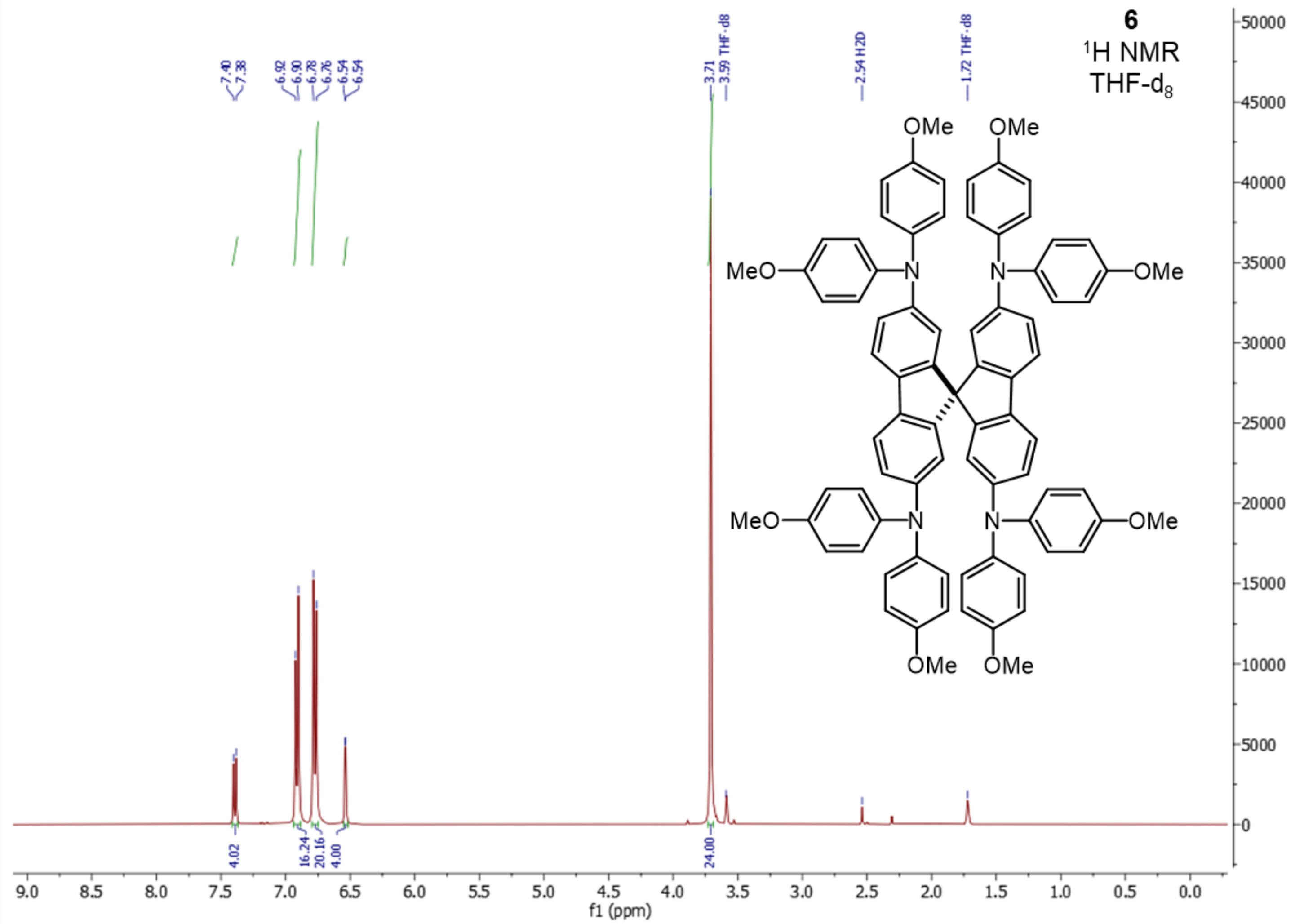

Supplementary Figure 28. ^{1}H NMR (400 MHz, THF-d$_8$) spectrum of **6**.

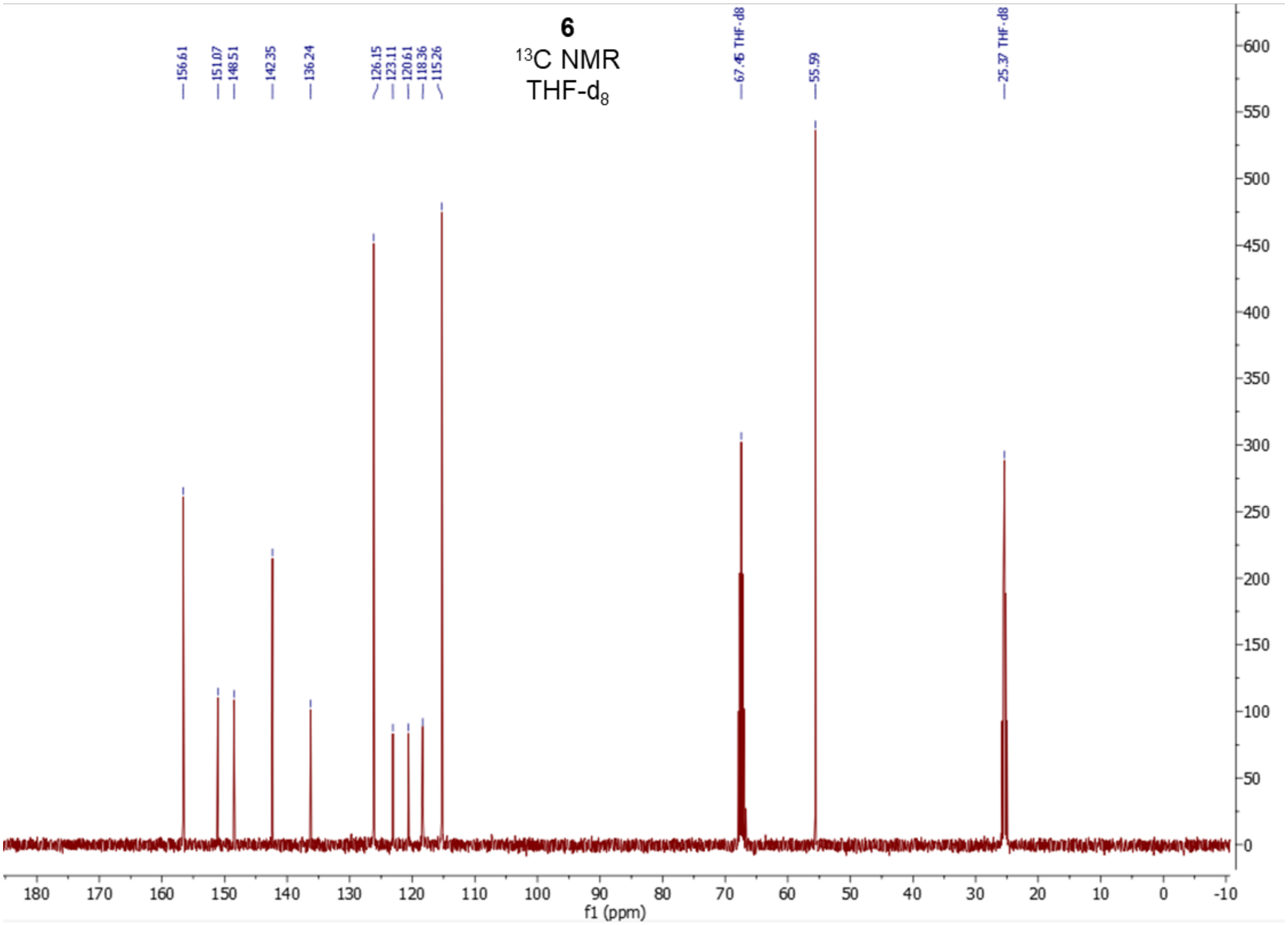

Supplementary Figure 29. ^{13}C NMR (101 MHz, THF-d_8) spectrum of **6**.

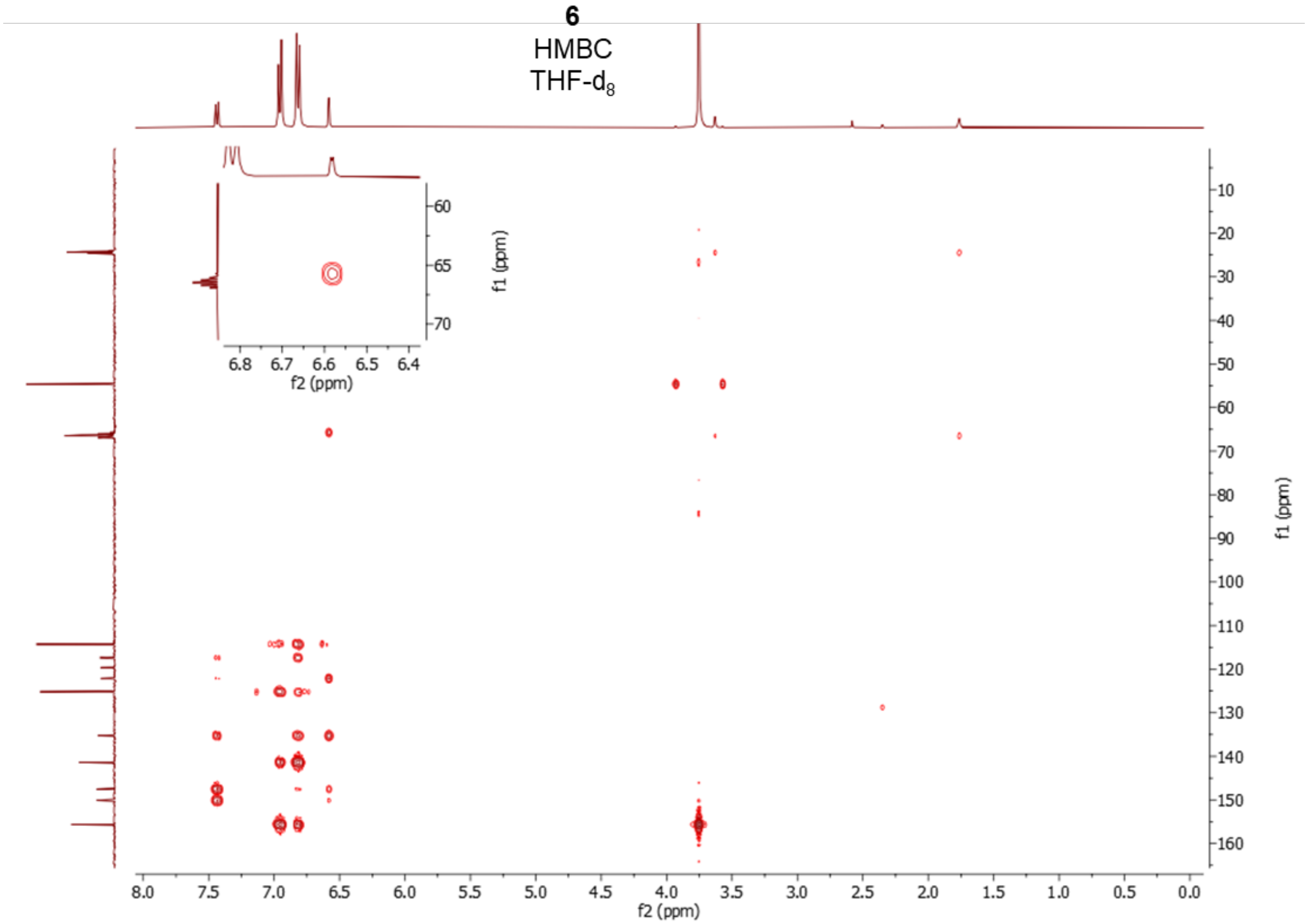

Supplementary Figure 30. HMBC NMR (^{1}H: 400 MHz, ^{13}C: 101 MHz, THF-d$_8$) spectrum of **6**.

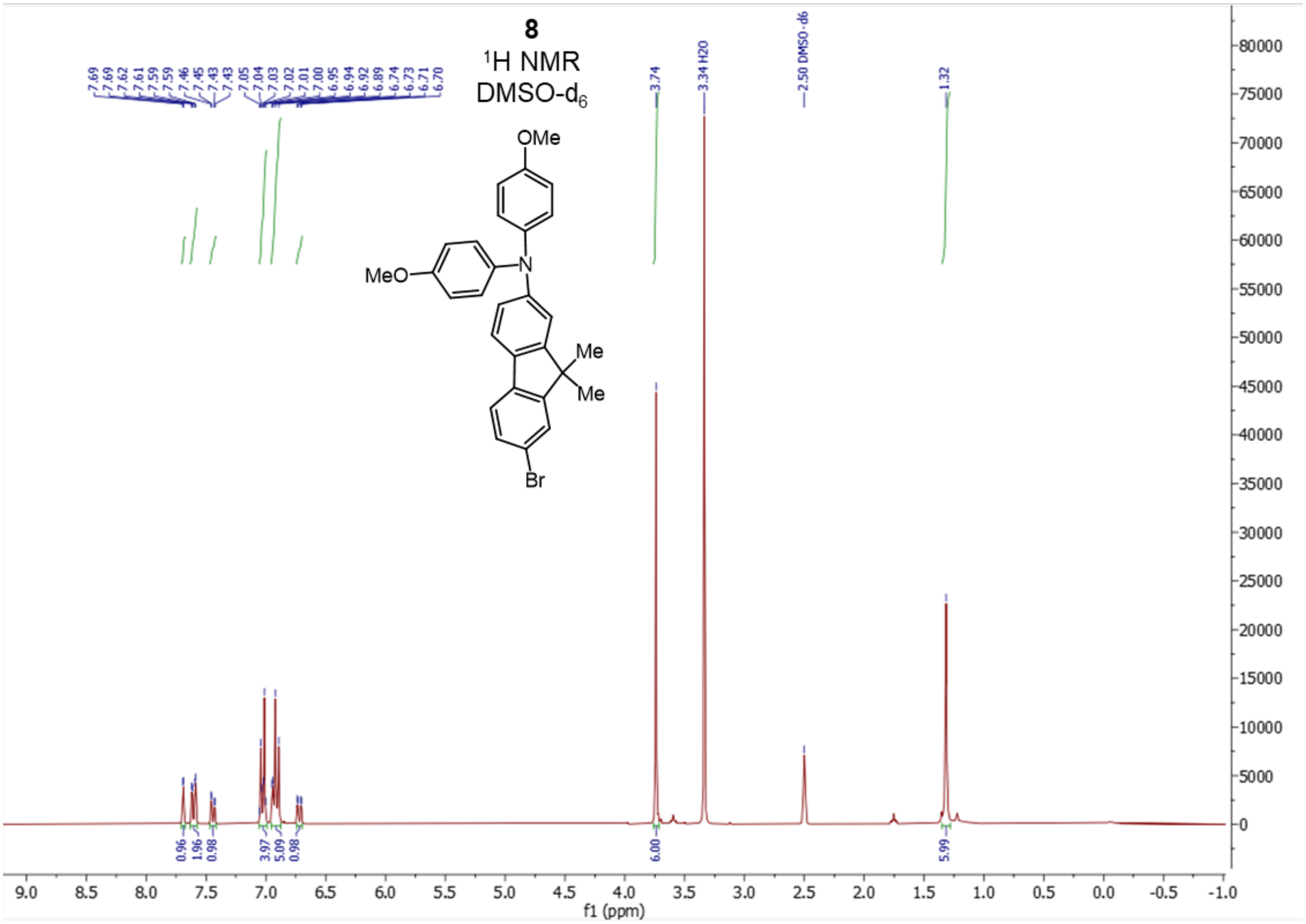

Supplementary Figure 31. ^{1}H NMR (300 MHz, DMSO-d_6) spectrum of **8**.

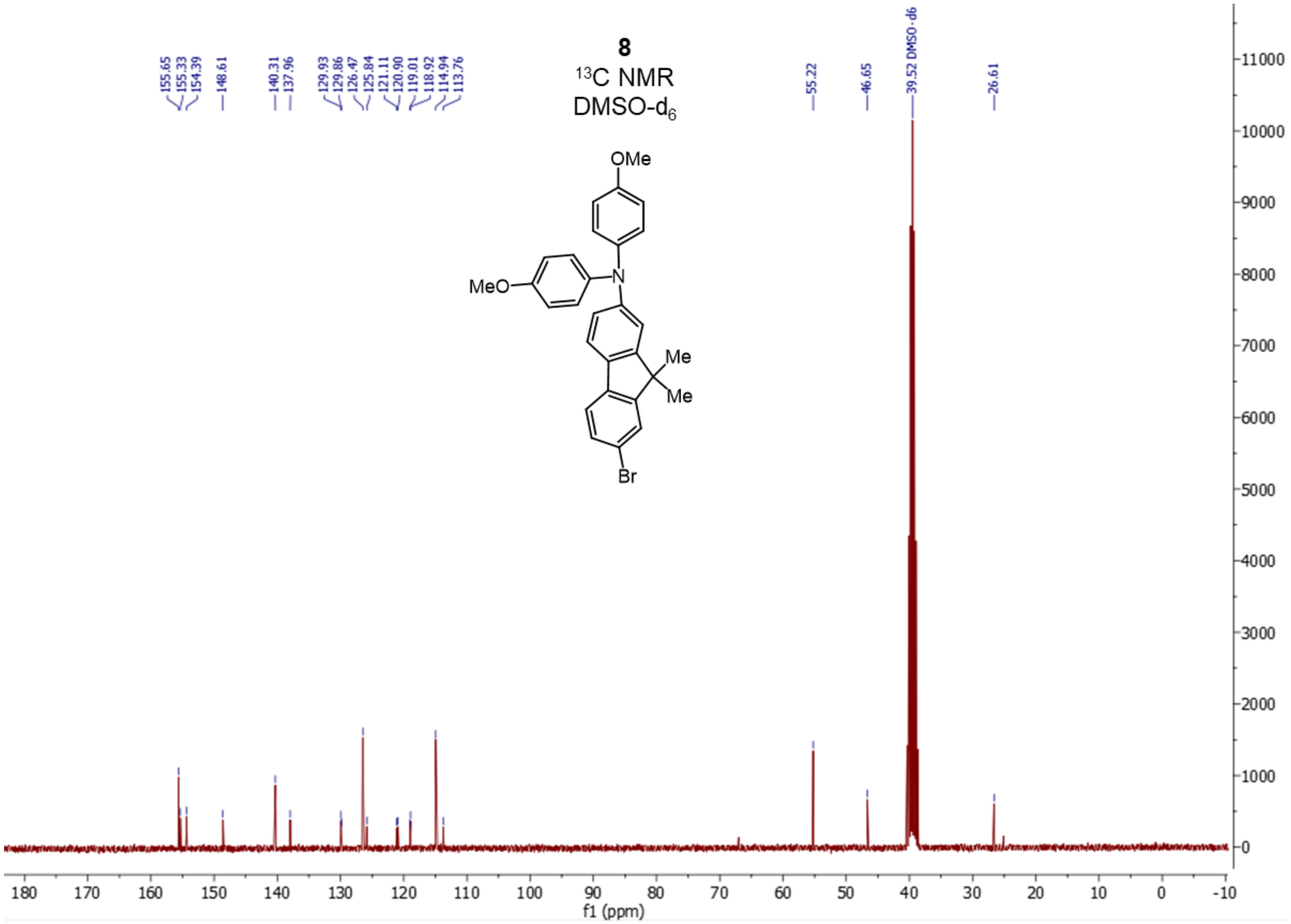

Supplementary Figure 32. ^{13}C NMR (75 MHz, DMSO-d_6) spectrum of **8**.

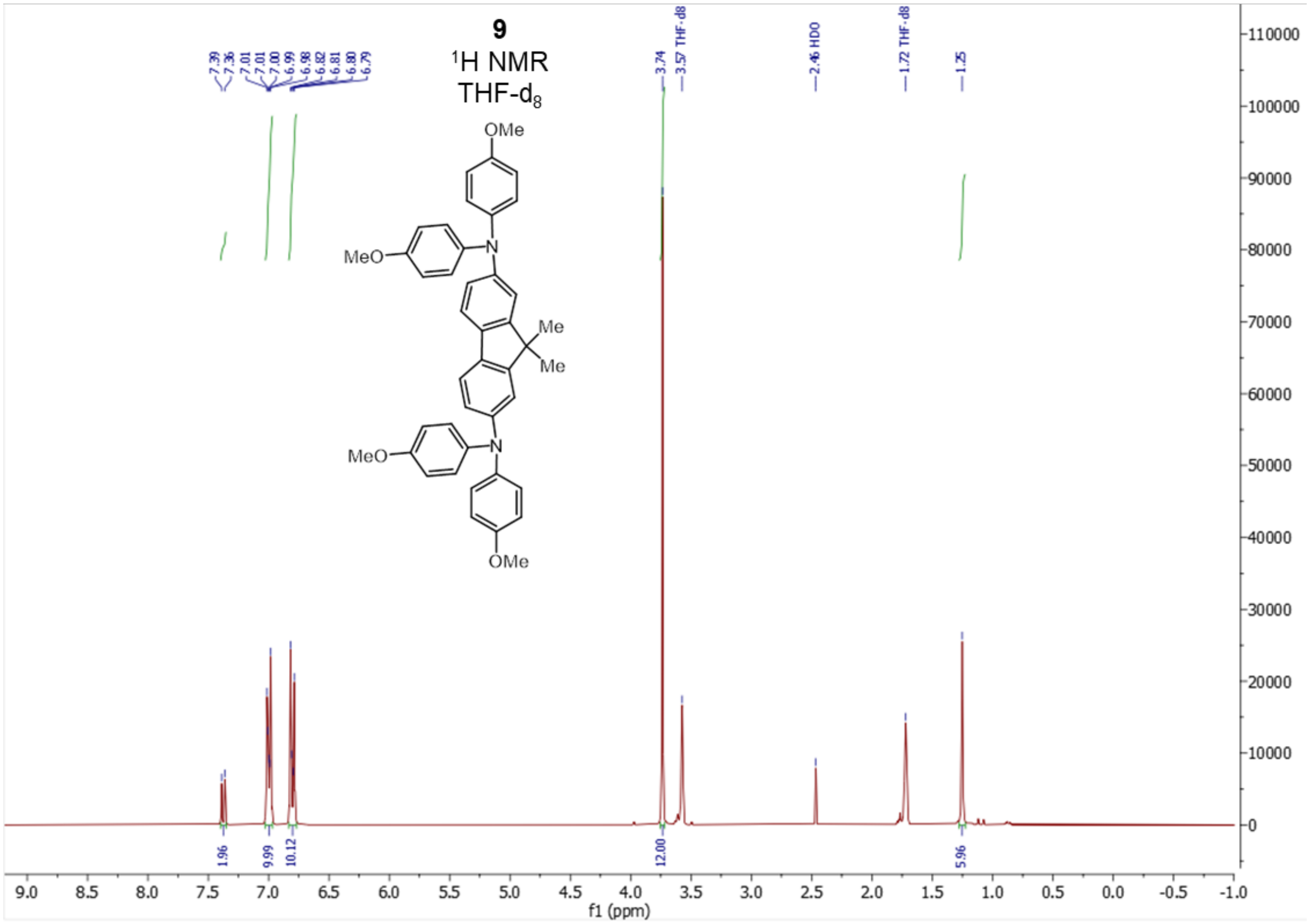

Supplementary Figure 33. ^{1}H NMR (300 MHz, THF-d_8) spectrum of **9**.

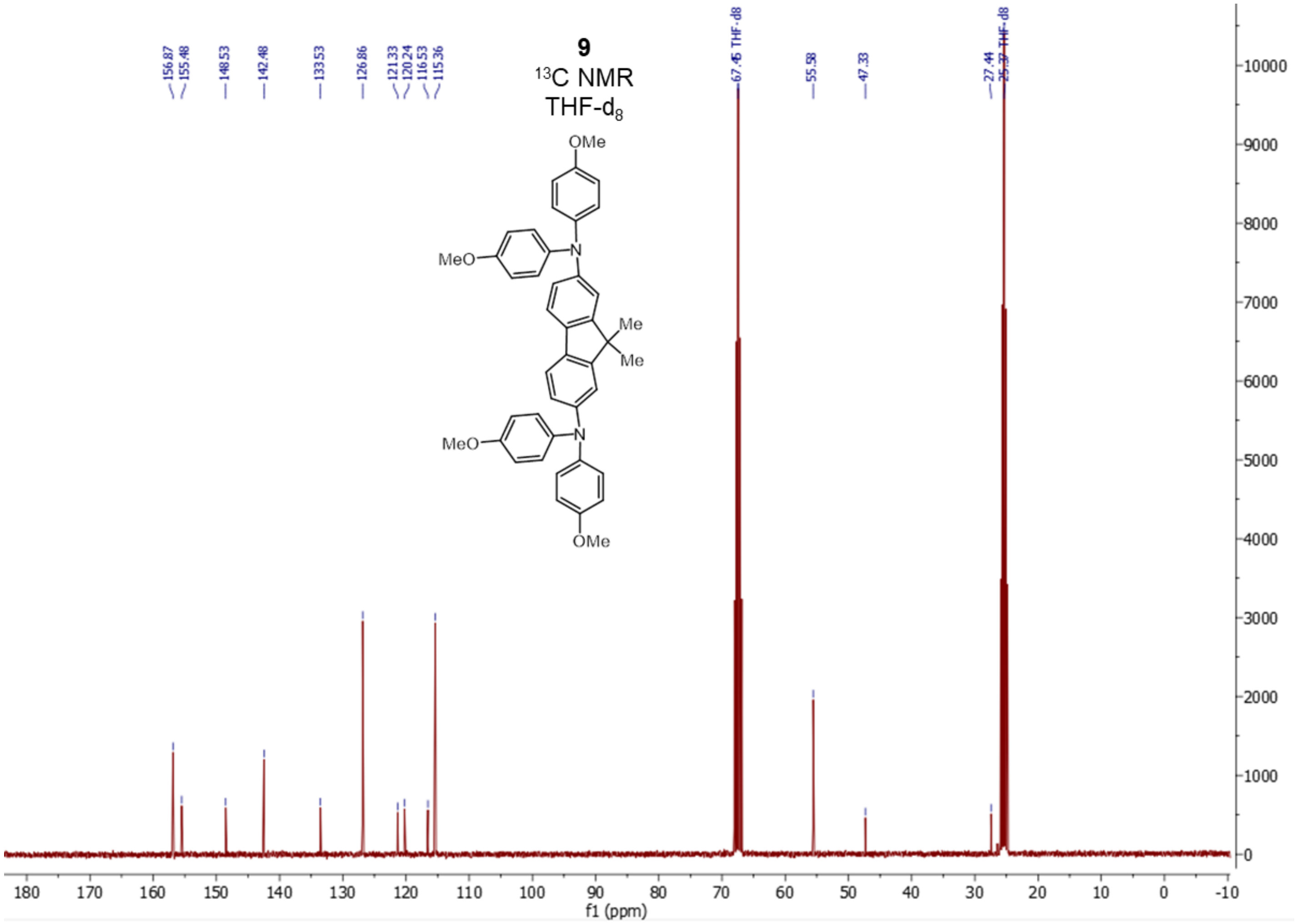

Supplementary Figure 34. ^{13}C NMR (75 MHz, THF-d_8) spectrum of **9**.

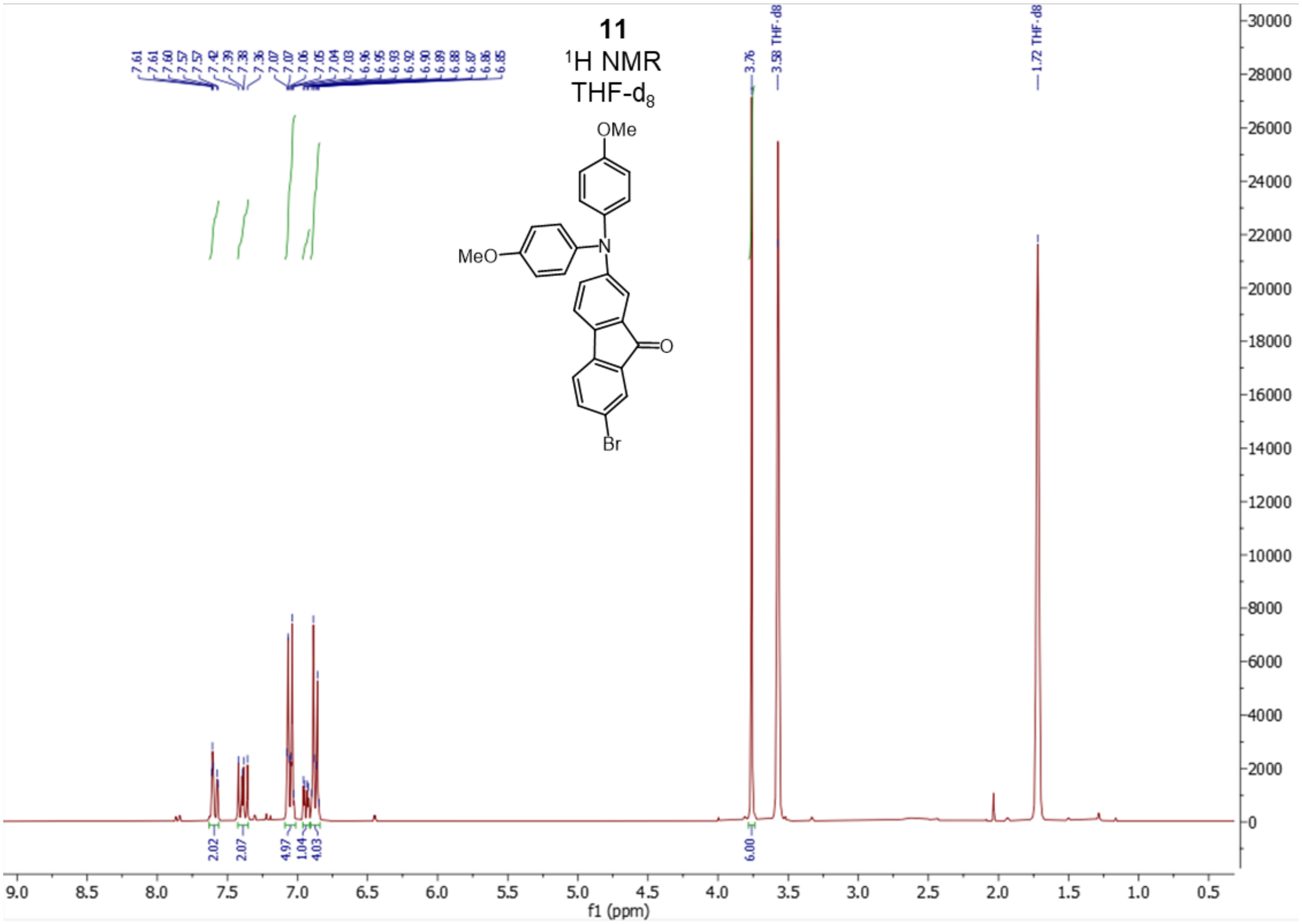

Supplementary Figure 35. ^{1}H NMR (300 MHz, THF-d_8) spectrum of **11**.

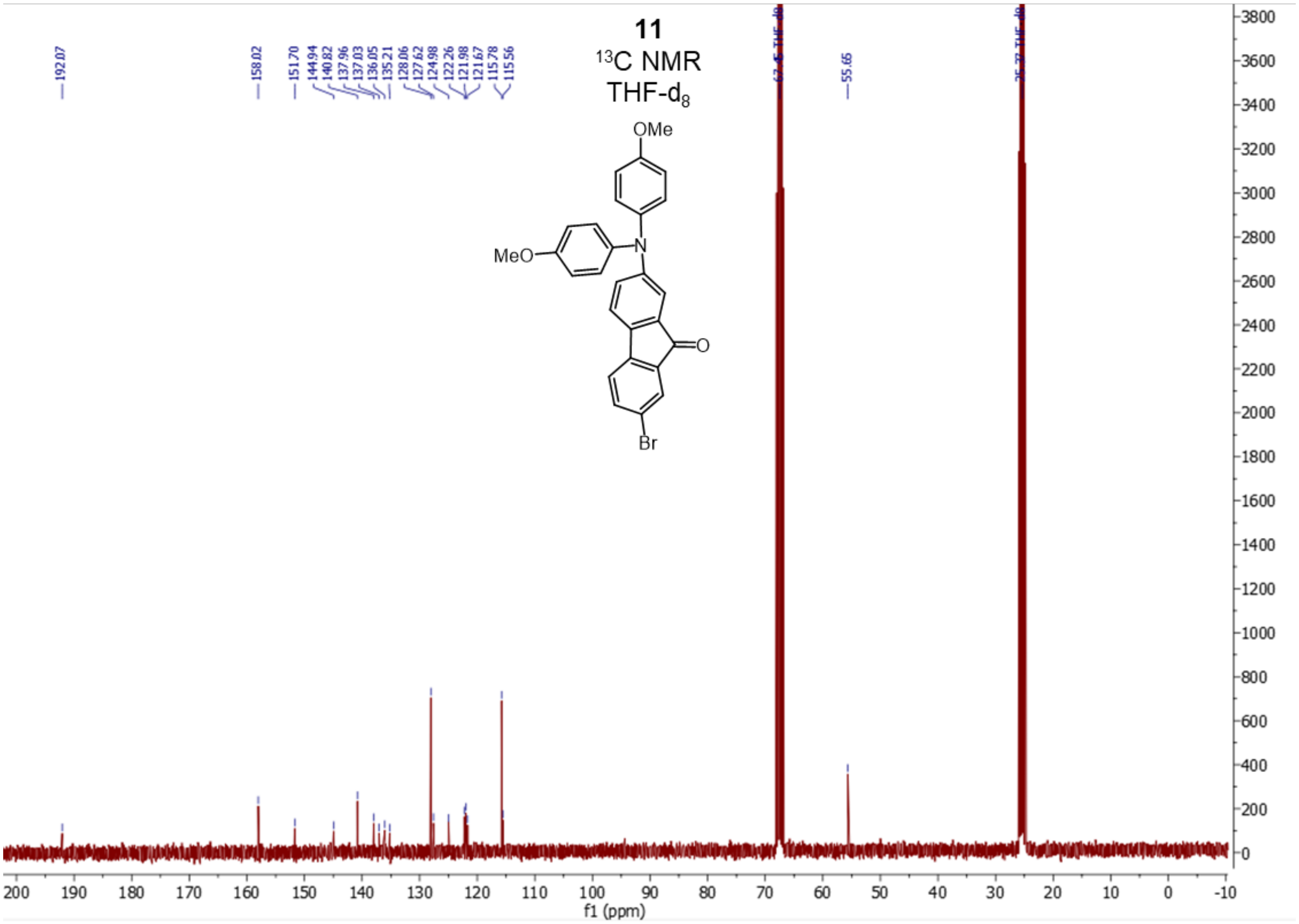

Supplementary Figure 36. ^{13}C NMR (75 MHz, THF-d_8) spectrum of **11**.

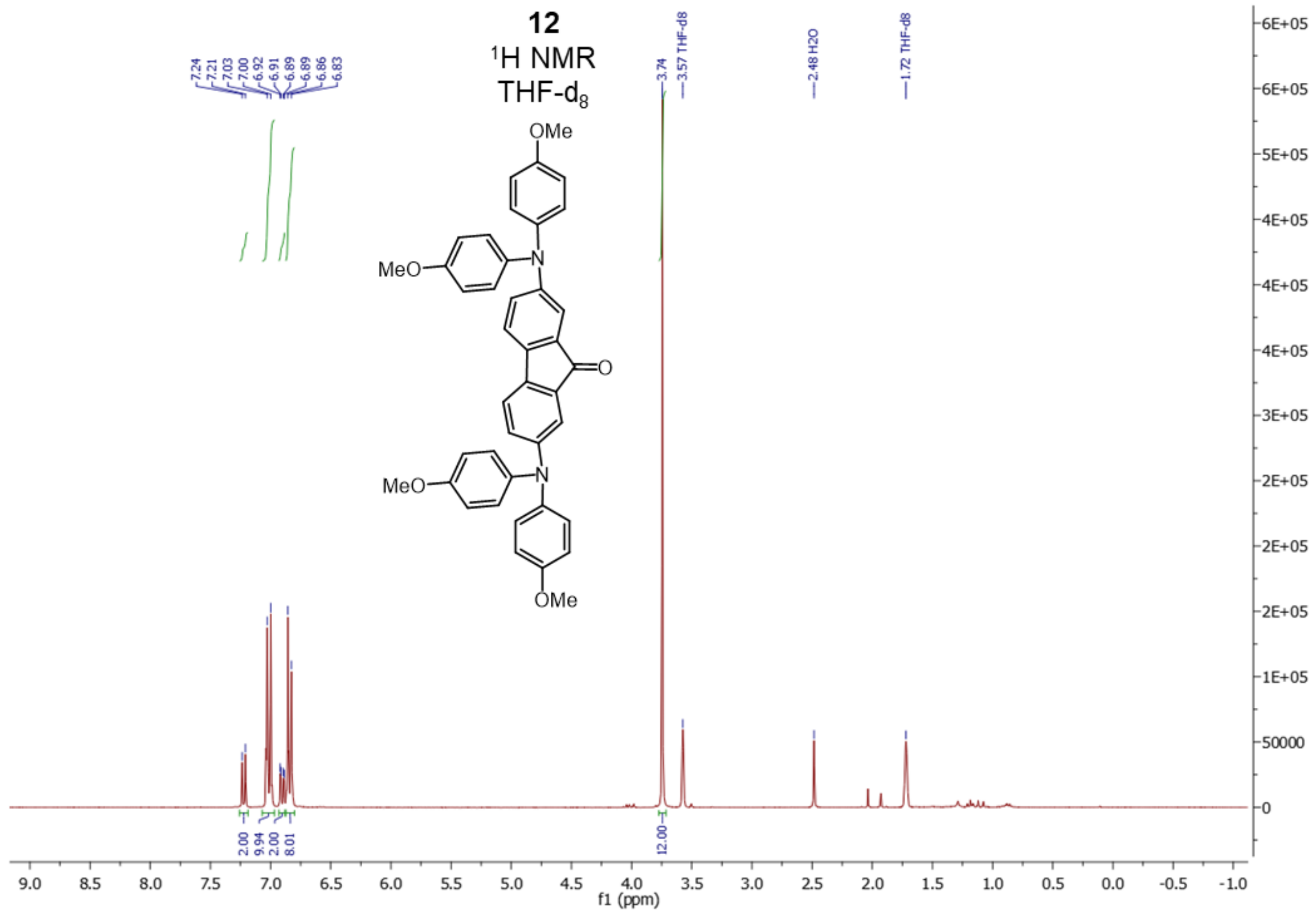

Supplementary Figure 37. ^{1}H NMR (300 MHz, THF-d_8) spectrum of **12**.

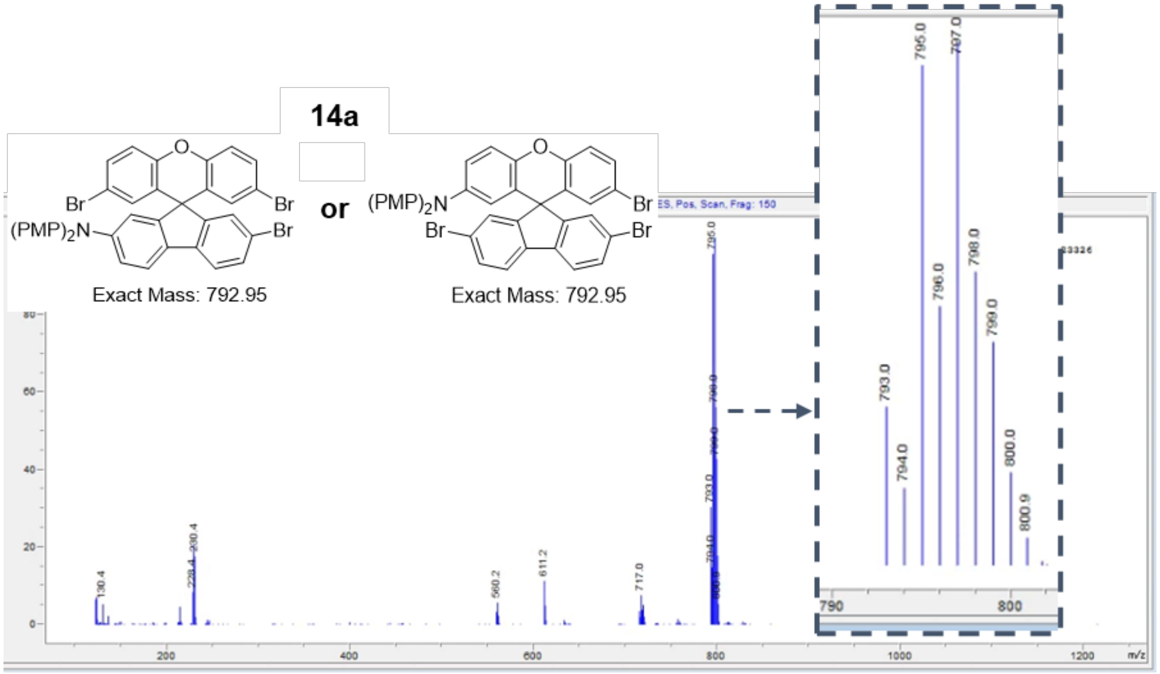

Supplementary Figure 38. LRMS of **14a** (exact mass: 792.95 m/z, found: 793.0 m/z).

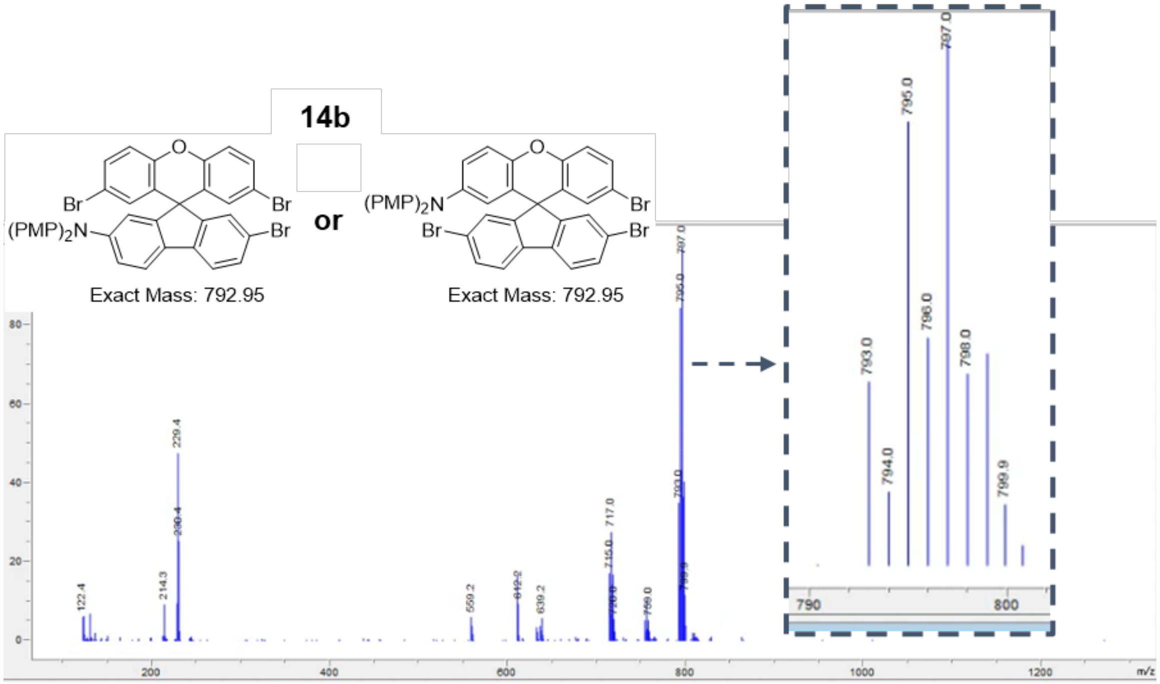

Supplementary Figure 39. LRMS of **14b** (exact mass: 792.95 m/z, found: 793.0 m/z).

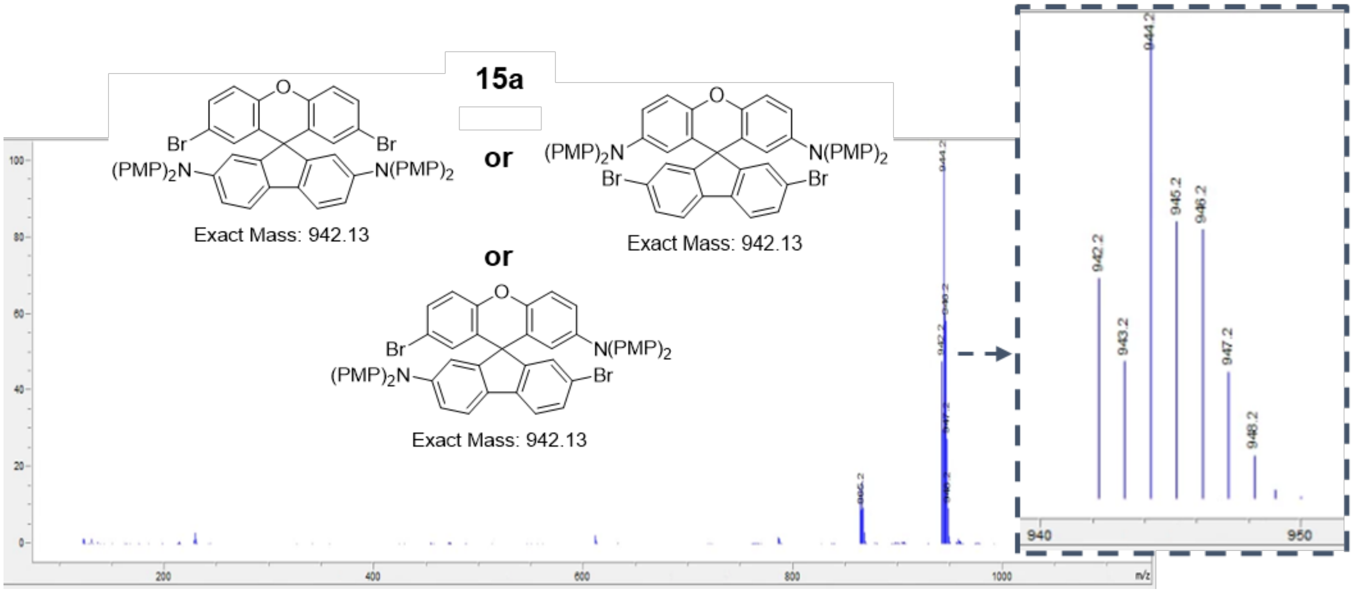

Supplementary Figure 40. LRMS of **15a** (exact mass: 942.13 m/z, found: 942.2 m/z).

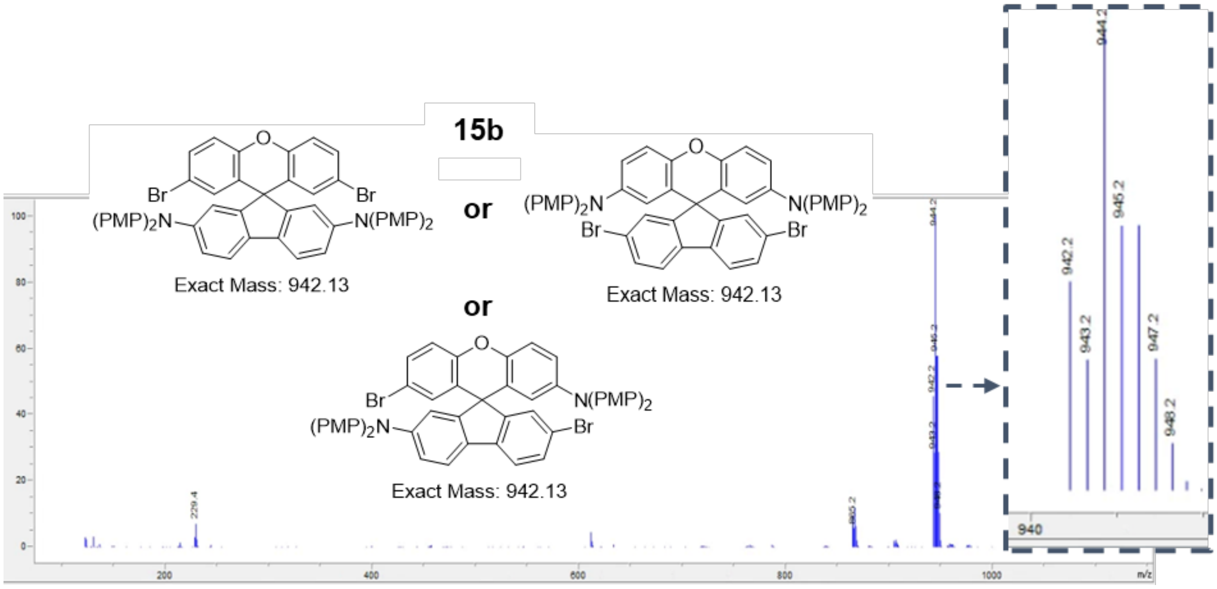

Supplementary Figure 41. LRMS of **15b** (exact mass: 942.13 m/z, found: 942.2 m/z).

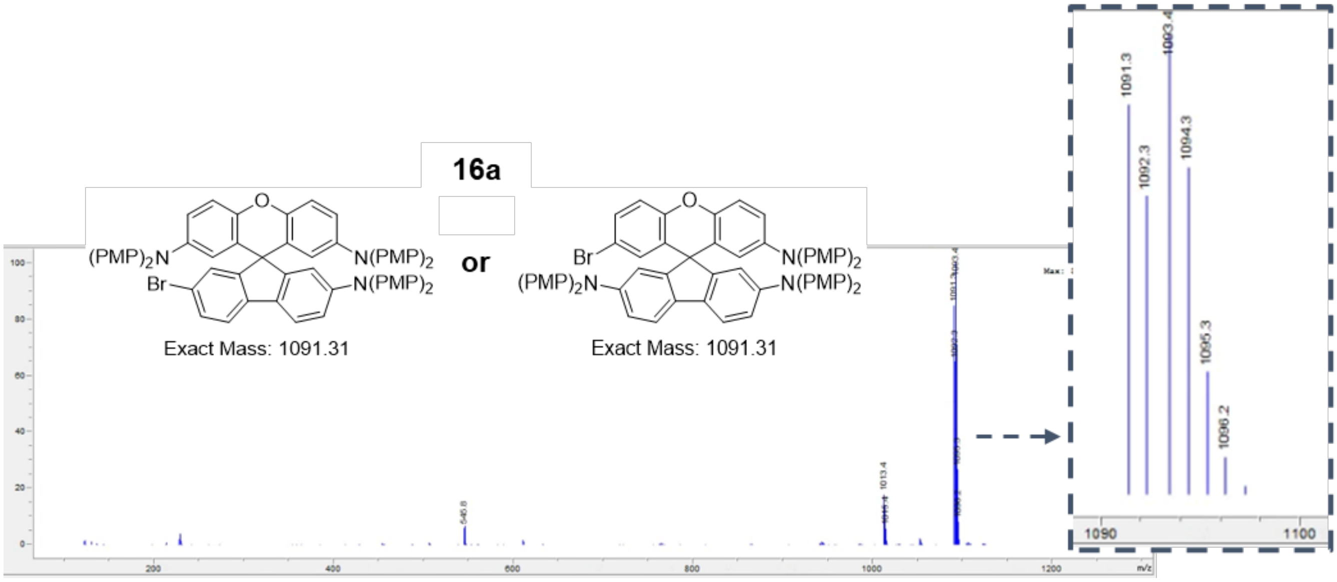

Supplementary Figure 42. LRMS of **16a** (exact mass: 1091.31 m/z, found: 1091.3 m/z).

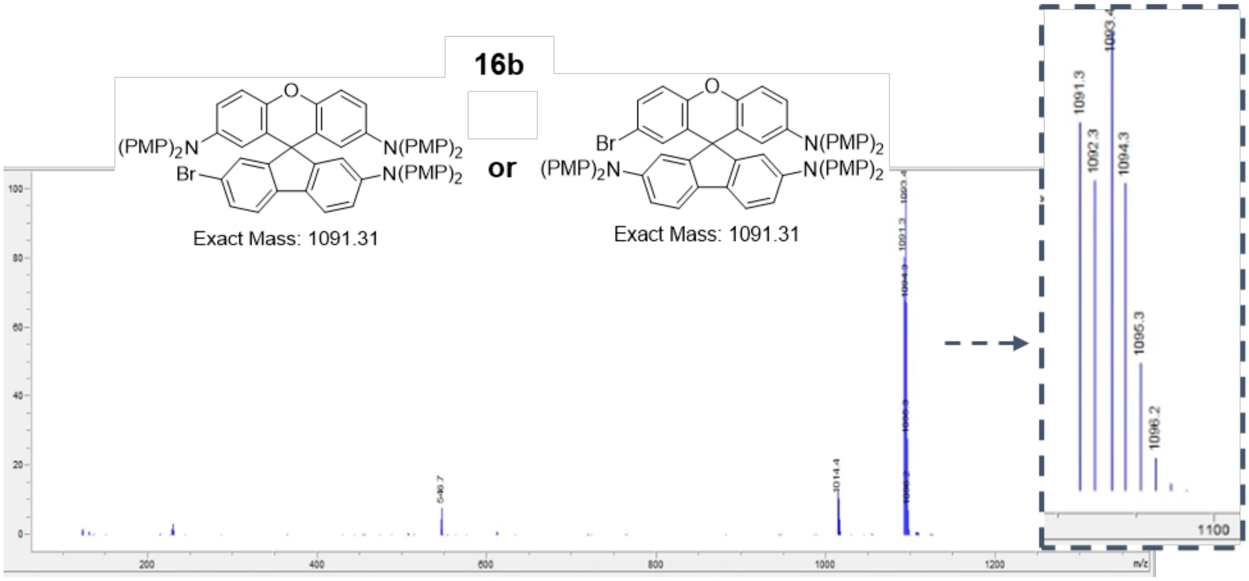

Supplementary Figure 43. LRMS of **16b** (exact mass: 1091.31 m/z, found: 1091.3 m/z).

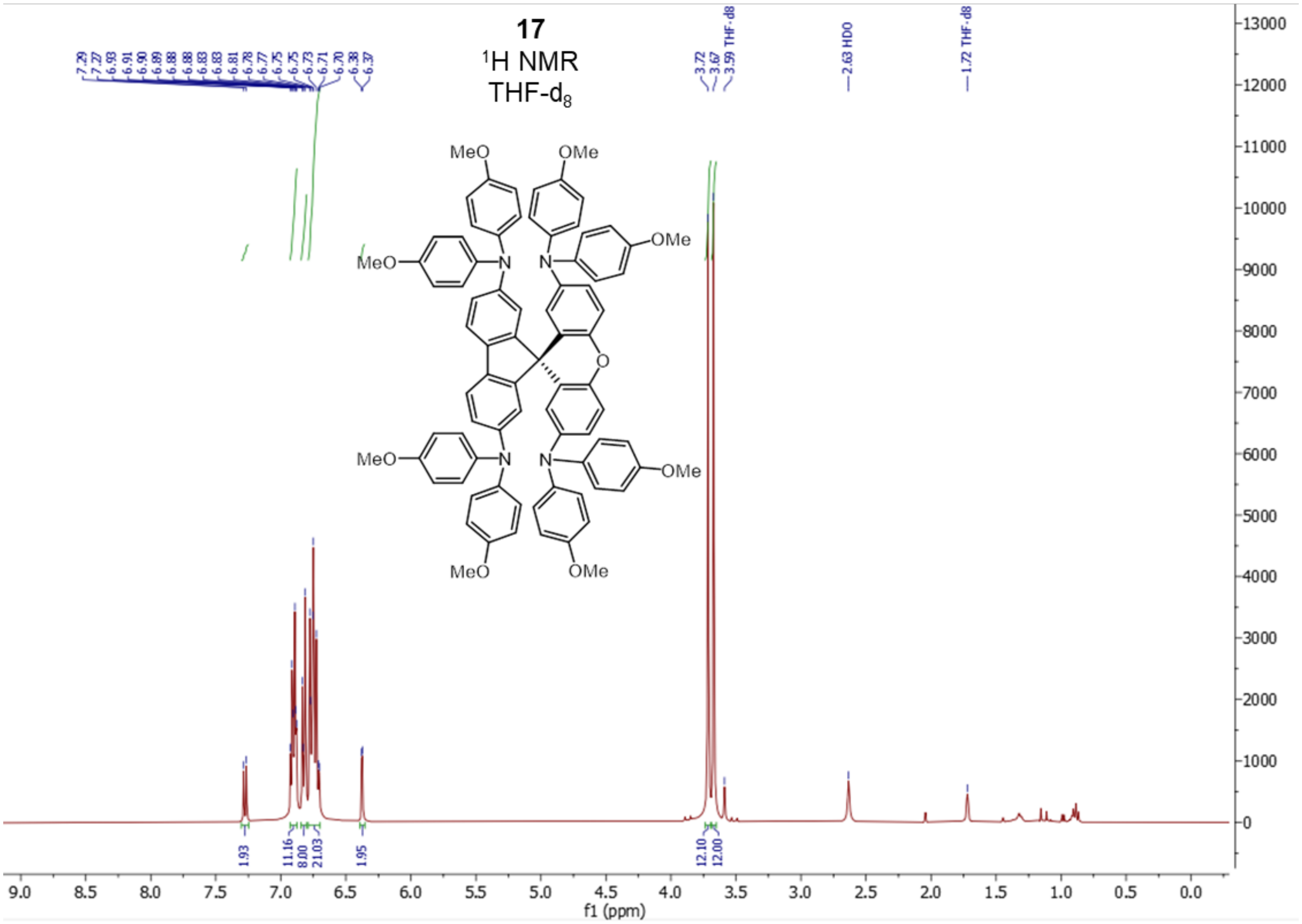

Supplementary Figure 44. ^{1}H NMR (400 MHz, THF-d_8) spectrum of **17**.

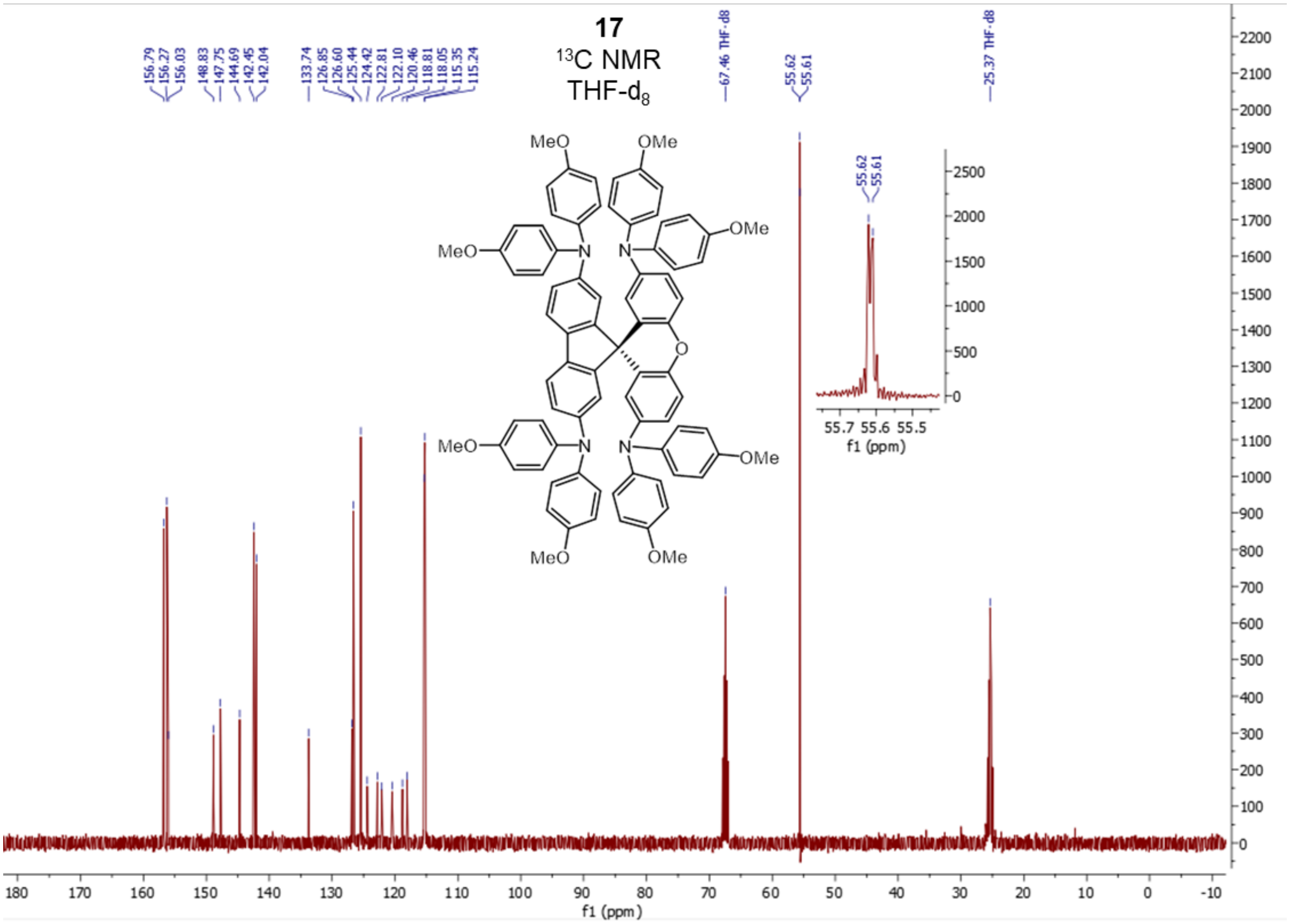

Supplementary Figure 45. ^{13}C NMR (101 MHz, THF-d_8) spectrum of **17**.

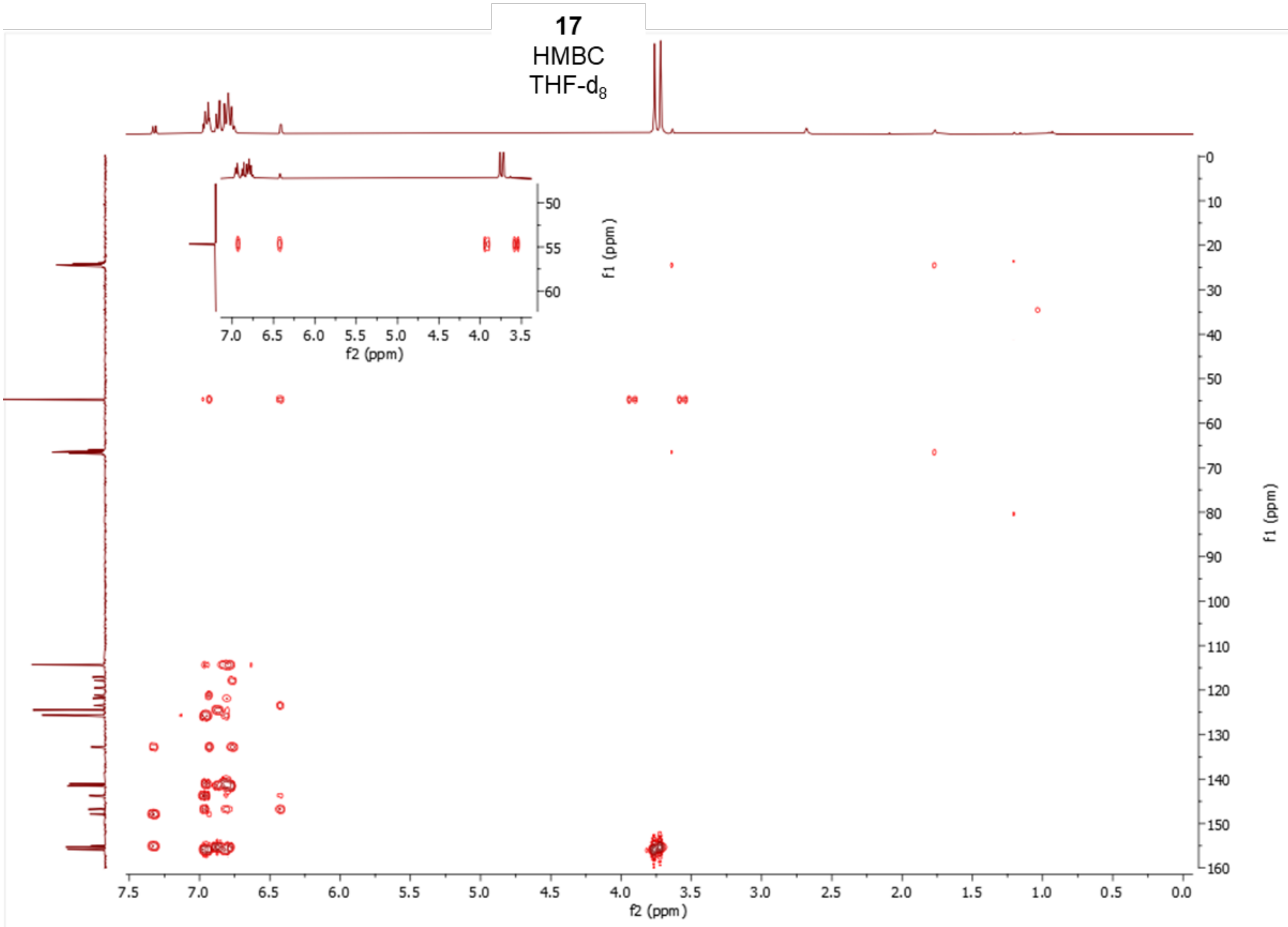

Supplementary Figure 46. HMBC NMR (^{1}H: 400 MHz, ^{13}C: 101 MHz, THF-d_8) spectrum of **17**.

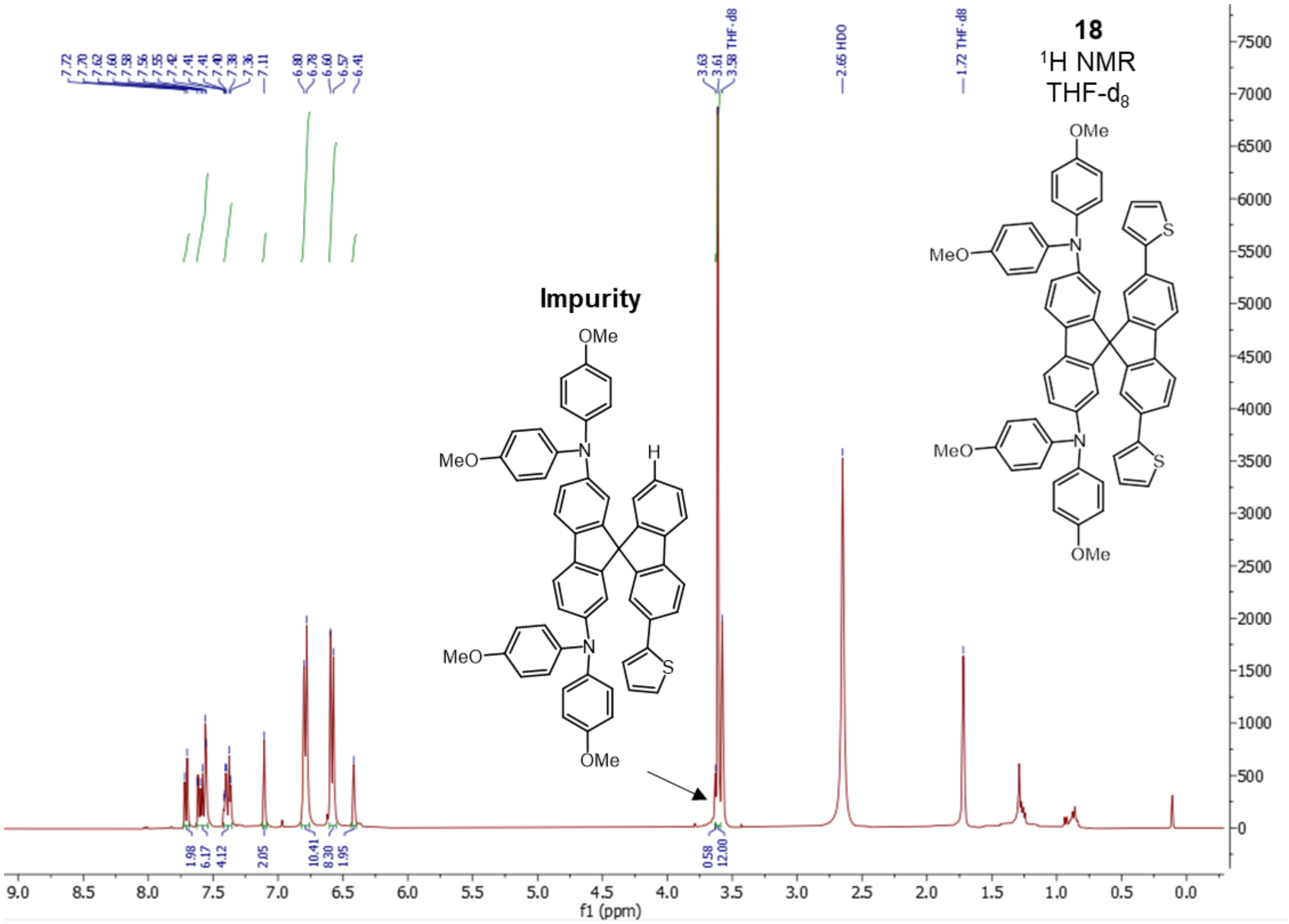

Supplementary Figure 47. ^{1}H NMR (600 MHz, THF-d_8) spectrum of **18** with a minor impurity.

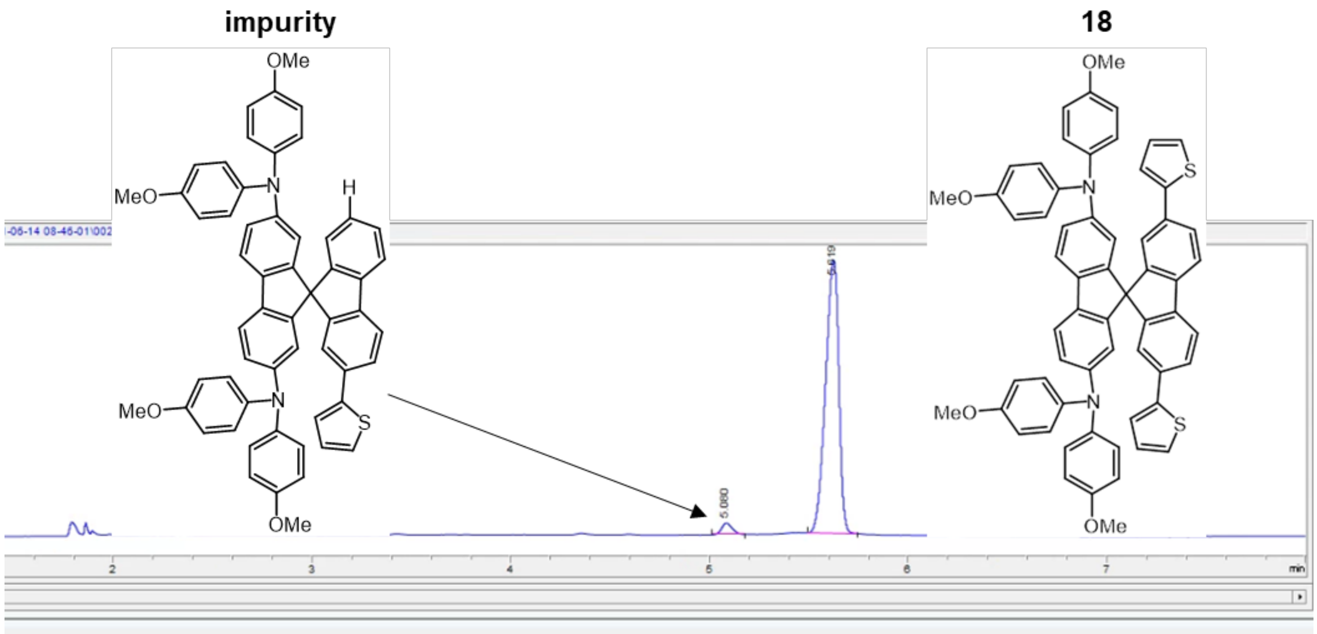

#	Time	Type	Area	Height	Width	Area%	Symmetry
1	5.08	BB	45.1	12.3	0.0559	3.156	0.785
2	5.619	BB	1385.5	296.1	0.0736	96.844	1.485

Supplementary Figure 48. HPLC chromatogram showing the composition of **18** (96.8% by HPLC peak area) with a minor impurity (3.2% by HPLC peak area).

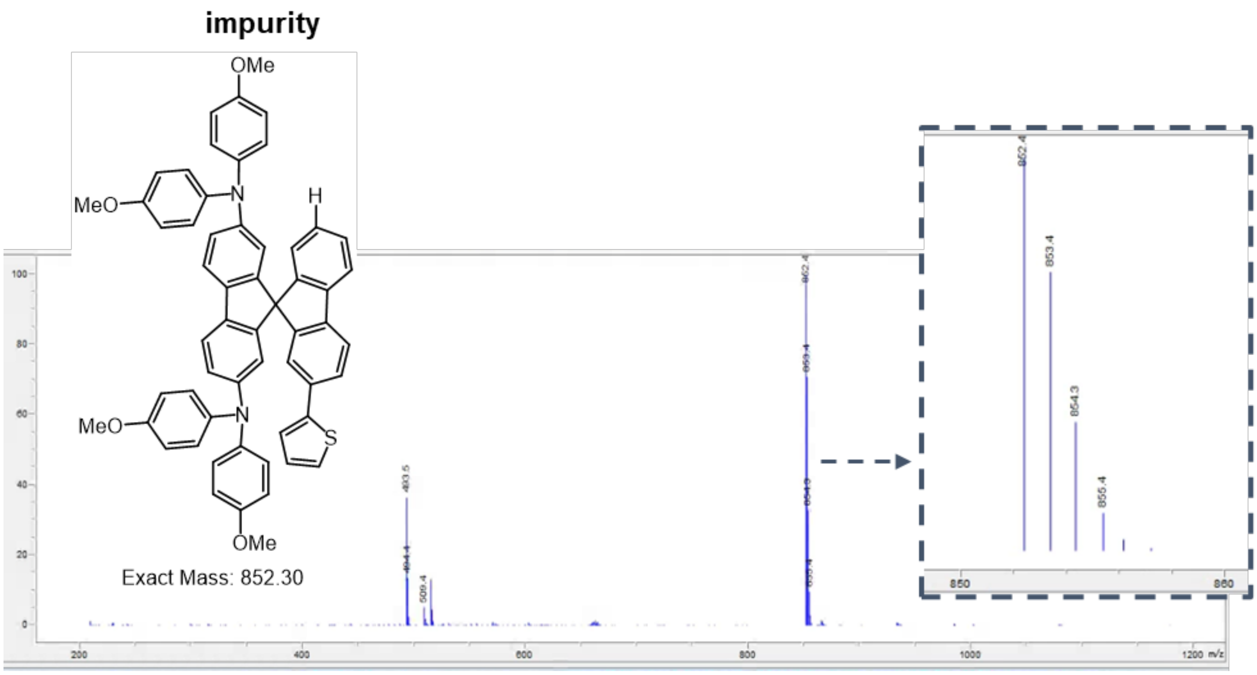

Supplementary Figure 49. LRMS of the impurity contaminant of **18** (exact mass of impurity: 852.30 m/z, found: 852.4 m/z).

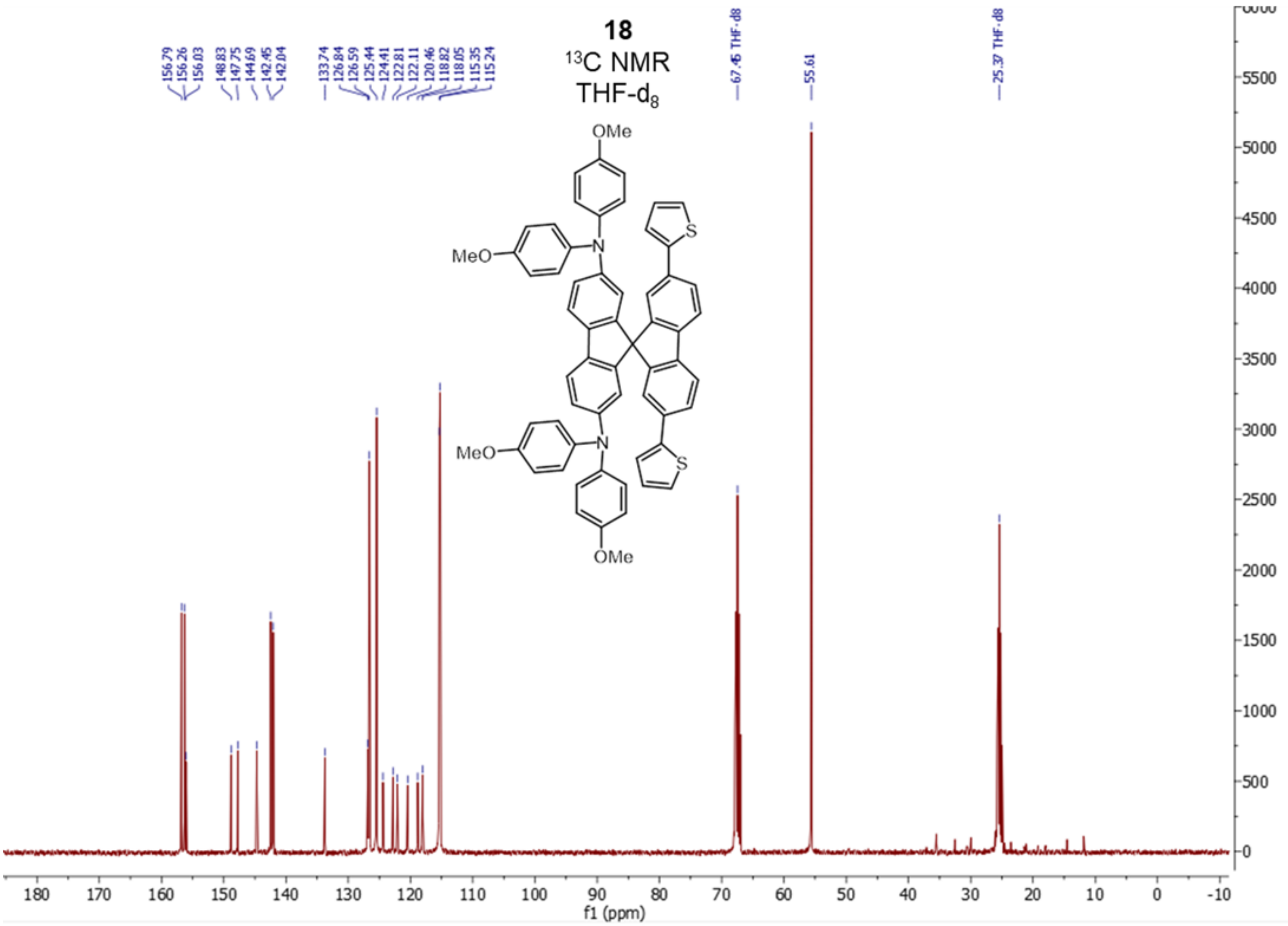

Supplementary Figure 50. ^{13}C NMR (101 MHz, THF-d_8) spectrum of **18**.

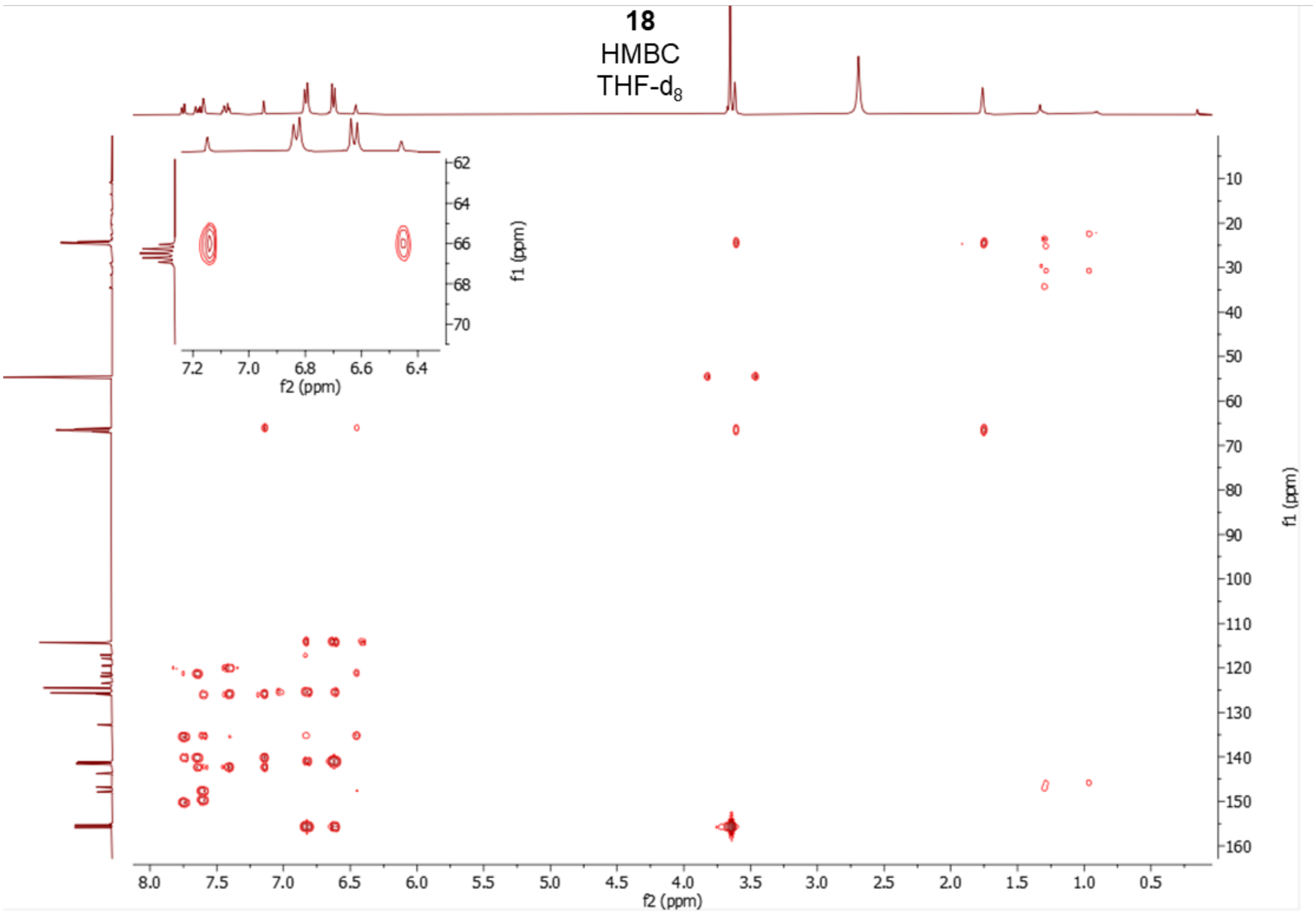

Supplementary Figure 51. HMBC NMR (^{1}H: 400 MHz, ^{13}C: 101 MHz, THF-d_8) spectrum of **18**.

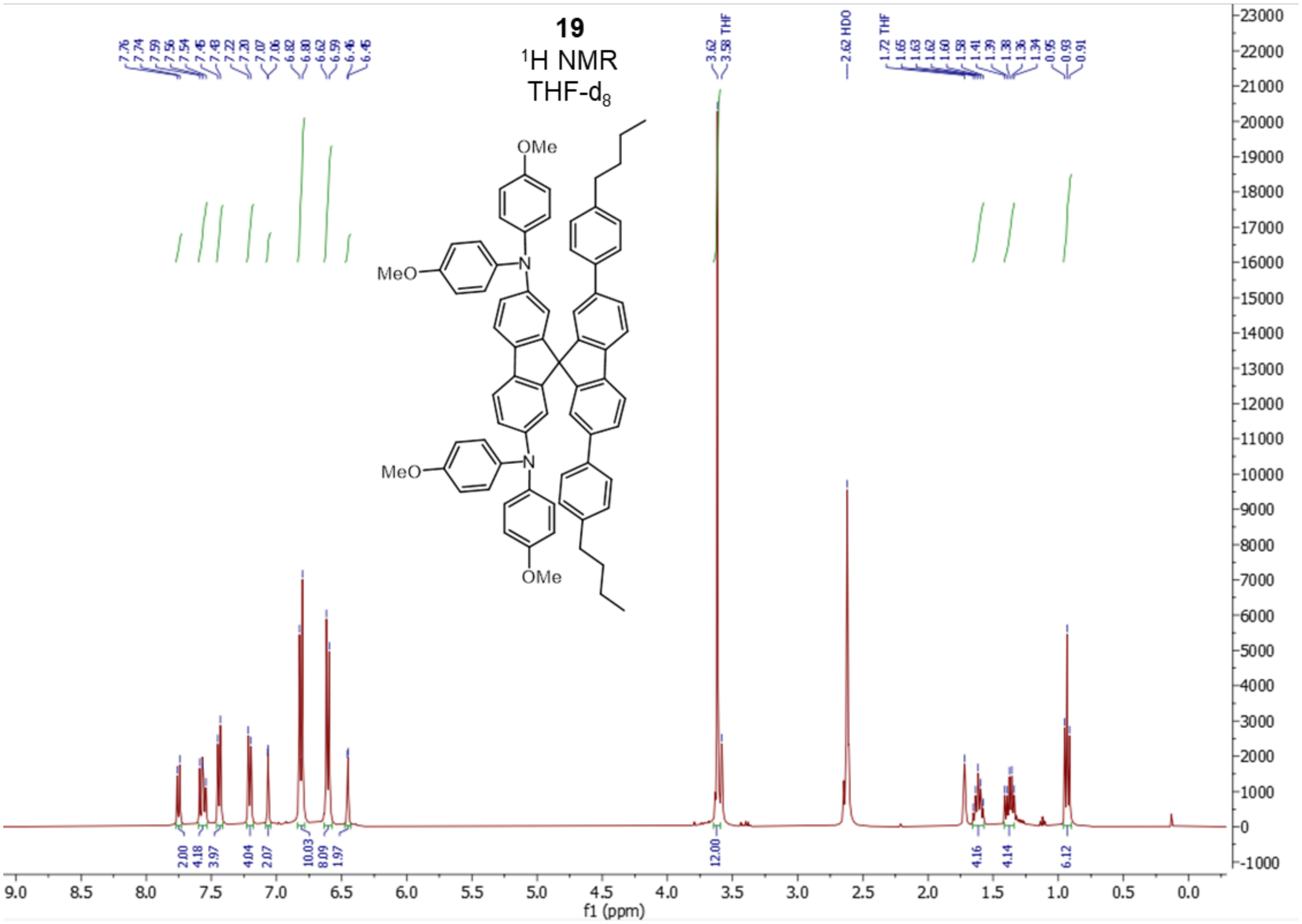

Supplementary Figure 52. ^{1}H NMR (400 MHz, THF-d_8) spectrum of **19**.

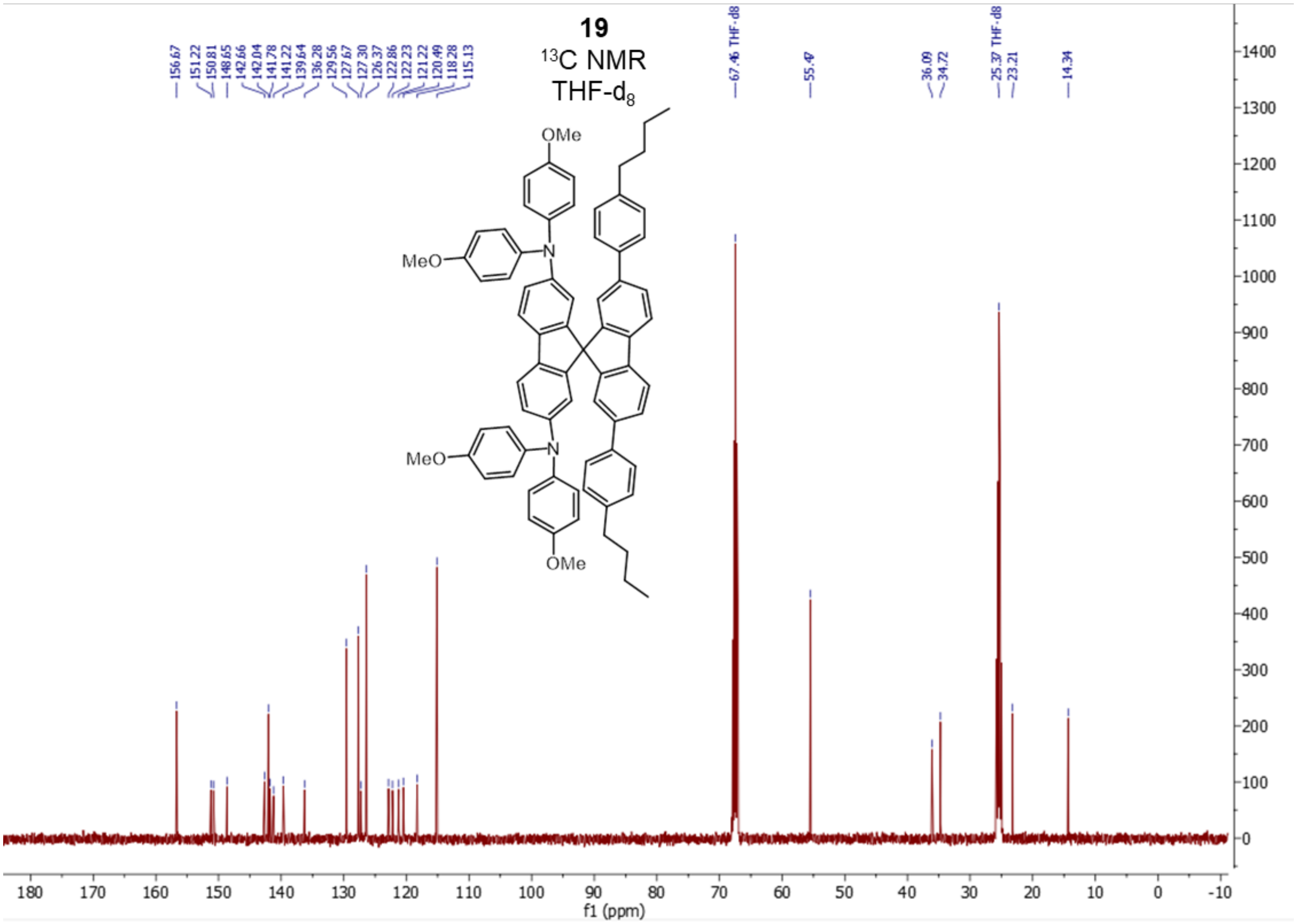

Supplementary Figure 53. ^{13}C NMR (101 MHz, THF-d_8) spectrum of **19**.

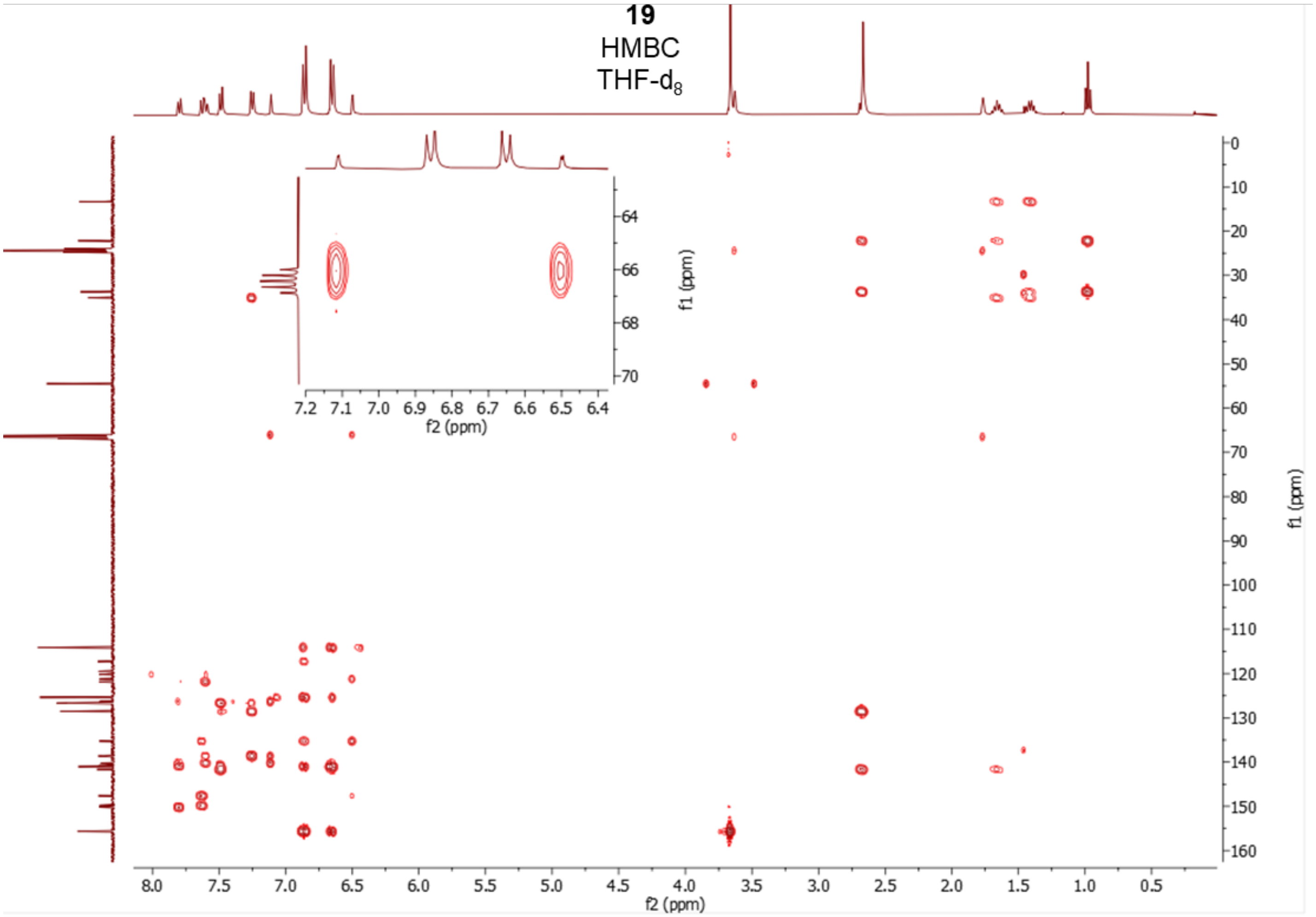

Supplementary Figure 54. HMBC NMR (^{1}H: 400 MHz, ^{13}C: 101 MHz, THF-d_8) spectrum of **19**.

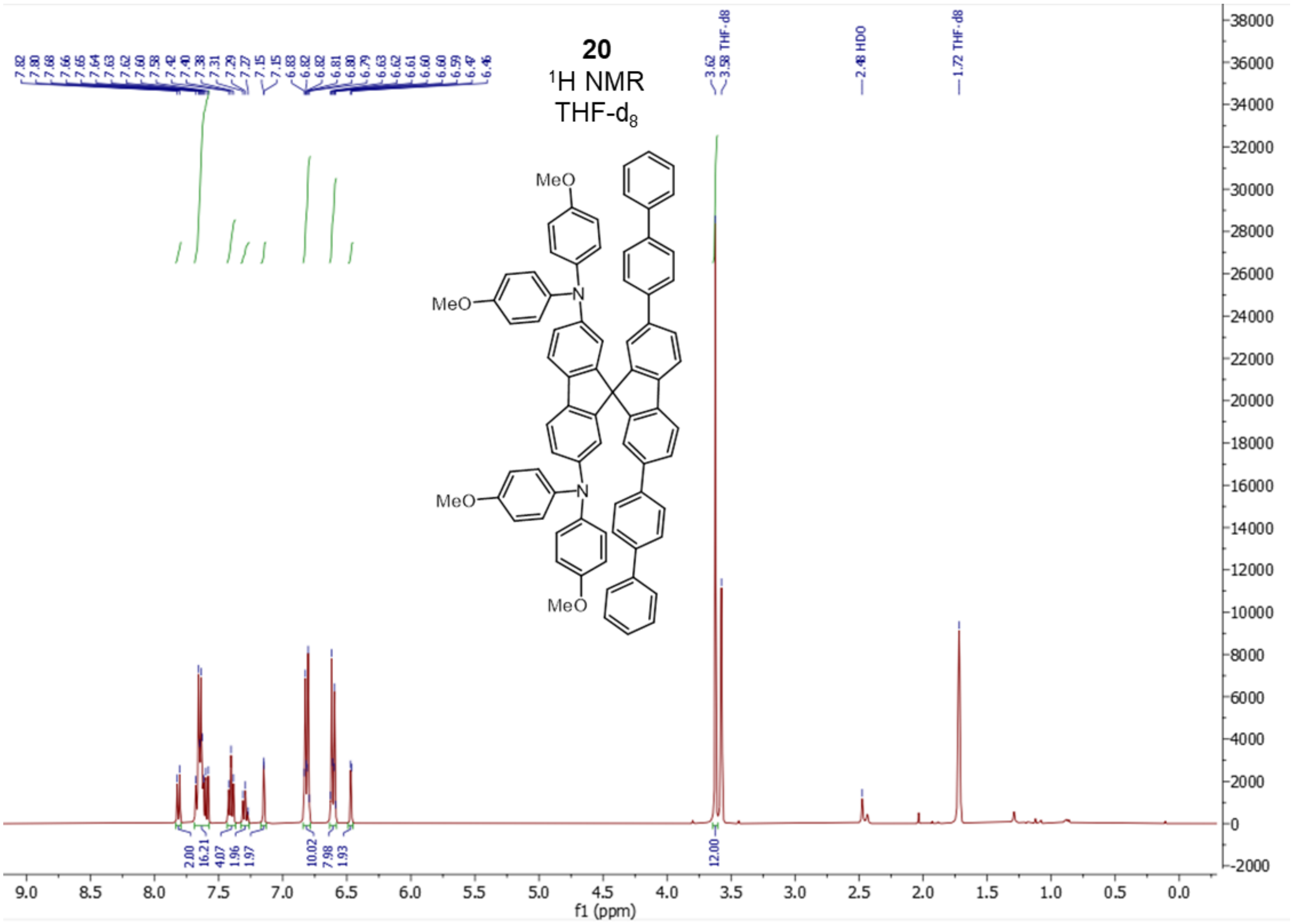

Supplementary Figure 55. ^{1}H NMR (400 MHz, THF-d_8) spectrum of **20**.

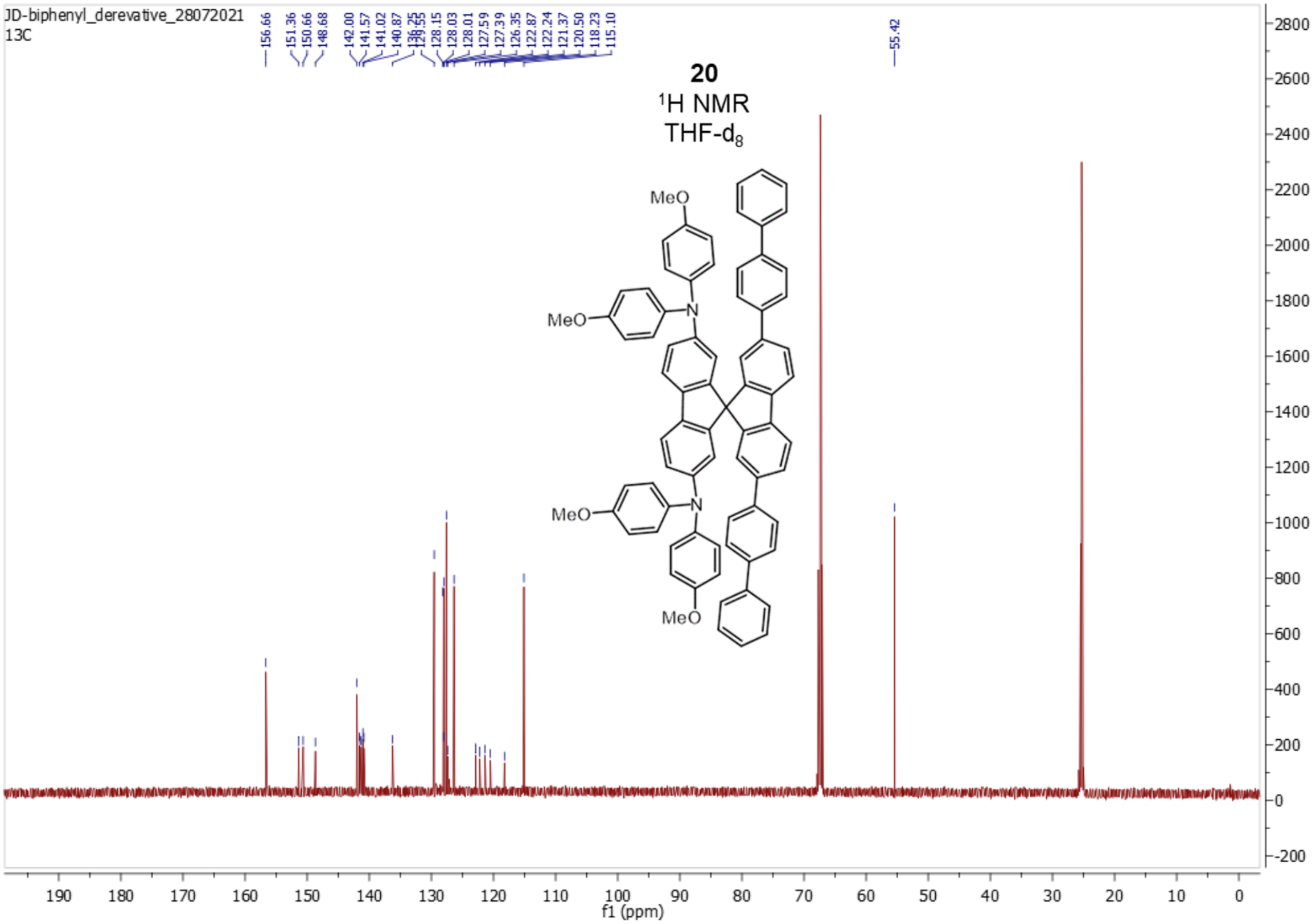

Supplementary Figure 56. ^{13}C NMR (101 MHz, THF-d$_8$) spectrum of **20**.

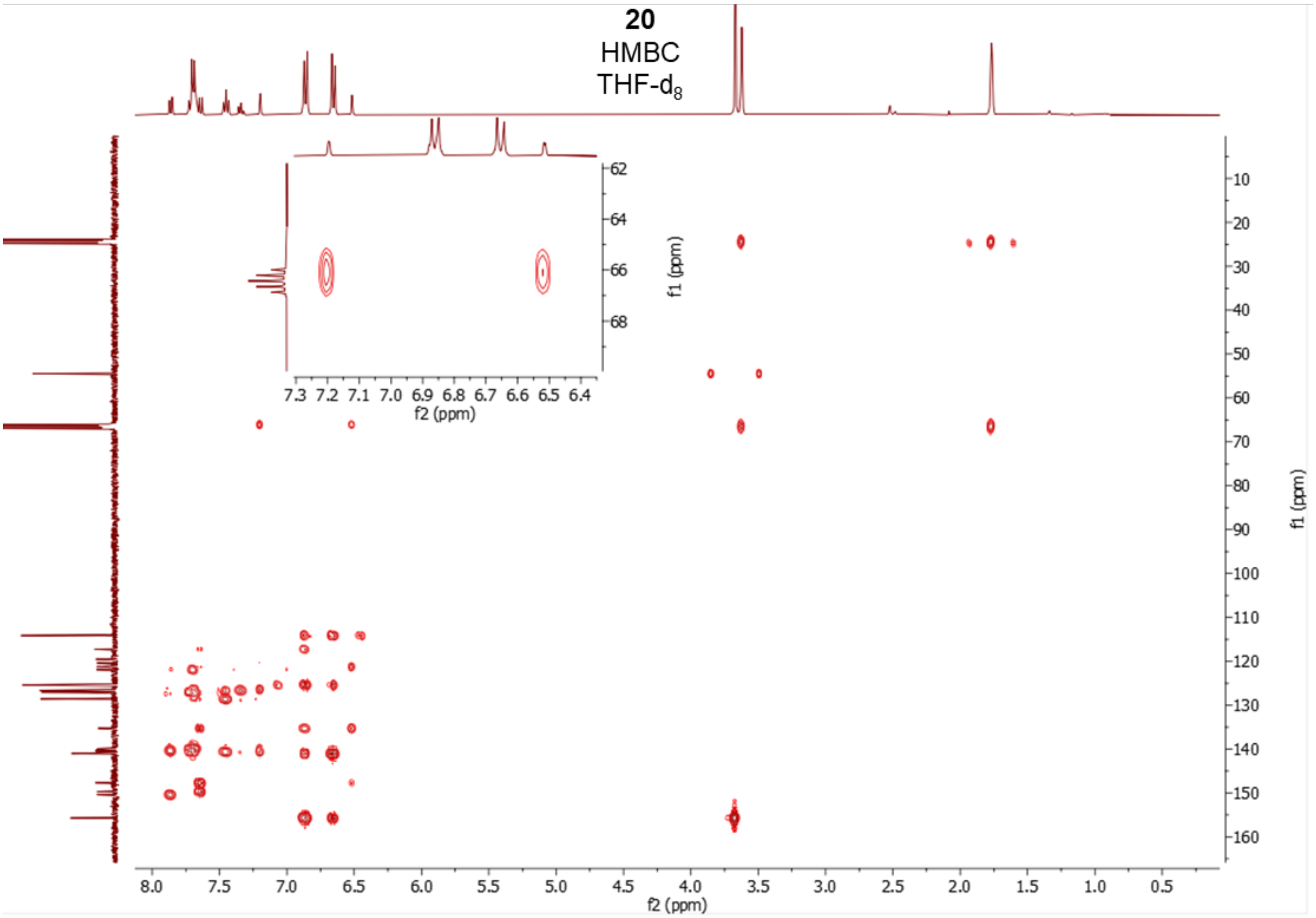

Supplementary Figure 57. HMBC NMR (^{1}H: 400 MHz, ^{13}C: 101 MHz, THF-d_8) spectrum of **20**.

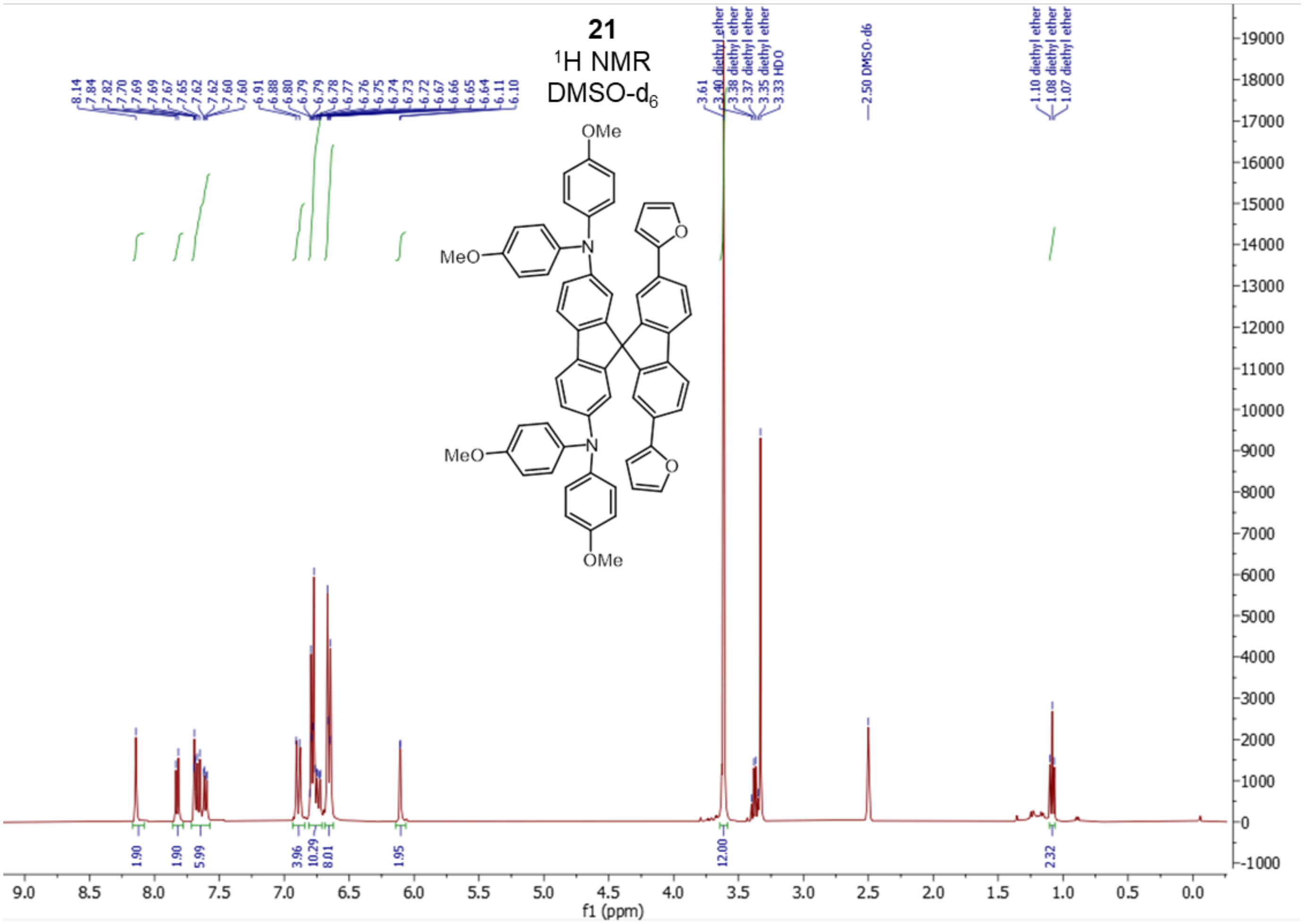

Supplementary Figure 58. ^{1}H NMR (400 MHz, DMSO-d$_6$) spectrum of **21** with diethyl ether contaminant.

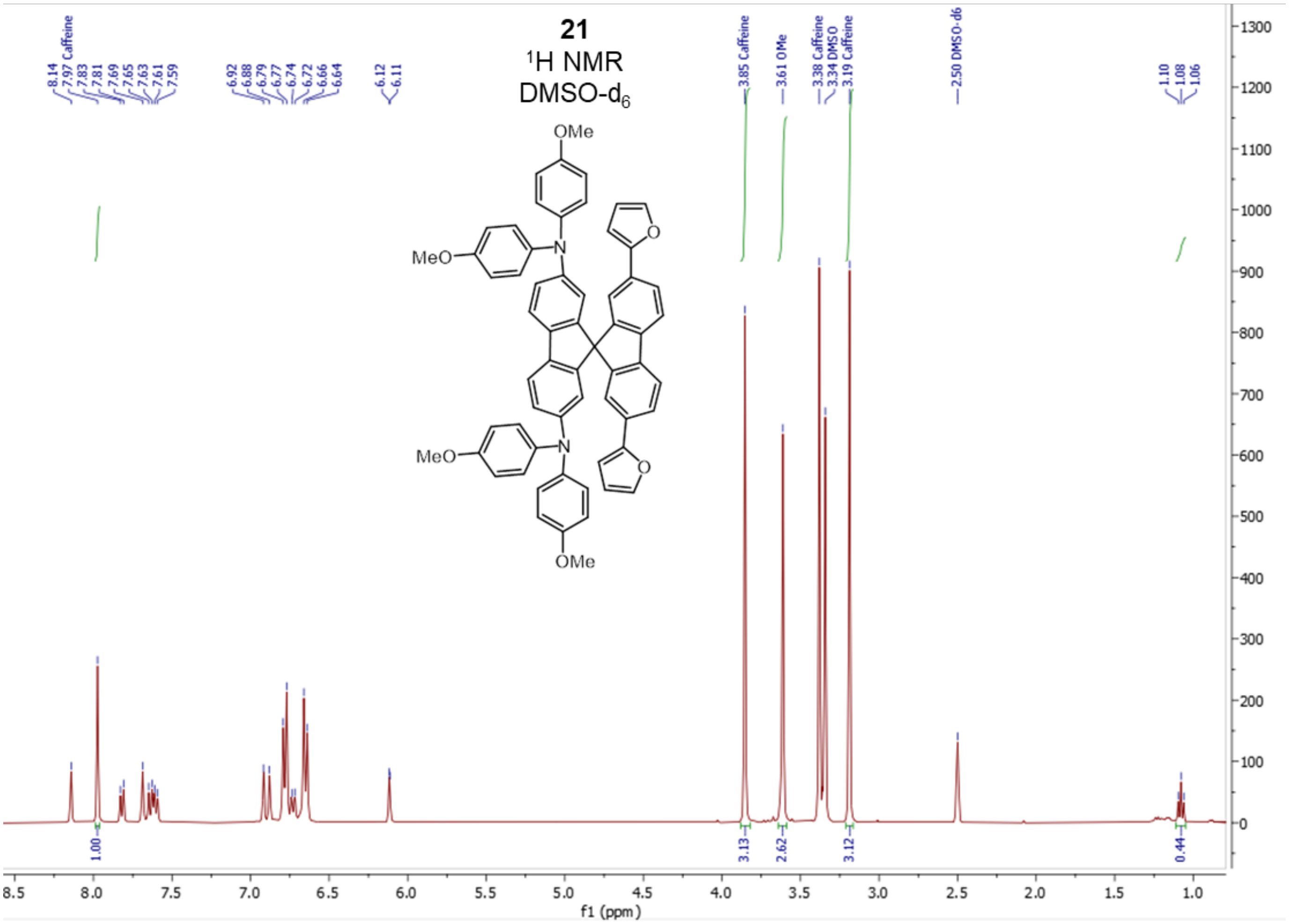

Supplementary Figure 59. ^{1}H NMR (400 MHz, DMSO-d_6) spectrum of **21** with diethyl ether contaminant and caffeine as an internal standard.

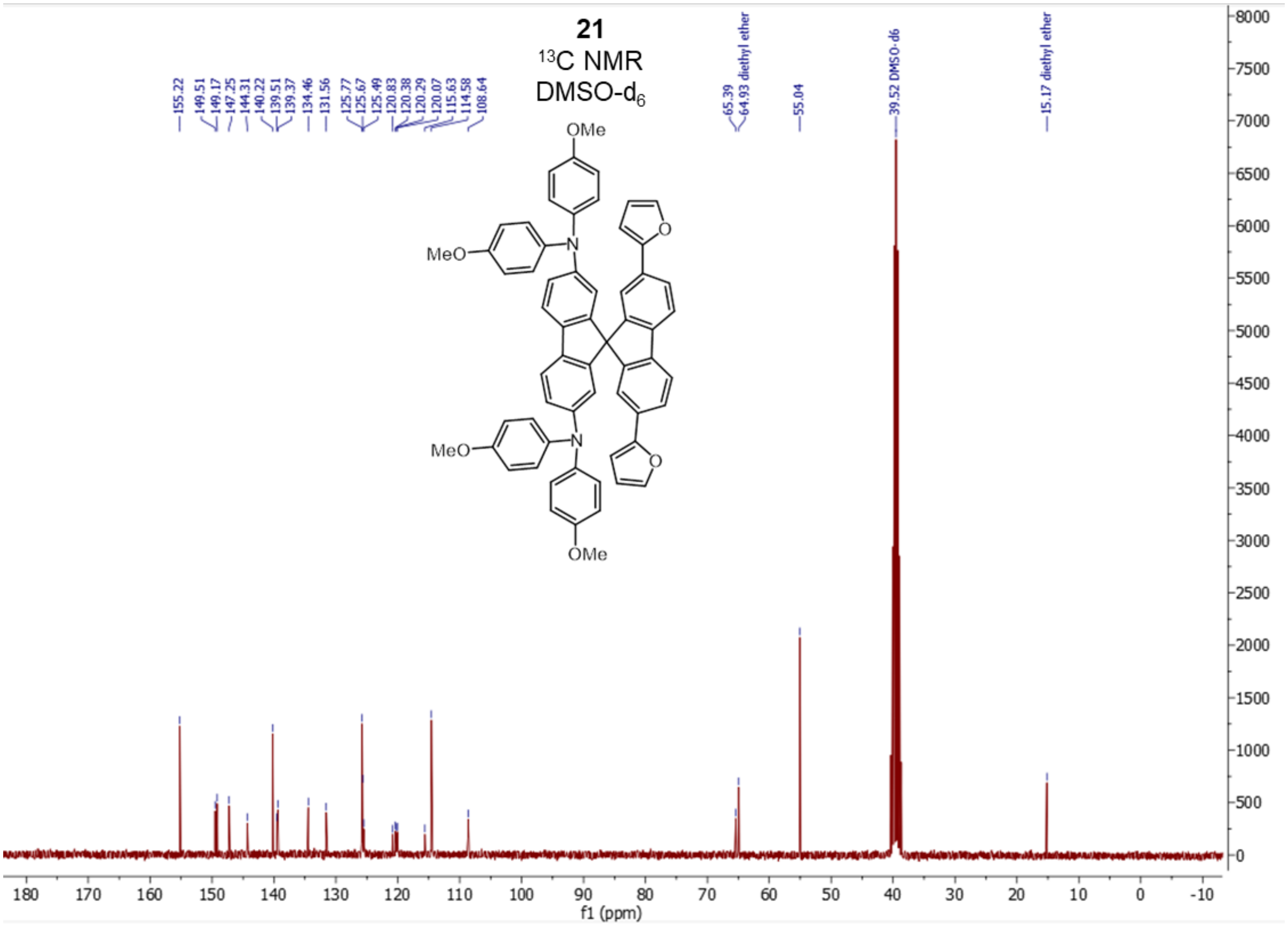

Supplementary Figure 60. ^{13}C NMR (101 MHz, DMSO-d_6) spectrum of **21** with diethyl ether contaminant.

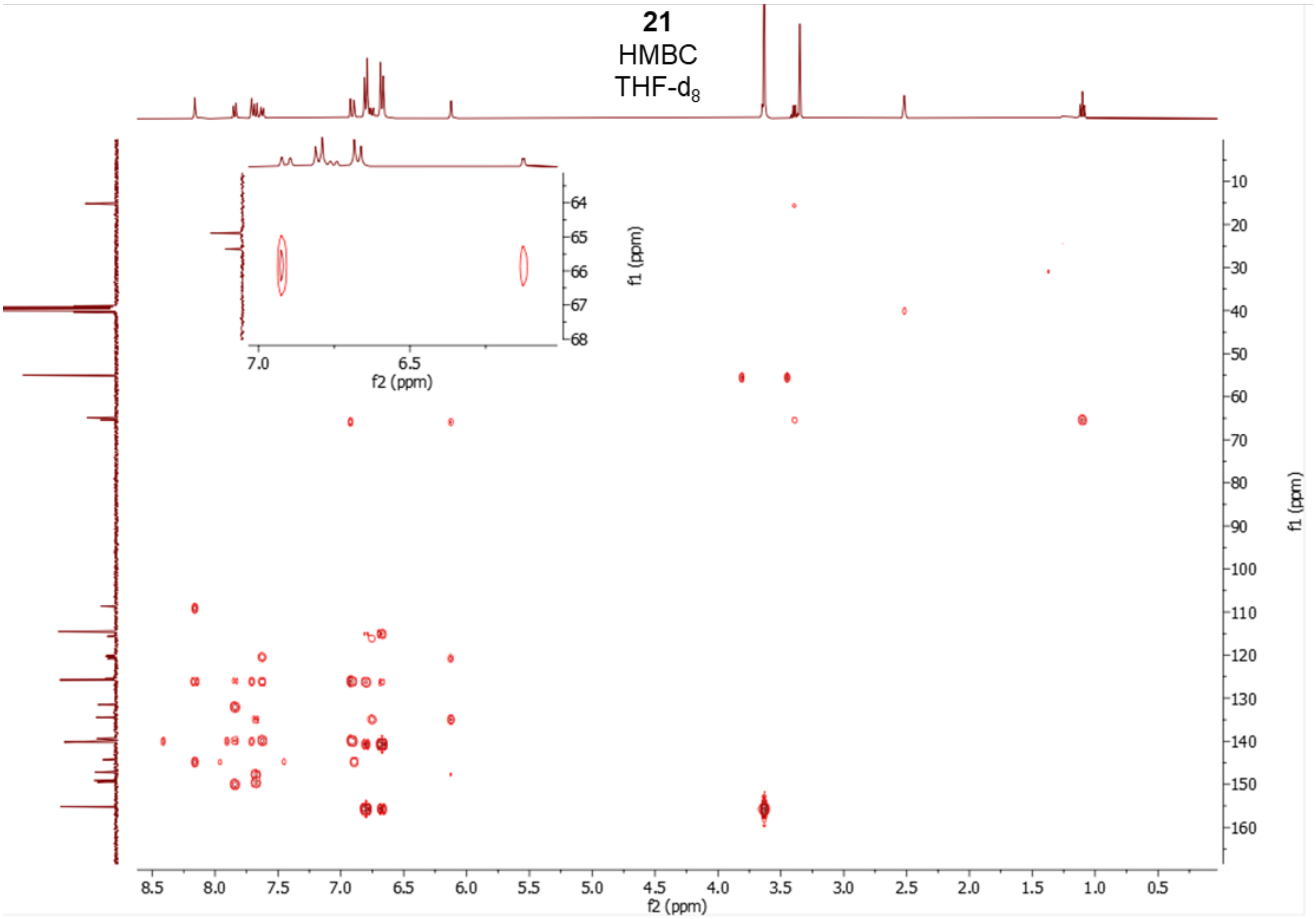

Supplementary Figure 61. HMBC NMR (^{1}H: 400 MHz, ^{13}C: 101 MHz, DMSO-d_6) spectrum of **21**.

Supplementary References

1) Chiykowski, V. A., Cao, Y., Tan, H., Tabor, D. P., Sargent, E. H., Aspuru-Guzik, A., and Berlinguette, C. P. Precise Control of Thermal and Redox Properties of Organic Hole-Transport Materials. Angew. Chem. Int. Ed. 57, 15529-15533 (2018)

2) Malig, T. C., Yunker, L. P. E., Steiner, S., and Hein, J. E. Online High-Performance Liquid Chromatography Analysis of Buchwald-Hartwig Aminations from within an Inert Environment. ACS Catal. 10, 13236-13244 (2020)

3) Blackmond, D. Reaction Progress Kinetic Analysis: A Powerful Methodology for Mechanistic Studies of Complex Catalytic Reactions. *Angew. Chem. Int. Ed.***44**, 4302-4320 (2005)

4) Nielsen, C.D.T., Bures, J. Visual Kinetic Analysis. *Chem. Sci.* **10**, 348-353 (2019)

5) Malig, T. C. Leveraging automation to elucidate reaction mechanisms. Ph.D. Dissertation, University of British Columbia, Vancouver, British Columbia, (2020).

6) Jeon, N. J., Lee, H. G., Kim, Y. C., Seo, J., Noh, J. H., Lee, J., and Seok, S. I.*o*-Methoxy Substituents in Spiro-OMeTAD for Efficient Inorganic-Organic Hybrid Perovskite Solar Cells. *J. Am. Chem. Soc.* **136**, 7837-7840 (2014)

7) Liu, F., Tu, Z., Fan, Y., Li, Q., and Li, Z. Spiro-Structure: A Good Approach to Achieve Mechanoluminescence Property. *ACS Omega* **4**, 18609-18615 (2019)

8) Fang, L., Zheng, A., Ren, M., Xie, X., and Wang, P. Unraveling the Structure-Property Relationship of Molecular Hole-Transporting Materials for Perovskite Solar Cells. *ACS Appl. Mater. Interfaces,* **11**, 39001-39009 (2019)
9) Reinfelds, M., von Cosel, J., Falahati, K., Hamerla, C., Slanina, T., Burghardt, I., and Heckel, A. A New Photocage Derived from Fluorene. *Chem. Eur. J.* **24**, 13026-13035 (2018)
10) Tabor, D. P., Chiykowski, V. A., Friederich, P., Cao, Y., Dvorak, D. J., Berlinguette, C. P., and Aspuru-Guzik, A. Design rules for high mobility xanthene-based hole transport materials. *Chem. Sci.* **10**, 8360-8366 (2019)
